# Molecular dynamics and in silico mutagenesis on the reversible inhibitor-bound SARS-CoV-2 main protease complexes reveal the role of lateral pocket in enhancing the ligand affinity

**DOI:** 10.1038/s41598-021-86471-0

**Published:** 2021-04-01

**Authors:** Ying Li Weng, Shiv Rakesh Naik, Nadia Dingelstad, Miguel R. Lugo, Subha Kalyaanamoorthy, Aravindhan Ganesan

**Affiliations:** 1 ArGan’s Lab, School of Pharmacy, Faculty of Science, University of Waterloo, Waterloo, ON Canada; 2 Department of Chemistry, Faculty of Science, University of Waterloo, Waterloo, ON Canada

**Keywords:** Computational biology and bioinformatics, Drug discovery

## Abstract

The 2019 novel coronavirus pandemic caused by SARS-CoV-2 remains a serious health threat to humans and there is an urgent need to develop therapeutics against this deadly virus. Recent scientific evidences have suggested that the main protease (M^pro^) enzyme in SARS-CoV-2 can be an ideal drug target due to its crucial role in the viral replication and transcription processes. Therefore, there are ongoing research efforts to identify drug candidates against SARS-CoV-2 M^pro^ that resulted in hundreds of X-ray crystal structures of ligand-bound M^pro^ complexes in the Protein Data Bank (PDB) describing the interactions of different fragment chemotypes within different sites of the M^pro^. In this work, we performed rigorous molecular dynamics (MD) simulation of 62 reversible ligand–M^pro^ complexes in the PDB to gain mechanistic insights about their interactions at the atomic level. Using a total of over 3 µs long MD trajectories, we characterized different pockets in the apo M^pro^ structure, and analyzed the dynamic interactions and binding affinity of ligands within those pockets. Our results identified the key residues that stabilize the ligands in the catalytic sites and other pockets of M^pro^. Our analyses unraveled the role of a lateral pocket in the catalytic site in M^pro^ that is critical for enhancing the ligand binding to the enzyme. We also highlighted the important contribution from HIS163 in the lateral pocket towards ligand binding and affinity against M^pro^ through computational mutation analyses. Further, we revealed the effects of explicit water molecules and M^pro^ dimerization in the ligand association with the target. Thus, comprehensive molecular-level insights gained from this work can be useful to identify or design potent small molecule inhibitors against SARS-CoV-2 M^pro^.

## Introduction

The novel coronavirus, Severe Acute Respiratory Syndrome Coronavirus 2 (or SARS-CoV-2), was declared as a global pandemic on March 11, 2020^1^ and as of February 5^th^, 2021, the outbreak has caused over 105 million cases and 2.3 million deaths worldwide. While rigorous scientific efforts have resulted in a few clinicially-approved vaccines, the rapid adaptive nature of the virus and its ability to mutate^2–5^ present new challenges. Thus, understanding the specific drug targets of SARS-CoV-2^6, 7^ and clarifying its mechanism of virulence^8,9^ to develop potent therapy^10–14^ or a vaccine^15–17^ against SARS-CoV-2 has since been the main focus of scientific research.

SARS-CoV-2 is an enveloped, positive-sense, single-stranded betacoronavirus that exhibits significant sequence similarity to that of the previous coronaviruses strains such as SARS-CoV-1 (~ 79% sequence identity) and Middle Eastern Respiratory Syndrome Coronavirus (~ 50% similarity^18^). Much like the previously identified coronaviruses, SARS-Cov-2 is composed of structural proteins that are important in producing a complete viral particle, non-structural proteins (nsps) that act as enzymes or transcription/replication factors in the viral life cycle, and numerous accessory proteins^19,20^. Of the various non-structural proteins, the SARS-CoV-2 M^pro^ (interchangeably used with M^pro^ in the text hereafter) remains an attractive therapeutic target as its role of cleaving the coronavirus polyproteins (at 11 sites^14,21,22^) into functional components is vital for viral replication and survival^13,19,22^. Inhibition of M^pro^ can block proteolytic enzyme activity on polyproteins and result in a long polyprotein peptide incapable of performing viral replication by itself^21,23^. In addition, the M^pro^ is highly conserved among coronaviruses. For example, the amino acid sequence of SARS-CoV-2 M^pro^ shares > 96% similarity with that of SARS-CoV M^pro^ (sequence alignment provided in SFig. 1. Therefore, there has been significant attraction towards targeting M^pro^, as seen by the hundreds of crystal structures of M^pro^ (either apo or in complex with ligands) reported in the PDB.

**Figure 1:**
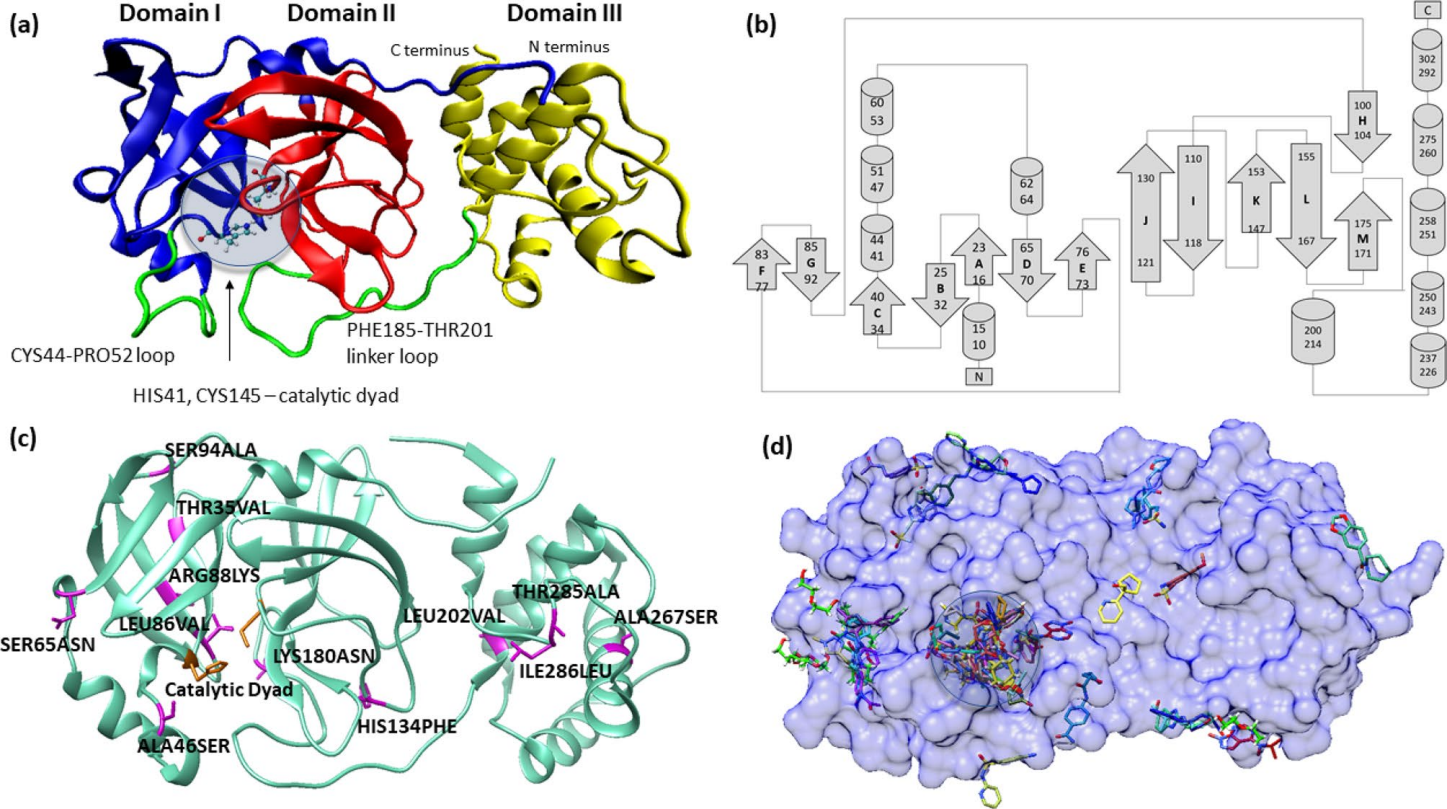
Three dimensional (3D) structure (**a**), and topology (**b**) of SARS CoV-2 M^pro^, along with the variable residues with reference to SARS-CoV-1 M^pro^ (**c**) and the different ligand binding sites described by the known PDB complexes of M^pro^. (**a**) A 3D structure of SARS-CoV-2 M^pro^ is shown with Domains I, II and III colored in blue, red and yellow respectively. Two important loops close to the catalytic dyad (HIS41 and CYS145) are shown in green: CYS44–PRO52 loop flanks the catalytic dyad and PHE185–THR201 connects Domain II with Domain III. (**b**) A topology diagram showing the secondary structural elements in SARS-CoV-2 M^pro^ with β-sheets marked as A-M. (**c**) Structural alignment of SARS-CoV-2 M^pro^ and Sars-CoV-1 M^pro^ shows only 12 mutations of amino acids that are shown as a stick representation in purple. (**d**) The binding of 62 reversible ligands bound at different sites in the SARS-CoV-2 M^pro^ that is shown as a surface representation in blue. The ligands bound within the catalytic site are highlighted with a circle. The molecular graphics in this figure were generated using VMD 1.9.3^63^ (**a**,**c**) and UCSF Chimera 1.14^64^ (**d**) while the secondary structure topology diagram in (**b**) was created using information from the EMBL-EBI online server PDBsum^84,85^.

Based on the known three-dimensional (3D) structure (in Fig. 1a), M^pro^ is composed of three domains including the N-terminal domain I (residues 1–101) domain II (residues 102–184) and the C-terminal domain III (residues 201–301)^24^. As seen in the topology diagram of M^pro^ in Fig. 1b, M^pro^ contains 13 β-sheets and 9 α-helices. Domains I and II are mainly composed of antiparallel β-sheets, with domain I having seven antiparallel β-sheets (labelled A through G) and domain II consisting of the other six antiparallel β-sheets (labelled H through M in Fig. 1b). Unlike these domains, domain III does not have any β-sheet and is composed of a cluster of five α-helices. Domains II and III are linked by a 17-residue long linker loop corresponding to the region PHE185-THR201 (Fig. 1a). The binding site cleft is formed at the interface of domains I and II with HIS41 (from domain I) and CYS145 (from domain II) forming a catalytic dyad^25^, which is unlike a catalytic triad in other cysteine and serine proteases^26,27^. It is proposed that, in SARS-CoV-2 M^pro^, a water molecule at the binding site plays the role of the third residue and involves in the catalytic mechanism of the enzyme^24,28^. A loop formed by CYS44-PRO52 in domain I and the domains II-III linker loop encase the active site as shown in Fig. 1a. Under physiological conditions, M^pro^ exists as a homodimer form, in which two monomers are placed in a perpendicular orientation to each other. In this orientation, the dimer interface is formed by the domain II of the first monomer, particularly through residue GLU166 and the N-terminal residues of the second monomer (generally called the “N-finger”)^13^, enclosing a contact surface area of ~ 1390 Å^221^. The N-finger from the monomer B is critical as it closes the catalytic site in monomer A. Further, it is proposed that domain II is crucial in modulating the formation of dimer interface in M^pro^^29^. Comparison of the structure of SARS-CoV-2 M^pro^ against the SARS-CoV-1 M^pro^, showed amino acid variations in only 12 positions (in Fig. 1c), which include THR35VAL, ALA46SER, SER65ASN, LEU86VAL, ARG88LYS, SER94ALA, HIS134PHE, LYS180ASN, LEU202VAL, ALA267SER, THR285ALA, ILE286LEU. Except ALA46SER that is part of the CYS44-PRO52 loop in domain I, all the other mutations are away from the catalytic site and mostly on the surface regions of the three domains.

Given the important roles of M^pro^ in the coronavirus infection, there have been numbeous efforts using computational methods^18,28–39^ and experimental approaches^21,40–42^ to find potent inhibitors of M^pro^. For example, a screening performed using the Fluorescence Resonance Energy Transfer-based enzymatic assay^41^ identified a number of inhibitors such as boceprevir, calpain inhibitors II and XIII, and GC-376 that inhibited M^pro^ with low micromolar IC50 values under the experimental conditions. In another study, Douangamath et al.^14^ performed rigorous fragment screening against SARS-CoV-2 M^pro^ and reported several crystal structures of covalent and non-covalent fragments in complex with M^pro^. Most of the fragments screened were found to be bound within the catalytic site of M^pro^; however, some non-covalent fragments were bound at different other sites, including the dimer interface site. These fragment-bound M^pro^ crystal structures^14^ provide a wealth of information about the ligand–M^pro^ interactions and also reveal several other cavities on the surface of M^pro^. While a large number of experimentally resolved structures of ligand M^pro^ complexes are emerging rapidly, it might be important and useful to understand the dynamic interactions of these complexes and to garner any novel molecular level insights from the dynamic process. It is known that molecular dynamics (MD) simulation is a powerful tool to study the dynamic molecular recognition processes in biological systems such as ligand–protein and protein–protein complexes^43–47^. Earlier, MD has been successful in the discovery of a novel cryptic binding trench in HIV integrase enzyme^48^ that eventually led to the development of novel anti-HIV inhibitors such as raltegravir. Recently, MD methods have been extensively used to understand the structures and ligand interactions of SARS-CoV-2 targets including RdRp^30,49–51^, M^pro^^18,28,35,38,39,52–54^, and S protein^32^, and computational design of peptide-based subunit vaccines^31^. Therefore, in this work, we use MD to characterize the dynamic interactions of almost all the crystal structures released as of June 10th, 2020 in PDB (the list is provided in the supplementary Table, ST1). This includes a total of 62 ligand-bound complexes of M^pro^, whose 3D superposed structure is shown in Fig. 1d. and the chemical structures of all the ligands are shown in supplementary Table, ST1. Using the MD trajectories of the systems, we calculated the binding free energy of the ligand-bound complexes using the end-point molecular mechanics generalized Born surface area (MM-GBSA) approach and identified the key residues that stabilize the ligand–M^pro^ interactions. Our results highlight the significance of a lateral pocket in the ligand–M^pro^ affinity and, in particular, the key role of HIS163 in this pocket through computational mutagenesis. Thus, our work should be useful to further our knowledge about the interactions of reversible ligand–SARS-CoV-2 M^pro^ complexes at the molecular level.

## Computational methods

### Preparation of ligand–M^pro^ complexes for MD

A total of 62 experimentally resolved crystal structures of reversible small molecule-SARS-CoV-2 M^pro^ complexes (the list of PDB codes are provided in supplementary table ST1), along with the crystal structure of an apo M^pro^ (PDB: 6M2Q), were retrieved from PDB. Initially, all the DMS compounds, water molecules, and ions were removed from the downloaded structures. For each complex, the AMBERff14SB force field^55^ and the general AMBER force field (GAFF2)^56^ were used for the protein and ligand, respectively. The partial charges of the ligand were assigned using the semi-empirical AM1-BCC^57^ procedures in antechamber^58^. The missing ligand parameters were obtained using the parmchk2 tools available within the AmberTools^59^. Subsequently, the ligand–M^pro^ complexes were prepared using tleap program in AMBER18 package^60^ by solvating the complex in a cubic box of TIP3P water molecules with a minimum of 12 Å distance between the box boundaries and the closest solute atoms. The solvated systems were subsequently charge neutralized with 150 mM concentration of NaCl counter ions. Thus, the systems were prepared for subsequent MD simulation.

### MD simulation of ligand–M^pro^ complexes

All MD simulations were performed using the AMBER18 molecular dynamics program^60^ with pmemd.cuda engine. MD simulation of each ligand–M^pro^ complex was performed in five consecutive stages: (1) energy minimization, (2) NVT heating, (3) NPT equilibration, (4) NPT pre-production simulation, and (5) production simulation. The initial stage of gradient minimization of the system was performed in six rounds. In the first round of minimization, a strong harmonic constraint of 100 kcal mol^−1^ Å^−2^ was applied on the solute atoms and 10,000 steps of minimization (1000 steps of steepest descent minimization + 9000 steps of conjugate gradient minimization) was performed. The next four rounds of minimization were performed with almost the same parameters as the first round with the exception of the harmonic constraints that were reduced as 50 > 10 > 5 kcal mol^−1^ Å^−2^ in each round. Finally, 20,000 steps of energy minimization of the system without any constraints was performed. Following the minimization, each complex was gradually heated to 310 K over a duration of 100 ps and, subsequently, equilibrated in four rounds, each being 400 ps long. In the first two rounds of equilibration, positional restraints were applied on the non-hydrogen atoms of the protein residues using restraint force constants of 5 kcal mol^−1^ Å^−2^ (first round) and 0.1 kcal mol^−1^ Å^−2^ (second round). Following this, the third round of equilibration was performed with a weak restraint of 0.01 kcal mol^−1^ Å^−2^ applied to only the backbone atoms of protein residues. Finally, a 400 ps long restraint-free equilibration of the system was carried before proceeding to production simulation. The equilibrated system underwent a 2 ns long pre-production simulation under isothermal-isobaric (NPT) conditions. Following the pre-production run, each complex was subjected to 3 subsequent 10 ns long MD simulations (making a total of 30 ns long MD simulations) using an integration time step of 2 fs. Throughout the simulation, the temperature was controlled using the Langevin thermostat^61^ and the pressure was kept at 1 bar using the Berendsen barostat^62^. During the production runs, coordinates were stored every 2 ps thereby resulting in a total of 15,000 snapshots from a 30 ns long MD trajectory. VMD 1.9.3^63^ and USCF Chimera^64^ were used to visualize the molecular dynamics trajectories. All simulations were performed using the Graham and Cedar GPU clusters available within the ComputeCanada infrastructure. The resulting MD trajectories of the ligand–M^pro^ complexes were processed through the CPPTRAJ tool^65^ to generate the root mean square deviation (RMSD) plots and the per-residue root mean square fluctuation or RMSF (for the apo trajectories only).

### Relative binding free energies of ligand–M^pro^ complexes

Following the MD simulation, for each complex, the last 10 ns of the MD trajectory was used to compute the binding free energy scores between the ligand and the M^pro^ bound in a complex. For this purpose, we employed the MM-GBSA method^66^ with the implicit solvent model of GB-Neck2 (or igb = 8)^67^. The snapshots sampled at a regular interval of 10 ps and thus a total of 1000 frames were used to calculate the MM-GBSA energies. The binding free energy (ΔG_bind_) using the MM-PB(GB)SA can be estimated as,

1 $$\Delta G_{bind} = \Delta E_{MM} + \Delta G_{Solv} - T\Delta S$$

Here, ΔE_MM_ is the summation of non-bonded and bonded interaction energies^66^. The solvation energy, Δ*G*_*solv*,_ is the sum of the polar and non-polar contributions of solvation, where the polar solvation terms are calculated using a Generalized-Born model or a Poisson–Boltzmann solver and the non-polar terms are computed based on the size of the solvent-accessible surface area in the ligand and M^pro^^66^. The last term corresponding to conformational entropy (TΔS) is computationally expensive and, therefore, is neglected. Previous studies^68,69^ have shown that incorporating the entropic contribution in the ΔG_bind_ calculations do not assure the accuracy of the calculated binding free energy. Therefore, in this work, we calculate only the relative binding free energy, which is often considered useful in relative ranking of compounds. All MM-GBSA calculations in this study were carried out using the MMPBSA.py script^70^ included in the AmberTools^60^. The pairwise decomposition analyses (idecomp = 4) using the MD trajectories were also carried out to identify the key ligand–residue energetic contributors to the binding free energy of the complexes^71^.

### Relative binding free energies of ligand–M^pro^ complexes in the presence of explicit water molecules

The binding free energies of ligand–M^pro^ complexes were also calculated in the presence of 0–6 explicit water molecules. These calculations were performed using an MM-GBSA variant called the NWAT-MMGBSA. Maffucci et al.^72^ demonstrated that this NWAT-MM-GBSA method can improve the correlation between the calculated and measured binding free energies of biological systems. All parameters in the NWAT-MM-GBSA calculations were the same as those explained for the routine MM-GBSA method (“Relative binding free energies of ligand–M^pro^ complexes”), except the explicit water molecules that were captured from the MD trajectories using the CPPTRAJ tool^65^ of Amber 18 program^60^. The cpptraj module was employed to select the desired number of snapshots and to generate the related topology files for the free energy calculations; whereas, the interface residues were found using the “InterfaceResidues” Pymol script. The NWAT calculation for each system was repeated by increasing the number of explicit water molecules from 0 to 6. All NWAT calculations utilized the shell scripts from Maffucci et al.^72,73^.

### Principal component analyses

All the structure dynamics calculations and Principal component analyses (PCA) were performed in the Protein Dynamics (ProDy) package release 1.11^74^ using Python 3.7.0 (Python Software Foundation, Delaware, USA). The Normal Mode Wizard (NMWiz) release 1.2 was plugged into the Visual Molecular Dynamics (VMD) Viewer, release 1.9.2^63^. For the essential dynamic analysis (EDA) of the full-length protein, only *N*_*t*_ = 296 C_α_-atoms (Lys5–Cys300) were considered; omitting 4 N-terminal and 6 C-terminal residues because of the high mobility of these regions. For the EDA of the domains I and II, *N*_*t*_ = 191 C_α_-atoms (Lys5–Gly195) were considered. In both cases, the superposition was based on a set of *N* < *N*_*t*_ atoms belonging to secondary structure (β-strands for D_I/II_; β-strands and α-helices for the full length protein) in order to maximize the relative mobility of loops and linker segments. Details on the theoretical and practical aspects for PCA/EDA methods can be found elsewhere (for reference check^75,76^).

For the optimized C_α_ superposition of an ensemble configurations of a set of *N* atoms, ProDy implements an iterative superposition (“*iterpose”*) that gives a unique solution that minimizes the average RMSD of the ensemble. Briefly, the *i*th member of the ensemble, i.e. the *i*th conformation, is represented by a *3N*-dimensional column vector defined as

2 $${\varvec{R}}_{i} = \left[ {{\varvec{r}}_{1,i} {\varvec{r}}_{2,i} {\varvec{r}}_{3,i} \ldots {\varvec{r}}_{N,i} } \right]^{{\text{T}}}$$

with ${\varvec{r}}_{p,i} = [x_{p} y_{p} z_{p} ]_{i}$ being the *3D*-position vector of the *p*th atom in the *i*th configuration. For an ensemble of M configurations, the average *3 N*-dimensional column vector is defined as

3 $$\overline{\user2{R}} = \frac{1}{M}\mathop \sum \limits_{i = 1}^{M} {\varvec{R}}_{i} = \left[ {\overline{{{\varvec{r}}_{1} }} \overline{{{\varvec{r}}_{2} }} \overline{{{\varvec{r}}_{3} }} \ldots \overline{{{\varvec{r}}_{N} }} } \right]^{{\text{T}}}$$

The mean square fluctuation (MSqF) and RMSF for the *p*th position are calculated over all the *M* conformations by

4 $$MSqF_{p} = \left\langle {\left\| {{\Delta }{\varvec{r}}_{p} } \right\|^{2} } \right\rangle = \frac{1}{M}\mathop \sum \limits_{i = 1}^{M} \left\| {{\varvec{r}}_{p,i} - \overline{{{\varvec{r}}_{p} }}^{2} } \right\|$$

5 $$RMSF_{p} = \left\langle {\left\| {{\Delta }{\varvec{r}}_{p} } \right\|^{2} } \right\rangle^{1/2} = \sqrt {\frac{1}{M}\mathop \sum \limits_{i = 1}^{M} \left\| {{\varvec{r}}_{p,i} - \overline{{{\varvec{r}}_{p} }} } \right\|^{2} }$$

The root means square deviation (RMSD) of the *i*th conformation is calculated over all the *N* atoms by

6 $$RMSD_{i} = \left\langle {\left\| {{\Delta }{\varvec{r}}_{i} } \right\|^{2} } \right\rangle^{1/2} = \sqrt {\frac{1}{N}\mathop \sum \limits_{p = 1}^{N} \left\| {{\varvec{r}}_{p,i} - \overline{{{\varvec{r}}_{p} }} } \right\|^{2} }$$

The average RMSD for the ensemble is calculated by

7 $$\overline{RMSD} = \left\langle {RMSD_{i} } \right\rangle = \frac{1}{M}\mathop \sum \limits_{i = 1}^{M} RMSD_{i}$$

The RMSD between two *i* and *j* conformations is calculated by

8 $$RMSD_{ij} = \left\langle {{\Delta }{\varvec{r}}_{ij}^{2} } \right\rangle^{1/2} = \sqrt {\frac{1}{N}\mathop \sum \limits_{p = 1}^{N} \left\| {{\varvec{r}}_{p,i} - {\varvec{r}}_{p,j} } \right\|^{2} }$$

The interpose routine proceeded as follows: (i) a random configuration of the ensemble was selected, and its coordinates were taken as an *average* conformation of the ensemble; (ii) each member of the ensemble was superimposed onto the average conformation by a rigid-body translation and rotation to minimize the RMSD of the configuration (Eq. 6); (iii) a new average conformation was calculated for the ensemble by using Eq. (3). Steps (ii) and (iii) were iteratively performed until the RMSD between two successive average configurations (Eqs. 7,8) were lower than an arbitrary threshold (usually 0.001 Å).

The subspace overlap between *n* EDA modes of ensemble A and *o* EDA modes of ensemble B, can be obtained from the root mean square inner product (RMSIP), defined by

9 $$RMSIP = \sqrt {\frac{1}{n}\mathop \sum \limits_{k = 1}^{n} \frac{1}{o} \mathop \sum \limits_{s = 1}^{o} \left( {{\varvec{v}}_{k} \cdot {\varvec{u}}_{s} } \right)^{2} }$$

### Preparation of mutated systems

Each of the selected ligand–M^pro^ complexes was modified by a single point mutation of HIS163 to ALA163 using the ‘swapaa’ command in UCSF Chimera^64^. The mutated systems were then prepared and subjected to 30 ns long MD simulation as discussed in the above sections for the wild-type (WT) complexes (in “Preparation of ligand–M^pro^ complexes for MD” and “MD simulation of ligand–M^pro^ complexes”). However, for the mutated systems, the relative binding free energies were calculated only using the MM-GBSA approach (“Relative binding free energies of ligand–M^pro^ complexes”) and the free energies in the presence of explicit water molecules were not computed.

### Preparation of ligand-bound dimer models of M^pro^

The models of ligand-bound M^pro^ dimer complexes were constructed using the experimentally resolved 3D structure of a M^pro^ dimer in the PDB (PDB: 6WTT). This was performed by aligning the ligand-bound M^pro^ monomer on the structure of M^pro^ dimer and by deleting the duplicate chain after alignment. All proceduces in building the dimer models were performed using the UCSF Chimera. Subsequently, all the dimer complexes were subjected to 30 ns long MD simulation and followed by binding free energy estimation using the MM-GBSA method as described above.

## Results and discussion

### MD simulation and analyses of apo SARS-CoV-2 M^pro^

**Figure 2:**
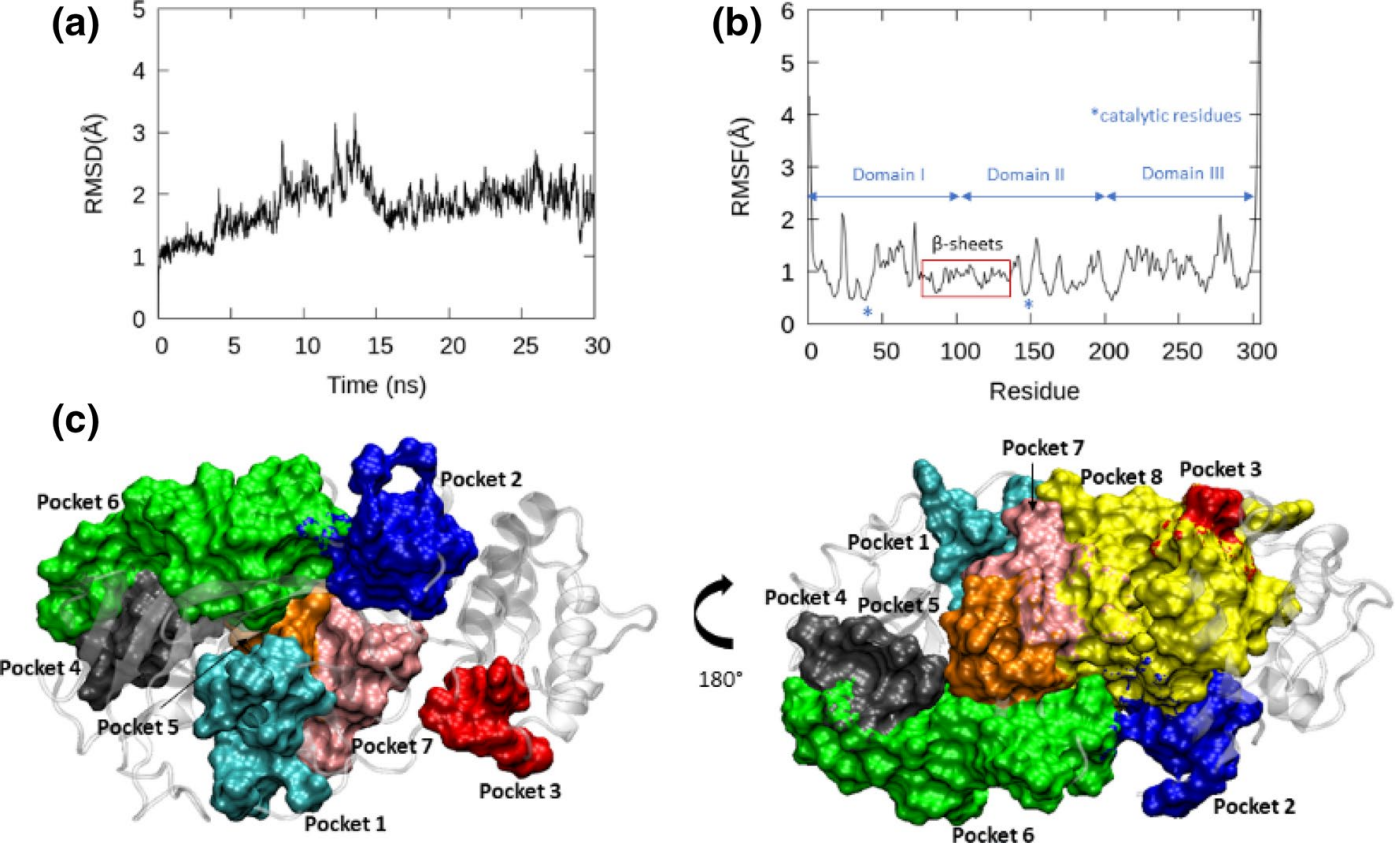
The backbone RMSD (**a**) and RMSF (**b**), of apo M^pro^ (PDB: 6M2Q) and the different pockets on the M^pro^ (**c**,**d**) identified through MD simulation. RMSD of the apo structure changed between 1–3 Å during MD. The per-residue fluctuation during MD is shown as RMSF with the different domains in the enzyme labelled to reflect this. Using the “MDpocket” tool, various transient pockets (named as Pocket 1–8) were identified and characterized within the apo structure during the MD simulation. The graphs in this figure (**a**,**b**) were generated using GNUplot (v5.2 patchlevel 8; http://www.gnuplot.info/) while the molecular graphics were generated using VMD 1.9.3^63^ (**c**,**d**).

To understand the dynamics of a SARS-CoV-2 M^pro^ in the absence of a bound ligand, we performed a 30 ns long MD simulation of an apo structure of M^pro^ downloaded from PDB (PDB ID: 6M2Q). Initially, the stability of the structure was assessed by plotting the fluctuation of backbone RMSD (Fig. 2a) during MD simulation. It was noted that the backbone RMSDs of M^pro^, as a whole, exhibited a fluctuation in the range of 1–3 Å, most often only varying within 0.5–1 Å. As can be seen in Fig. 2a, the RMSD plot showed a slightly increased fluctuation, reaching ~ 3 Å, around 14–15 ns timescale that was caused due to the movements of flexible C- and N-terminal loops. We analyzed the backbone RMSDs of the individual domains such as domain I–III in M^pro^ (see in Supplementary Figure, SFig. 2) to deduce the dynamic segments. We found that domains I and II remained quite stable throughout the simulation with backbone RMSD changes within ~ 0.7 and 1.2 Å. Nevertheless, Domain III, featuring a globular cluster of five helices exhibited, fairly higher fluctuations that reached > 1.5 Å during the mid-course of simulation and reaching a plateau during the last 10 ns. We performed RMSF analyses (Fig. 2b) to reveal the averaged per-residue fluctuations during MD simulations. The most stable segments of M^pro^ include the β-sheets in domains I and II, the loop connecting these domains (ASP92-PHE112), and the helices of domain III. Unsurprisingly, the most flexible regions occurred at the N and C terminus of the protein^47^. In addition to this segment, the other regions that displayed elasticity during MD were the loops connecting different secondary structures in domain III (ASN277-THR292), domain II (ASP153-ASP155 and LEU167-VAL171), and domain I (THR45-ASN65 and ALA70-VAL73). The loops enclosing the catalytic site in M^pro^ such as CYS44-PRO52 loop and the PHE185-THR201 linker loop (domain II-III linker) only exhibited small variations indicating their role in stabilizing the active site. The catalytic residues, HIS41 (from domain I) and CYS145 (from domain II) remained highly stable throughout the simulation. Despite the small flexibility within the different loop regions, the apo protein structure proved to be quite stable and only reached a maximum RMSD of 3 Å and a maximum RMSF of 2 Å. We also assessed different electrostatic interactions in M^pro^ that contributed to the stability of the structure during MD (Supplementary figure SFig. 3). We found strong hydrogen bonds (H-bonds) between the carboxylate side chain in THR111 and the hydroxyl moiety in ASP295 in domain II of M^pro^ (plot shown in the supplementary figure, SFig. 4a). In addition, we found stable salt bridge interactions formed by two ASP-ARG residue pairs: one pair of salt-bridge was formed between ARG131 in domain II and ASP289 in domain III (supplementary figure, SFig. 4b); and another pair of salt-bridge was established between ARG40 in domain I and ASP187 located in the DII-DIII linker loop (supplementary figure, SFig. 4c). These observations are consistent with the previous studies^77,78^. In addition, we also identified a critical water-mediated interaction near the active site of M^pro^, where a central water-molecule formed a 3-way H-bond network with residues HIS41 (the catalytic residue), ASP187, and HIS164. We noticed that whenever the water molecule at this tri-junction moved out of the binding site during MD, another water molecule entered the site and maintained this H-bond network. This suggests the critical role of water molecules in the stability of the binding site during MD simulation. Moreover, as indicated earlier, it is hypothesized that this water molecule in the binding site could play a role of the third residue in regulating the M^pro^ catalysis process^24,28^.

Since the 62 crystal structure complexes studied in this work show ligand binding at different sites other than the catalytic site in M^pro^, we explored the dynamics of different pockets in M^pro^. A protein pocket detection tool, MDpocket^79^, was employed for this purpose. Using the 30 ns MD trajectory of the apo M^pro^, we sampled snapshots at a regular interval of 10 ps from the last 25 ns, thereby, using 2500 snapshots of the enzyme for the pocket analyses. Each snapshot was saved as an individual PDB file and was used to determine favorable binding pockets within the protein. In addition to the main binding site, many small and transient pockets were identified using the putative channels and small cavities search options within MDpocket^79^. Through this analysis, we identified a total of 8 pockets in M^pro^ monomer (Fig. 2c,d) that we named Pocket1–Pocket8 in the text hereafter. A table comparing different characteristics of each of these pockets such as residues, pocket volume, and PDB codes of the structures with ligand bound in the respective pockets are presented in the supplementary table ST2. The parameters used for MDpocket analyses are also provided in the footnote of this supplementary table. Pocket 1, the catalytic pocket, was encircled by the loop composed of PHE140-CYS145, a β-sheet (‘L’ in Fig. 1b) made of HIS163-GLU166, and the residues from the domain II-III linker loop including HIS172, VAL186, ASP187, ARG188, GLN189, and GLN192. The volume of this pocket was ~ 800 Å^3^ in our MD snapshots and, as expected, most compounds in this study were bound in this pocket. Among the other pockets, three pockets (including pockets 2, 3, 7) were found within the domains II and III, one pocket (pocket 4) was found in domain I, and two pockets (pocket 5, 6) were found between domains I and II (Fig. 2c,d). Pocket 2 was a small cavity (volume < 320 Å^3^) that was found between domains II and III and formed by the N-terminal (PHE3–PHE8) and C-terminal (PHE291–THR304) residues, along with residues THR111 and GLN127 from domain II. In two of the crystal structures studied here, 5RFA (1-methyl-*N*-{[(2S)-oxolan-2-yl]methyl}-1H-pyrazole-3-carboxamide) and 5RGQ (1-(4-fluoro-2-methylphenyl)methanesulfonamide), ligands were found to bind at this pocket. Pocket 3 was a small and transient surface pocket formed by THR198 (from domain II-III linker) and the selected residues from domain III (MET235 ASN238 TYR239 GLU240 PRO24). Fragments such as 2-{[(1H-benzimidazol-2-yl)amino]methyl}phenol (PDB: 5REC), [(2 ~ {R})-4-(phenylmethyl)morpholin-2-yl]methanol (PDBL: 5RGS), and (2R,3R)-1-benzyl-2-methylpiperidin-3-ol (PDB: 5REE) were found to bind in this pocket. Pocket 7 was another small pocket that was mainly formed by residues from domain II (ARG105-PRO108, MET130, PHE134, PHE181-PRO184) but was located near the interface between domains II and III. It may be due to the transient nature of this pocket that only one carboxamide containing ligand (PDB: 5REG) was found to bind in this pocket. Pocket 4, a fairly large pocket of 600 Å^3^, was found to be formed majorly by the β-sheets composed of residues ASP34-TYR37, GLY79, HIS80, SER81, LYS88-LYS90. This pocket remained stable throughout MD simulation, which suggests that this could be a viable pocket for ligand binding. Consistent with this observation, we found that ligands (of different sizes and classes) from at least 7 known crystal structures (5RFC, 5RH4, 5RE6, 5RE5, 5RGG, 5RFB, and 6YVF) were found to be bound in this pocket. Pocket 5 was slightly smaller than Pocket 4 but remained quite stable throughout the MD trajectory. This pocket was positioned near the catalytic site (or Pocket 1) between domains I and II, but on the opposite face of the protein. Four ligands were found to interact with this pocket in the crystal structures (PDBs: 5REI, 5RED, 5RF5, 5RGR). There was another large pocket of 800 Å^3^, Pocket 6, that was also found between domains I and II, and five ligands from the PDB structures of 5RF4, 5RFD, 5RE8, 5RF9, and 5RFJ were found to be interacting at this site. In addition to the well-defined pockets discussed above, we found a huge surface area (dubbed here as pocket 8) of 2250 Å^3^ that was found largely on the surface of domains 2 and 3 (Fig. 2c) and connecting many of the smaller cavities. There was only one crystal structure (PDB: 5RF0) that described the binding of a ligand within this surface. Mapping these residues onto a dimer structure of M^pro^ (6WTT), we found that this surface was mostly buried at the dimer interface (refer to the figure provided in the footnote of supplementary table, ST2), which explained the existence of this dynamic surface area during MD analyses. However, since the dimerization in SARS-CoV-2 M^pro^ is crucial for the activity of the enzyme and thus the replication of the virus, this large surface area of pocket 8 can be an interesting site for drug-binding, as targeting dimerization in M^pro^ is considered as a potential therapeutic strategy to inhibit the virus replication^13^.

### Stability of ligand–SARS-CoV-2 M^pro^ complexes during MD simulation

Following the analyses of pockets and their dynamics in the apo structure of M^pro^, we performed MD simulation and analyses of the selected crystal structures (supplementary table, ST1) in this study. As described in the methods section, we initially performed 30 ns long MD simulation of each of the 62 reversible ligand–M^pro^ complexes that resulted in a total of 1.86 μs long molecular trajectories for analyses. We analyzed the stability of the protein and ligand structures using the RMSD analyses. To simplify the data presentation, we calculated the average backbone RMSD fluctuations of the proteins and the average RMSD fluctuation of the ligand in each complex during MD simulation, (provided in the supplementary table, ST3). As can be seen in SFig. 5a, the average backbone RMSD variation of proteins in all the complexes were mostly in the range of 1.5–2.3 Å, when compared to that of 1.81 Å for the apo (or ligand-free) M^pro^ structure. This suggests that the binding of the ligand had only a limited impact on the structural dynamics of the enzyme. Two PDB structures, 5RE5 and 5REG, were the only exception to this trend and displayed slightly higher average backbone RMSD value of 2.84 Å and 2.87 Å, respectively, which are still within the fluctuations that are usually seen for proteins of this size, as also previously shown by an MD study^77^ of SARS-CoV-2 M^pro^ structure. Analyses of their trajectories showed that the PHE185-THR201 linker loop was slightly more flexible in these complexes during a simulation. The flexible nature of this long loop connecting domains II and III is already known^77,80^.

Nevertheless, unlike the stable proteins, ligands that were co-crystallized in the structures studied here displayed a spectrum of behaviour that described their nature of stability and interactions with the target. We initially analyzed the stability of the ligands bound within the HIS41-CYS145 catalytic binding site (or pocket 1), refer to SFig. 5b. It was noted that most of the ligands (excluding PDB complexes of 5R7Z, 5REH, 5RF2, 5REE, and 5RF3) bound within the catalytic binding site exhibited an average RMSD in the range of 1–8 Å (SFig. 5c). Ligands with lower RMSD values (i.e., < 3 Å) indicated that they remained more stable in the initial pose (e.g., ligands in PDB structures: 5RG1, 5RGW, 6W63, 5RH3); whereas, an average RMSD in the range of 3–8 Å indicated that the ligand, despite bound stable in the catalytic site, underwent some conformational changes to stabilize the association with M^pro^ (e.g., ligands in the PDB structures 5R7Y, 5R82, 5REZ, 5RH8, and 5RHD). For instance, in the 5RHD structure (in supplementary figure, SFig. 6), the 1-[4-(methylsulfonyl)phenyl]piperazine ligand was initially bound in a pose in which the methylsulfonyl group was interacting with the “L” β-sheet residues such as MET165-LEU167 and the piperazine moiety was stabilized by the H-bond interactions with CYS44-PRO52 loop that flanked the catalytic site. During MD simulation (in the first 10 ns of the simulation), the methylsulfonyl group changed its orientation and interacted with the loop containing GLY143, SER144 and CYS145. Thus, this change of binding pose of the ligand during MD simulation resulted in a slightly higher RMSD range of over 4.5 Å. However, there were a few ligands that bound within the catalytic site and had a weak association with M^pro^ that resulted in their unbinding. For instance, the smallest ligands in this study such as 1-azanylpropylideneazanium (in 5RF2) and pyrimidin-5-amine (in 5RF3) that were originally bound within the binding site displayed weak interactions with M^pro^ and egressed during MD simulation. This explained the large average RMSDs for these ligands (in SFig. 5c) during our MD simulations. Another ligand (~ {N}[2-(5-fluoranyl-1 ~ {H}-indol-3-yl)ethyl]ethanamide) that was co-crystallized in PDB 5R7Z was initially bound in a pose in which the fluroindole group was buried into the catalytic site and the ethanamide group was projecting towards the surface of M^pro^. However, during 30 ns long MD simulation, this ligand explored different orientations within the catalytic site and eventually unbound from the pocket. Similar behaviour was also seen in 1-cyclohexyl-3-(2-pyridin-4-ylethyl)urea bound with M^pro^ in 5REH structure, where the pyridine ring was bound inside the pocket and the cyclohexane moiety stuck outside on the surface that resulted in weak interactions with M^pro^ and egression during MD. It should be noted that the current simulation of ligand-bound complexes was based on the monomer state of M^pro^. However, it is known that M^pro^ exists as a dimer, where the N-terminus of the second chain will close the binding site of the first monomer chain. Therefore, it is possible that 5REH could be a more stable ligand under a dimer conformation. To address this concern, we analyzed the dynamic interactions of the select ligands with the dimer state of M^pro^. These include the ligands that were originally bound within the catalytic site of M^pro^ and unbound during MD. The results of these dimer simulations are discussed in a later section.

Next, we analyzed the stability of complexes in which the ligands were bound in the other pockets (pockets 2–8) found in the apo structure. The average RMSD of the ligands bound in these pockets are presented in the supplementary figure, SFig. 7. Two ligands, 1-(4-fluoro-2-methylphenyl)methanesulfonamide (5RGQ) and 1-methyl-*N*-{[(2S)-oxolan-2-yl]methyl}-1H-pyrazole-3-carboxamide (5RFA) were bound in pocket 2, which was near the dimer interface. While the former ligand remained stable throughout the 30 ns long MD (average RMSD of 2.29 Å), the latter disassociated during simulation and displayed higher RMSD changes. Despite pocket 3 being a relatively small cavity, the ligands 2-{[(1H-benzimidazol-2-yl)amino]methyl}phenol (5REC) and [(2 ~ {R})-4-(phenylmethyl)morpholin-2-yl]methanol (5RGS) remained highly stable highlighting their complementing properties to this pocket through H-bond interactions with TYR239 and GLU240 (more discussion are presented in the following section). Nevertheless, the piperidinol based ligand (in 5REE) was not able to make such key H-bonds therefore unable to maintain stable interactions with pocket 3, which was reflected by its average RMSD of 12.47 Å during MD. From the RMSD analyses, it was clear that pocket 4 favoured an acid group or its derivatives when compared to an amide or amine-based ligands. For example, the ligands based on benzoic acid (6YVF), propanoic acid (5RH4), and carbamate (5RFC) exhibited stable interactions with this pocket. In comparison, the carboxamide-based (5RGG and 5RE5), acetamide-based (5RE6) and ethanamine (5RFB) ligands remained loosely bound to this pocket. It was interesting to note that all the ligands that bound to pockets 5 and 6 that were located between domains I and II displayed weak affinity towards this site, as all of them unbound during the MD simulations. This suggests that this site is either not druggable or the ligands explored in this screening do not have the physicochemical features to complement this site. Thus, our stability analyses highlight the stability and dynamic interactions of the experimentally reported ligand–M^pro^ complexes.

### Binding free energy analyses of ligand–M^pro^ complexes

**Figure 3:**
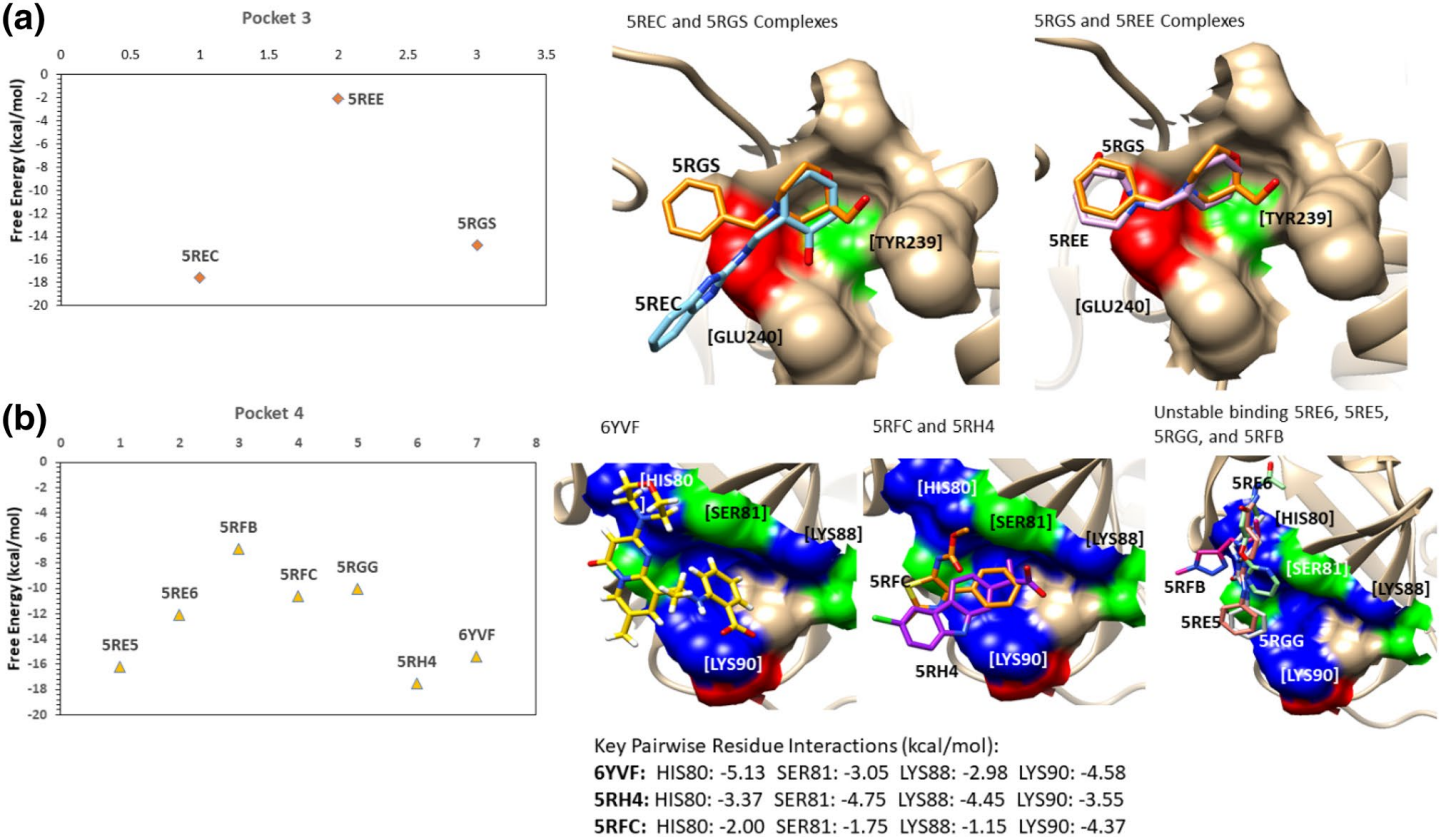
The binding free energies and the binding poses of the ligand–M^pro^ complexes in which the ligands were bound in pocket 3 (**a**) and in pocket 4 (**b**). Both in pocket 3 and pocket 4, a few ligands remained stable due to their ability to make key electrostatic interactions with the residues in the pocket, while the other ligands failed to make such interactions that resulted in their weak affinity against M^pro^. The molecular graphics in this figure were generated using UCSF Chimera 1.14^64^ (**a**,**b**) while the plots were generated using Microsoft Excel 365 (https://www.office.com/).

In the earlier section, we discussed the stability of reversible ligands in different pockets in SARS-CoV-2 M^pro^ structure. Building on these analyses, we calculated the binding free energy of the ligand–M^pro^ complexes using the end-point MM-GBSA method based on the snapshots of the complexes sampled from their respective MD trajectories. For each complex, we sampled the snapshots at a regular interval of 10 ps from the last 10 ns trajectory, which resulted in 1000 frames for the MM-GBSA calculations. The binding free energies of the complexes are provided in the supplementary table, ST3. We also decomposed the ligand–residue pairwise energetic contribution to the binding free energy of the complex. We intend to evaluate the binding affinity of the ligand–M^pro^ complexes that were relaxed through MD simulation and identify the key residues contributing to their affinity. As expected, the complexes in which the bound-ligand disassociated in the course of MD (as discussed in the earlier section) displayed a low affinity of > − 10 kcal/mol that described weak and non-specific interactions with the surface of M^pro^ during the simulation (supplementary figure, SFig. 8). This was particularly true for the ligands bound in pockets 5 and 6 located at the junction of domain I and II, as these complexes did not show any stable interactions. In a similar nature, those ligands that egressed from the catalytic pocket also had weak affinity against M^pro^ and these include the structural complexes of 5REH, 5RF2, 5RF3, 5RF8 and 5R7Z as discussed in the stability analyses. Figure 3 compares the binding free energies of ligands bound to pocket 3 (Fig. 3a) and pocket 4 (Fig. 3b). In pocket 3, the ligands, 2-{[(1H-benzimidazol-2-yl)amino]methyl}phenol (5REC) and [(2 ~ {R})-4-(phenylmethyl)morpholin-2-yl]methanol (5RGS), displayed binding affinity scores of − 17.56 kcal/mol and − 14.79 kcal/mol, respectively. Analysing their binding poses (in Fig. 3a), the alcohol (–OH) functional group in these compounds bound inside the cavity and was stabilized by the H-bond interactions with TYR239, GLU240 and MET235. Nevertheless, in the binding pose of the ligand ((2R,3R)-1-benzyl-2-methylpiperidin-3-ol) within pocket 3 in M^pro^ (5REE complex), the phenyl ring was occupying the cavity and the OH group in the piperidin-3-ol moiety was not able to make H-bond interactions with the key residues in this pocket. Consequently, the ligand moved out of pocket 3 during MD, thereby resulting in a poor affinity of ~ − 2 kcal/mol (in Fig. 3a). Earlier we noted that pocket 4 favoured the binding of an acid group as opposed to the amide or amine-based fragments. Consistent with this interpretation, the binding affinity scores of the propanoic acid ligand (5RH4) and benzoic acid ligand (6YVF) were − 17.50 kcal/mol, and − 15.35 kcal/mol, respectively (Fig. 3b). Analyses of their binding poses showed that acid groups in these ligands burried themselves in the cavity. In this orientation, they made electrostatic interactions (H-bonds) with the LYS residues in the pocket (LYS88 and LYS90). Energy decomposition analyses revealed that the pairwise interactions of the ligand with the key restudies such as HIS80, SER81, LYS88 and LYS90 made significant contributions to the binding affinity of the complexes (Fig. 3b).

Nevertheless, in the binding pose of a carbamate-based ligand in the 5RFC structure (Fig. 3b), the hydrophobic ring of the ligand was bound deep inside the pocket, and its carboxy group was placed on the edge of the pocket and made some electrostatic contacts with SER81 and LYS90. This pose was not favoured and as a result, this ligand displayed weaker affinity (− 10.63 kcal/mol) when compared to the other acid-based ligands. Therefore, the amide or amine compounds including carboxamide-based (5RGG and 5RE5), acetamide-based (5RE6) and ethanamine (5RFB) ligands were not able to accommodate the cavity in pocket 4, and as a result, they were not stable in the pocket and made several transient interactions. The binding free energy scores in Fig. 3b do not represent the ligand affinity to one site rather a cumulative effect of interacting with different sites on the surface of M^pro^ during MD.

**Figure 4:**
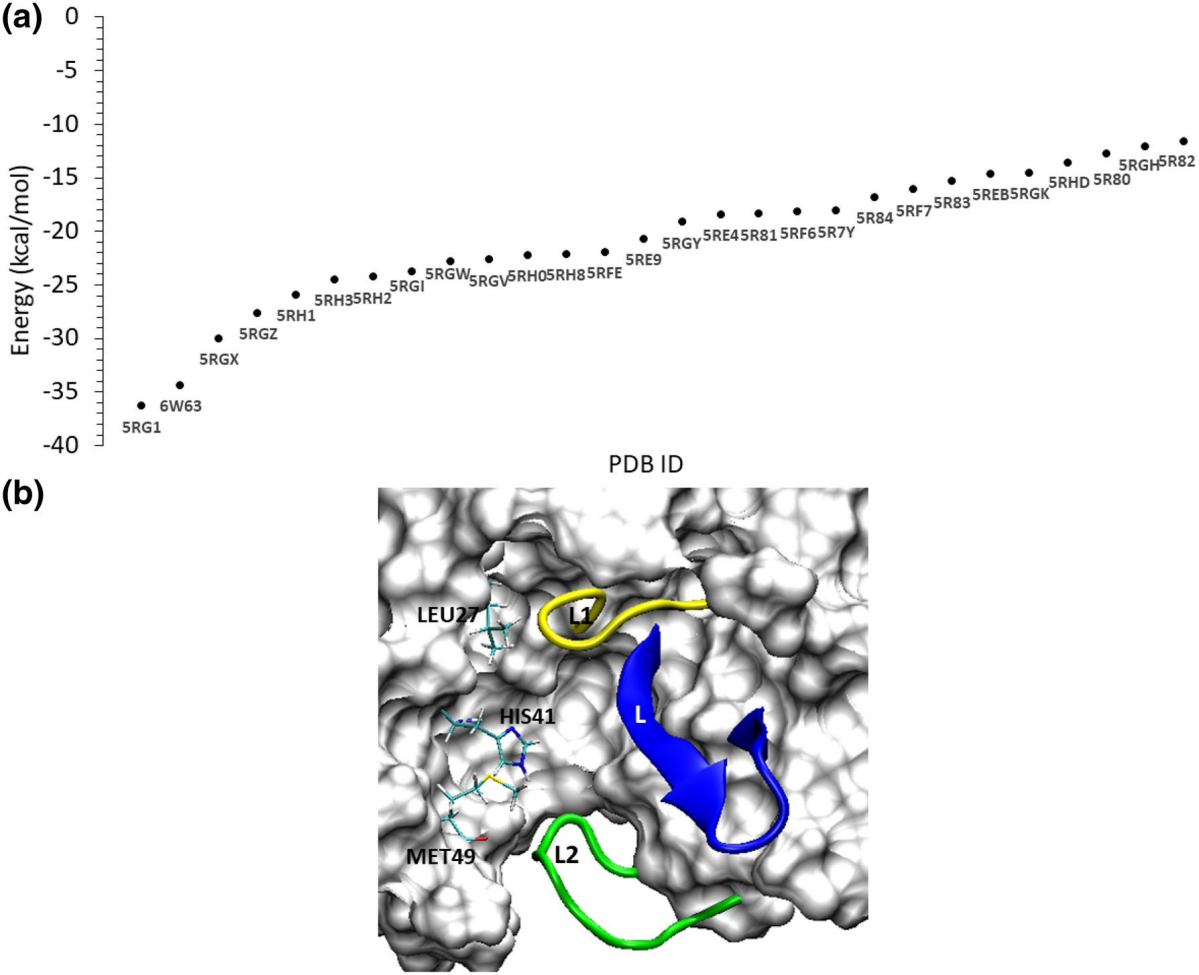
The scatter plot comparing the binding free energies of the ligand–M^pro^ complexes in which the ligands were bound in catalytic site (**a**) and the identification of four key components that are critical for ligand-binding in this site (**b**). Two loops flank the active site pocket: the L1 loop shown in yellow, and the L2 loop (or the domain II-III linker loop) shown in green. In addition, the ‘L’ β-sheet is shown in blue, and the residues such as HIS41, LEU27, and MET49 located deep inside the pocket are shown as stick representation. The molecular graphic in this figure was generated using VMD 1.9.3^63^ (**b**) while the scatter plot was generated using Microsoft Excel 365 (**a**) (https://www.office.com/).

Figure 4a compares the binding free energies of the complexes in which the ligands were bound within the catalytic site of M^pro^ throughout the simulation. From our analyses of MD trajectories of these complexes, it was clear that the ligand bound in the active site (or pocket 1) was mainly stabilized by their interactions with four segments, apart from the catalytic dyad residues, HIS41 (from domain I) and CYS145 (from domain II). These include (1) a loop (dubbed as ‘L1’) connecting the J and K β-sheets leading to CYS145, shown as yellow in Fig. 4b, (2) domain II-III linker (shown as green in Fig. 4b) composed of residues VAL186 through ALA193 and flanking on the other side of the active site (dubbed as ‘L2), (iii) The ‘L’ β-sheet (see in Fig. 1b) that is lining the active site and composed of residues HIS163-LEU167 (shown as blue in Fig. 4b), and (iv) key hydrophobic residues surrounding the catalytic HIS41 such as LEU27 and MET49. Depending upon how strongly the ligands engaged with these segments, their binding affinity scores varied. For example, the ligand, 5-fluoro-1-[(5-methyl-1,3,4-thiadiazol-2-yl)methyl]-1,2,3,6-tetrahydropyridine (PDB: 5RGH), was bound to the catalytic site of M^pro^ and had a weak binding affinity of − 12.10 kcal/mol. Analyzing this trajectory, we found that the fluorine-attached tetrahydropyridine ring was anchored to the ‘L1’ loop through the interaction of fluorine atom with that of GLN189 and GLN192 residues (supplementary Fig, SFig. 9a). However, the other methyl-attached thiadiazol moiety of the ligand remained flexible and underwent significant conformational changes during MD (supplementary Fig, SFig. 9b). As a result of this fluctuation in the ligand pose, the affinity of this complex remained low. In a similar nature, the ligands co-crystallized with M^pro^ in the complexes of 5R82 (6-(ethylamino)pyridine-3-carbonitrile), 5R80 (methyl 4-sulfamoylbenzoate), 5RHD (1-[4-(methylsulfonyl)phenyl]piperazine), 5RGK (2-fluoro-*N*-[2-(pyridin-4-yl)ethyl]benzamide), and 5REB (1-[(thiophen-3-yl)methyl]piperidin-4-ol) were also anchored to one of the four segments (in Fig. 4b) and remained dynamic within the catalytic binding site. Therefore, these complexes had a binding affinity > − 15 kcal/mol as seen in Fig. 4a. For example, the conformational dynamics of the ligand, 6-(ethylamino)pyridine-3-carbonitrile, in the 5R82 structure is clearly described in the supplementary figure, SFig. 10.

**Figure 5:**
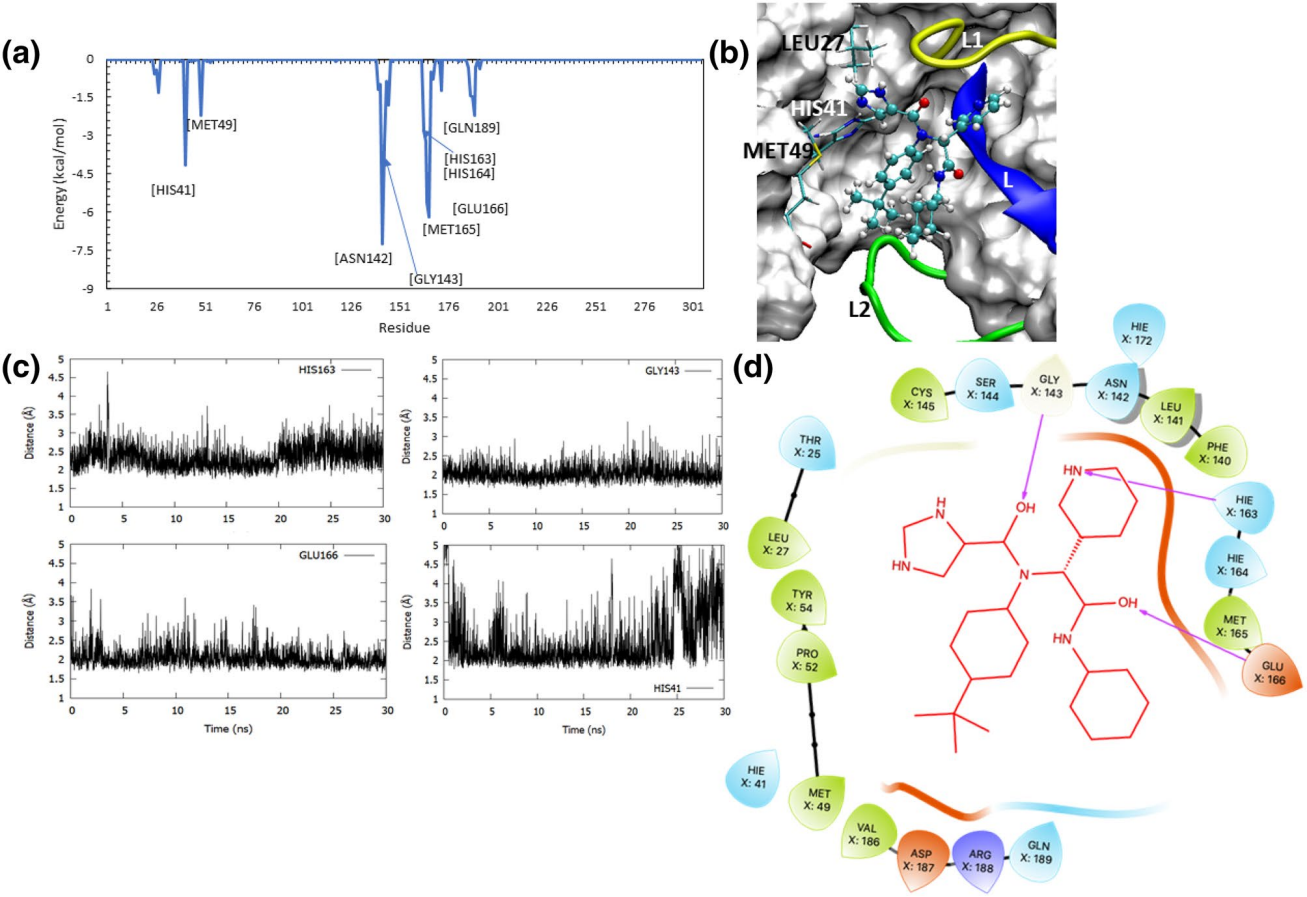
The pair-wise decomposition analyses (**a**) for the binding mode (**b**) of ligand in 6W63 complex and its hydrogen bond interactions with the key residues in the pocket (**c**), along with the 2D interaction diagram for the ligand–M^pro^ binding mode are shown. The decomposition plot (**a**) identified the key residues that helped in a favourable binding mode of the ligand (**b**) that was supported by its interactions with the L1 loop, L2 loop, L β-sheet and the catalytic diad residues. The ligand in this complex made stable H-bond interactions with a few key resiudes including HIS41, GLY143, HIS163, and GLY166 (**c**). In the presented 2D interaction diagram for the ligand–M^pro^ binding pose (**d**), the ligand is shown as red lines, the hydrophobic residues are shown in green; the polar residues in torquise; the negatively charged residues in orange; and the positively charged residues are shown in blue. The hydrogen bonds are displayed as purple arrows. The three dimensional molecular graphic used in this figure was generated using VMD 1.9.3^63^ (**b**) while the two-dimensional interaction diagram was developed using Maestro Schrodinger^86^ (**d**). The plots were generated using GNUplot (v5.2 patchlevel 8; http://www.gnuplot.info/) (**c**) and Microsoft Excel 365 (**a**) (https://www.office.com/).

**Figure 6:**
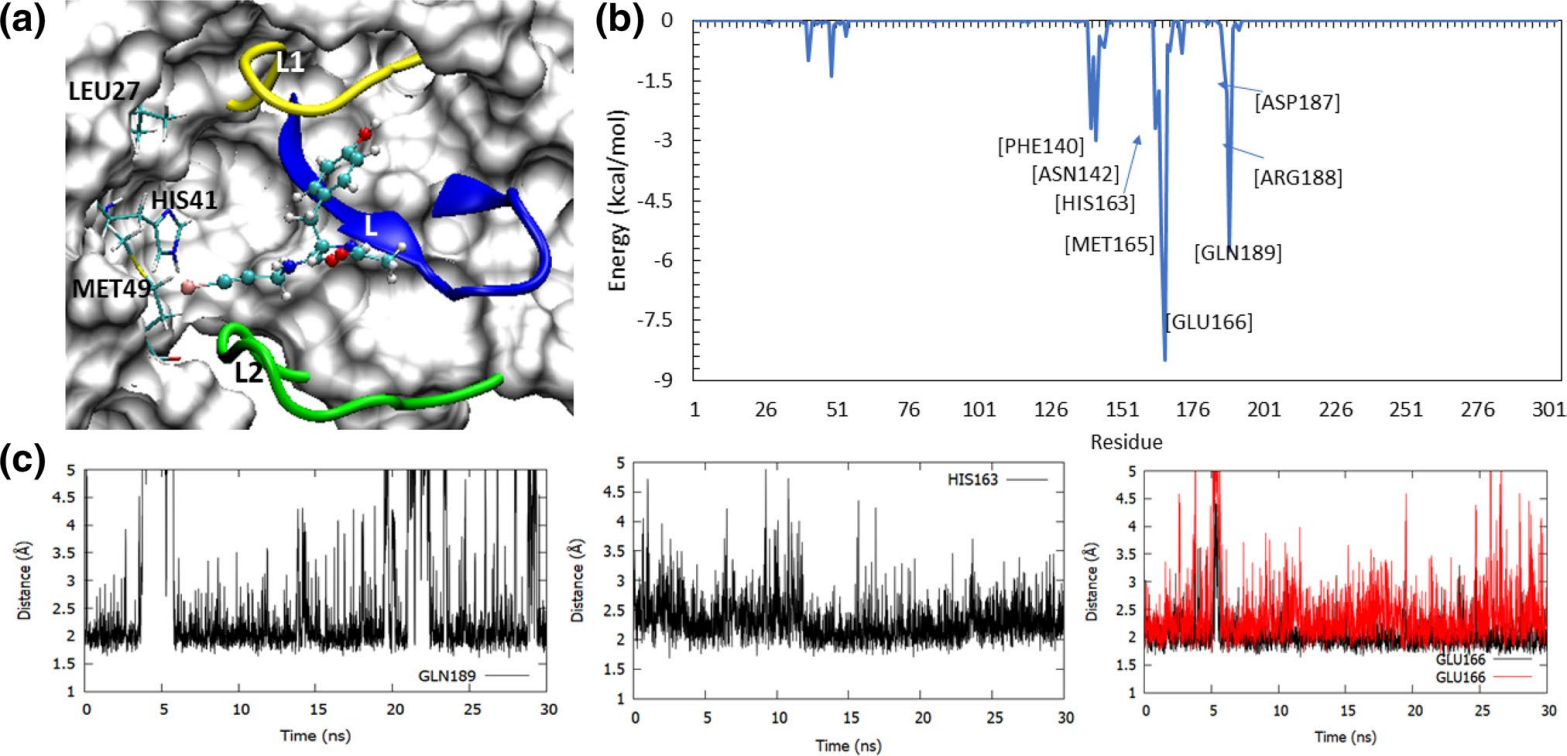
The binding mode of ligand in the 5RG1 complex within the catalytic site of M^pro^ (**a**), the decomposition of key residues contributing to this ligand–receptor complex (**b**), along with the time evolution of H-bond interactions of the bound ligand with residues such as GLN189, HIS163 and GLU166 (**c**). In the ligand bind pose in this complex (**a**), the ligand is seen interacting with all the key segments in the catalytic site of M^pro^, which include the L1 loop, L2 loop, L β-sheet and the catalytic diad residues. Evidently, the ligand occupied the lateral pocket in the catalytic site of M^pro^, where it formed stable H-bonds with HIS163 residue. This binding mode was supported by other key residues such as GLN189 and GLU166, as seen in the pairwise decomposition plot (**b**). The molecular graphic used in this figure was generated using VMD 1.9.3^63^ (**a**) while the plots were generated using GNUplot (v5.2 patchlevel 8 http://www.gnuplot.info/) (**c**) and Microsoft Excel 365 (**b**) (https://www.office.com/).

We noted that the ligands bound to the catalytic site and stabilized by the interactions with two of the four segments described above exhibited a better binding affinity score that is < − 15 kcal/mol (in Fig. 4b). For example, the bulky compound in the series studied here is *N*-(4-tert-butylphenyl)-*N*-[(1R)-2-(cyclohexylamino)-2-oxo-1-(pyridin-3-yl)ethyl]-1H-imidazole-4-carboxamide that was co-crystallized with M^pro^ in the PDB structure 6W63. This compound was bound against M^pro^ in a pose (in Fig. 5a–d), in which the pyridine ring occupied a lateral cavity formed by the ‘L1’ loop (yellow) and the ‘L’ β-sheet and formed a stable H-bond with HIS163 residue (Fig. 5c) in this cavity. The OH groups in this ligand made strong H-bonds with GLU166 and GLY143 (refer to the 2D interaction diagram shown in Fig. 5d). In addition, the imidazole ring interacted with HIS41, and the methyl group attached to cyclohexamine moiety made hydrophobic contacts with the ‘L2’ loop (i.e., the domain II-III linker loop). Pairwise decomposition analyses revealed the key residues that stablized this complex, which include HIS41, ASN142, GLY143, MET165, and GLU166. The interactions of the ligand with these key residues contributed at least − 3 kcal/mol energy to the binding free energy. Therefore, given the complementary properties between the ligand and the protein in the 6W63 structure, this complex had a high binding affinity score of − 34.4 kcal/mol. Unlike the compound in 6W63 complex, the Nalpha-acetyl-*N*-(3-bromoprop-2-yn-1-yl)-l-tyrosinamide ligand in the PDB structure of 5RG1 is a much more linear compound; yet, it displayed a strong binding affinity of − 36.30 kcal/mol towards M^pro^. It was found that this ligand was bound in a pose (Fig. 6a) that allowed it to interact with the key residues in the binding site (Fig. 6b). It was found that the phenol group in this ligand also filled the lateral pocket (Fig. 6a) through H-bonds with the imidazole side-chain of HIS163 residue (Fig. 6c). In addition, the ligand also formed two strong H-bonds with GLU166 (in Fig. 6c), and an additional H-bond with GLN189. Decomposition analyses identified HIS163, MET165, GLU166, ARG188, GLN189 as key energetic contributions to the binding free energy (Fig. 6b). This is in consistent with a few studies^21,35,54,81^ that noted a number of these residues (GLU166, GLN189, HIS163, SER144, GLY143) as key players in ligand interactions with M^pro^.

Similar to the ligands in 6W63 and 5RG1 complexes, several other pyridine-containing compounds in this study exhibited similar interactions and possessed a binding affinity value better than − 22 kcal/mol (Fig. 4a). These include (2R)-2-(3-chlorophenyl)-*N*-(4-methylpyridin-3-yl)propenamide (5RH3), 2-(3-cyanophenyl)-*N*-(4-methylpyridin-3-yl)acetamide (5RGX), 2-(3-cyanophenyl)-*N*-(pyridin-3-yl)acetamide (5RGZ), 2-(5-chlorothiophen-2-yl)-*N*-(pyridin-3-yl)acetamide (5RH1), (2R)-2-(3-chlorophenyl)-*N*-(4-methylpyridin-3-yl)propenamide (5RH3), 2-(3-chlorophenyl)-*N*-(4-methylpyridin-3-yl)acetamide (5RH2), 2-(5-cyanopyridin-3-yl)-*N*-(pyridin-3-yl)acetamide (5RGW), and *N*-(5-methylthiophen-2-yl)-*N*'-pyridin-3-ylurea (5RH0). In all these compounds, the pyridine ring occupied the lateral pocket and interacted with HIS163, whereas the linker group in their structures made H-bond interactions with ASN142, GLY143 and GLN189. For example, the binding mode of a ligand–enzyme complex in the PDB structure 5RH3, the corresponding H-bond evolution plots, and energy decomposition graphs are all provided in the supplementary figure SFig. 11. Unlike the pyridine-based compounds, the ligand co-crystallized in the PDB structure 5RGV, 2-(isoquinolin-4-yl)-*N*-phenylacetamide, also had a high affinity of − 22.65 kcal/mol; but, in this structure, it was an isoquinoline group that formed a H-bond with HIS163 and occupied the lateral pocket. This shows that the lateral pocket can engage different moieties such as pyridine, phenol and isoquinoline groups. A previous study^81^ noted that the H-bond interactions between different covalent and non-covalent ligands with two conserved hydrophobic residues HIS163 and GLU166 in M^pro^ and suggested a possible role for these residues in viral polypeptide cleavage process.

The other molecules (in Fig. 4) in the binding affinity range of − 15 and − 22 kcal/mol include ligands that formed stable interactions with the ‘L2’ loop, ‘L’ β-sheet and also with ‘L1’ loop, but, they did not occupy the lateral cavity. For example, the 1-methyl-3,4-dihydro-2 ~ {H}-quinoline-7-sulfonamide ligand (in 5R81), interacted with the ‘L2’ loop through H-bonds with GLN190 and GLY192, and with the ‘L’ β-sheet through hydrophobic interactions with MET165 and GLU166, in addition to the interactions with HIS41 (supplementary figure, SFig. 12). However, this ligand did not engage with the lateral pocket and consequently had a weaker binding affinity of − 18.3 kcal/mol. In a similar nature, ligands including 5-(1,4-oxazepan-4-yl)pyridine-2-carbonitrile (5RF6), *N*-[(4-cyanophenyl)methyl]morpholine-4-carboxamide (5REE), 2-(4-methylphenoxy)-1-(4-methylpiperazin-4-ium-1-yl)ethenone (5RE9), *N*-(4-methoxypyridin-2-yl)-2-(naphthalen-2-yl)acetamide (5RGY), and *N*-(2-phenylethyl)methanesulfonamide (5R7Y) interacted with different segments within the binding site without occupying the lateral cavity and all of them displayed weaker binding affinity toward M^pro^. While our MM-GBSA analyses suggested that ligand engagement with the lateral pocket in M^pro^ improved the binding affinity, the ligand complex of 5RF7 was an exception. The ligand in this complex, 1-(4-methylpiperazin-1-yl)-2-(1H-pyrrolo[2,3-b]pyridin-3-yl)ethan-1-one, contained a pyrrolopyridine moiety that was indeed bound in the lateral pocket of M^pro^ and formed H-bonds with HIS163. However, the methylpiperazine group remained flexible and formed only transient H-bonds with the key residues such as GLY143, ASN142 and GLU166 (supplementary figure, SFig. 13). It can be noted that although this ligand did not make stable interactions with the key residues in the catalytic pocket such as MET49, GLY143, SER144, MET164, and GLU166 (SFig. 13b), it still had an affinity of ~ − 16.1 kcal/mol that was mainly due to the stable engagement of the ligand with the lateral pocket in M^pro^. Again, this highlighted the important role of the lateral pocket in the ligand M^pro^ interactions.

In order to verify the stability, molecular interactions and binding affinity of the ligand–M^pro^ complexes over a long simulation time, we extended the MD simulation of the selected systems for upto 100 ns. All the parameters for the extended MD remained the same as used in the 30 ns long simulation. Only those complexes that remained stable and had a binding affinity score lower than − 20 kcal/mol (in Fig. 4a) during the initial shorter simulation (of 30 ns) were subjected to the longer MD runs. This choice was made as the compounds that either unbound or displayed weaker binding affinity to M^pro^ during the 30 ns trajectory may not benefit from the extended simulation. Therefore, we collected 100 ns long MD trajectories for a total of 15 systems, which included the apo M^pro^ and 14 ligand-bound M^pro^ complexes (the list is provided in supplementary table, ST4).

From our analyses of the 100 ns long MD trajectories, we found out that the 2-(4-methylphenoxy)-1-(4-methylpiperazin-4-ium-1-yl)ethenone ligand in 5RE9 structural complex became unstable during the extended simulation and unbound from the binding pocket of M^pro^. It should be noted that, amongst the compounds selected for longer simulation, this ligand in 5RE9 complex displayed the weakest affinity of − 20.69 kcal/mol against M^pro^ during the 30 ns MD trajectory. In fact, as discussed earlier, the binding pose of this ligand did not involve interactions with the lateral cavity in M^pro^. Therefore, it was not surprising to find this ligand unbinding from M^pro^ binding site during the extended simulation. Except for this one complex, all the other ligands remained stably bound against the M^pro^ (RMSDs provided in the supplementary table ST4). The binding affinities of these stable complexes were recalculated using the final 80 ns long MD trajectories by collecting snapshots every 20 ps. A comparison of the binding affinities of the complexes from 30 ns (last 10 ns) and 100 ns (last 80 ns) MD trajectories are provided in the supplementary figure, SFig. 14. As it can be seen, the binding affinities of most of the complexes (except for a very few) calculated from the longer trajectories were close to their respective values from the shorter trajectories. The ΔΔG_(bind)_ values (i.e., the absolute difference between the binding free energies from 30 and 100 ns trajectories) of the complexes mostly remained within 3 kcal/mol, which is well within the estimated standard deviations. For example, we found that the 6W63 structure with the bulkiest ligand in this study, (*N*-(4-tert-butylphenyl)-*N*-[(1R)-2-(cyclohexylamino)-2-oxo-1-(pyridin-3-yl)ethyl]-1H-imidazole-4-carboxamide), remained stable throughout the 100 ns long MD simulation and retained all the key interactions in the active site of M^pro^ (as discussed above). In particular, the H-bond interactions between the pyridine ring of the ligand and HIS163 in the lateral cavity of M^pro^ were intact for the entire simulation. As a result, the binding affinity of this complex (6W63) slightly improved by ~ 2 kcal/mol in the calculations with the longer MD trajectory when compared to the shorter one. Similarly, in most other complexes where the key interactions with the binding site, especially the lateral cavity of M^pro^, remained stable during 100 ns simulation, their binding affinity scores also remained fairly strong (< − 20 kcal/mol). Nevertheless, the binding affinities of three complexes such as 5RG1, 5RH2, and 5RH0 became weaker by > 4 kcal/mol (ΔΔG_(bind)_ value) when calculated from the extended MD trajectories. Analyses of their trajectories revealed that the ligands bound in these complexes initially interacted with the lateral pocket but eventually lost them to the dynamic changes that occurred after 50 ns time scale. Yet, the compounds remained bound in the active site of M^pro^ for the entire duration of 100 ns long MD. Thus, our extended MD simulation and analyses also demonstrated the importance of a strong interactions of the ligand with the lateral pocket (in particular to HIS163) of M^pro^ to maintain a relatively stronger binding affinity.

### PCA analyses of apo and ligand-bound M^pro^ complexes

The 100 ns long trajectories of the apo and the 14 ligand-bound M^pro^ complexes were used to perform principal component analyses (PCA) to understand the impact of ligand-binding on the atomic fluctuations of M^pro^. For this purpose, we initially combined all the 15 trajectories into one Grand ensemble of 75,000 configurations (5000 snapshots for each system) and superposed them based on 168 Cα-atoms belonging to secondary structure elements (see methods section). Aside from short explorations, the time evolution of RMSD revealed that most of the fluctuations deviated between 1–2 Å from the ensemble’s average structure (Supplementary figure, SFig. 15a). We analyzed the trajectory average RMSD for all the systems and found out that it was the highest for the apo protein (Supplementary figure, SFig. 15b), suggesting that the apo trajectory deviated the most from the average structure, which was determined by liganded trajectories in proportion 14:1. Thus, the ensemble average structure might be defining a characteristic ‘bonded conformation’, which was apparently distinctive than that of the apo state. The two liganded trajectories that deviated the most from the average were 5RE9 and 6W63 complexes (Supplementary figure, SFig. 15b). As discussed above, the ligand of 5RE9 structure displayed weak affinity towards M^pro^ and unbound from the target during the 100 ns long MD simulation. As a result, the deviations in this complex followed an apo-like trajectory. On the other hand, the ligand in 6W63 is a large and multi-moiety molecule, so that it exhibited some variations with respect to the average structure. The residue mean square fluctuation (MSqF) profile for the Grand ensemble showed that active-site loops including L2 (i.e., the domain II-III linker loop), and the loop formed by P39-Y54 (dubbed as L3 in the text hereafter), along with the loops at domain III (L_dIII_) fluctuated the most (Supplementary figure, SFig. 15c). The trace of L3 residues in 5RE9 and 6W63 structures were indeed the ones with higher atomic displacements from the average conformation. It should be noted that 5RE9 complex presented the lowest MM-GBSA binding energy (− 8.65 kcal/mol), and the 6W63 complex displayed the highest binding affinity (− 36.56 kcal/mol) of the dataset studied using 100 ns long MD trajectories.

**Figure 7:**
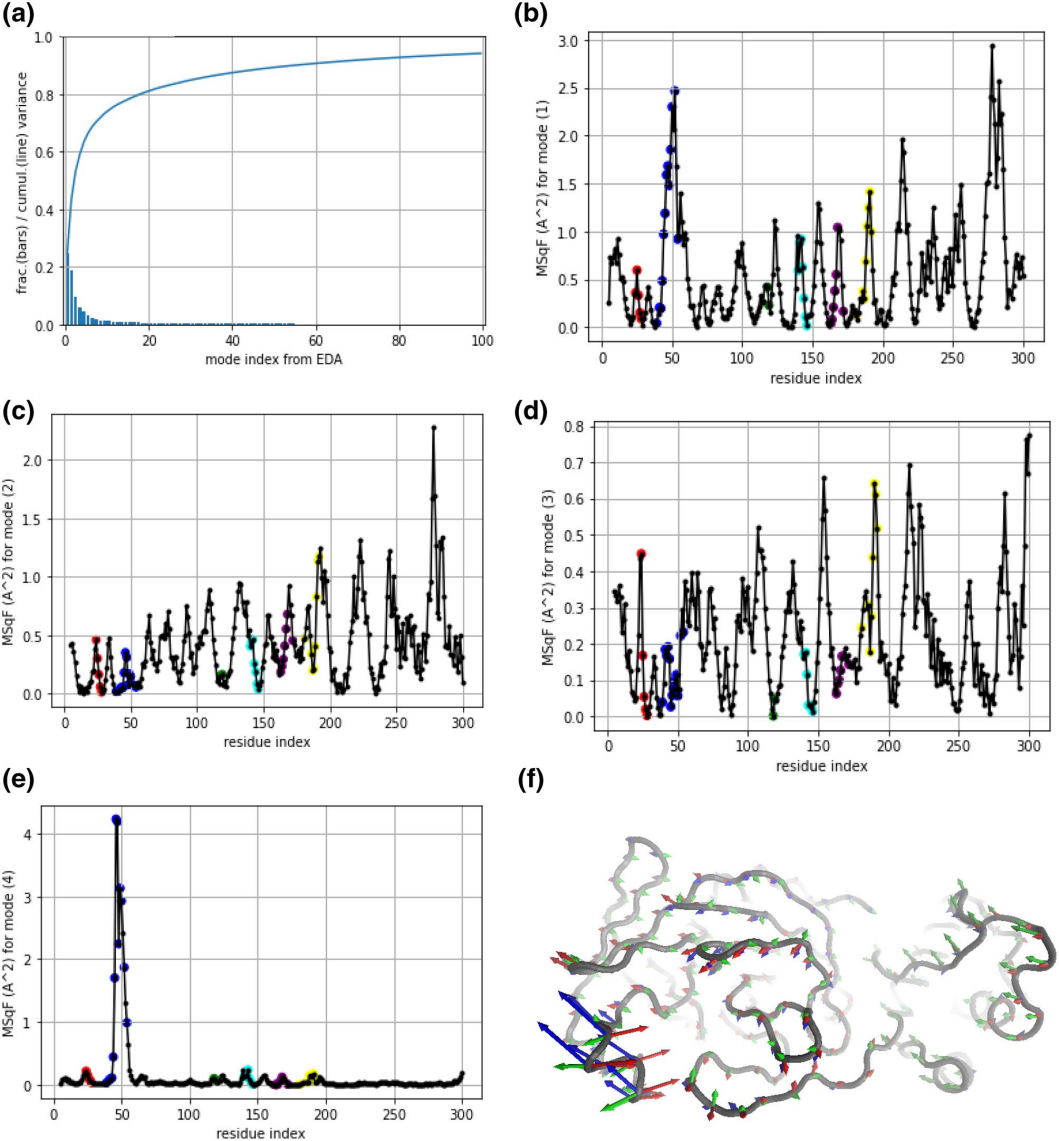
(**a**) EDA variance of the atomic fluctuation in the combined ensemble. Fractional variance (blue bars) and cumulative fractional variance (blue curve) of the atomic fluctuations for the first 100 PC modes from an EDA over the Grand ensemble of MD conformations. (**b**–**e**) Mobility profile per mode in the combined ensemble. Dissection of the MSqF (in Å^2^) for the first 4 PC modes. Highlighted with colors residues belonging to active-site loops according to segment [24–28] (red), L3: [39–54] (blue), segment [118–119] (green), L1: [140–146] (cyan), “L” strand: [163–172] (purple), L2: [181–192] (yellow). (**f**) MD Grand ensemble deformations into the essential space. Tube representation of the Cα-trace of M^pro^ (residues 5..300) depicting vectors that characterize the direction and relative magnitude of the deformations determined by the indicated PC modes: PC1 (blue), PC2 (red), and PC3 (green). Each set of vectors scaled to 1 Å of $\left\langle {{\text{RMSF}}} \right\rangle$ for visualization. The molecular graphic used in this figure was generated using ProDY^74^ and rendered using VMD 1.9.3^63^ (**f**) while the plots were created using Microsoft Excel 365 (**a**–**e**) (https://www.office.com/).

An essential dynamics analysis (EDA)^82^ was performed for the Grand ensemble for the Cα-atoms to determine the main modes of deformations of M^pro^ in solution. Out of the total of 879 principal components (PCs) analyzed, the first 4 PCs captured ~ 60% of the total variance in atomic fluctuations (Fig. 7a). The dissection of the total MSqF distribution per mode of fluctuation (or per PC) for the first 4 PCs is shown (Fig. 7b–e). Note that the first 3 PCs exhibited high collectivity (Fig. 7b–d), with peaks of fluctuation at and around loops connecting stable secondary structural elements; while the fluctuations described by PC4 is highly localized at L3 loop with a maximum at Ser46 (Fig. 7e). The combined PC[1:3] fluctuations corresponded to a complex deformation along the vectors shown in Fig. 7f that reconfigured the binding cavity and also elicited a displacement of loops in domain III (L_dII_). This describes that the dynamics of the active-site loops remains a key factor in determining the stability of the complex, and therefore, the binding energy.

Nevertheless, to better solve the fluctuations of M^pro^ elicited by ligands at the binding pocket on each trajectory, the 100 ns MD trajectories of the 15 systems (apo and 14 ligand-bound complexes) were independently superposed based now only on 82 Cα-atoms belonging to β-strand elements of domains I and II and an EDA were performed. The calculated MSqF profiles for each trajectory (shown in Supplementary figure, SFig. 16) displayed diverse amplitudes and collectivity. However, the dominant fluctuations of the active site L3 loop and the DII-DIII linker loop (L2) were seen as a common feature in almost all of the trajectories. Further investigation of the PCs relative to the domains I and II of M^pro^ in the individual trajectories (Supplementary figure, SFig. 17) indicated that the related essential dynamics (up to 71% of variance) were mainly captured by the first 4 PC modes in almost all the cases. Inclusion of the first 20 PC modes were able to capture up to 84% of the variance in the systems studied. Again, the L3 loop fluctuations were clearly seen in the individual trajectories, therefore, highlighting the role of this loop in active site of M^pro^.

In order to compare part of the essential dynamics among the different trajectories with respect to D_I/II_ mobilities, the space overlap between PC[1:4] of any trajectory with PC[1:3] of any target trajectory were calculated. This can report the grade of superposition of the subspace covered by both trajectories in terms of the inner products between pairs of their deformation vectors. From the heatmap matrix of the 15 ensembles (Supplementary figure, SFig. 18), it can be observed that subspace covered by 6W63 complex was the most unique, while the subspace covered by 5RE9 is the most shared by the dataset. This is in agreement with the trends observed in the PCA of the Grand ensemble and our binding affinity data. Since the ligand in 6W63 was strongly bound in the binding site of M^pro^, it displayed a unique atomic fluctuation arising from ligand engagement. Conversely, the ligand in 5RE9 complex is the most exposed in the selected dataset and this ligand unbound from the target during the extended MD simulation. As a result, the modes of fluctuation in 5RE9 are similar to the behaviour seen for the ligand-free apo M^pro^.

### Steric water site analyses in the lateral pocket of M^pro^

**Figure 8:**
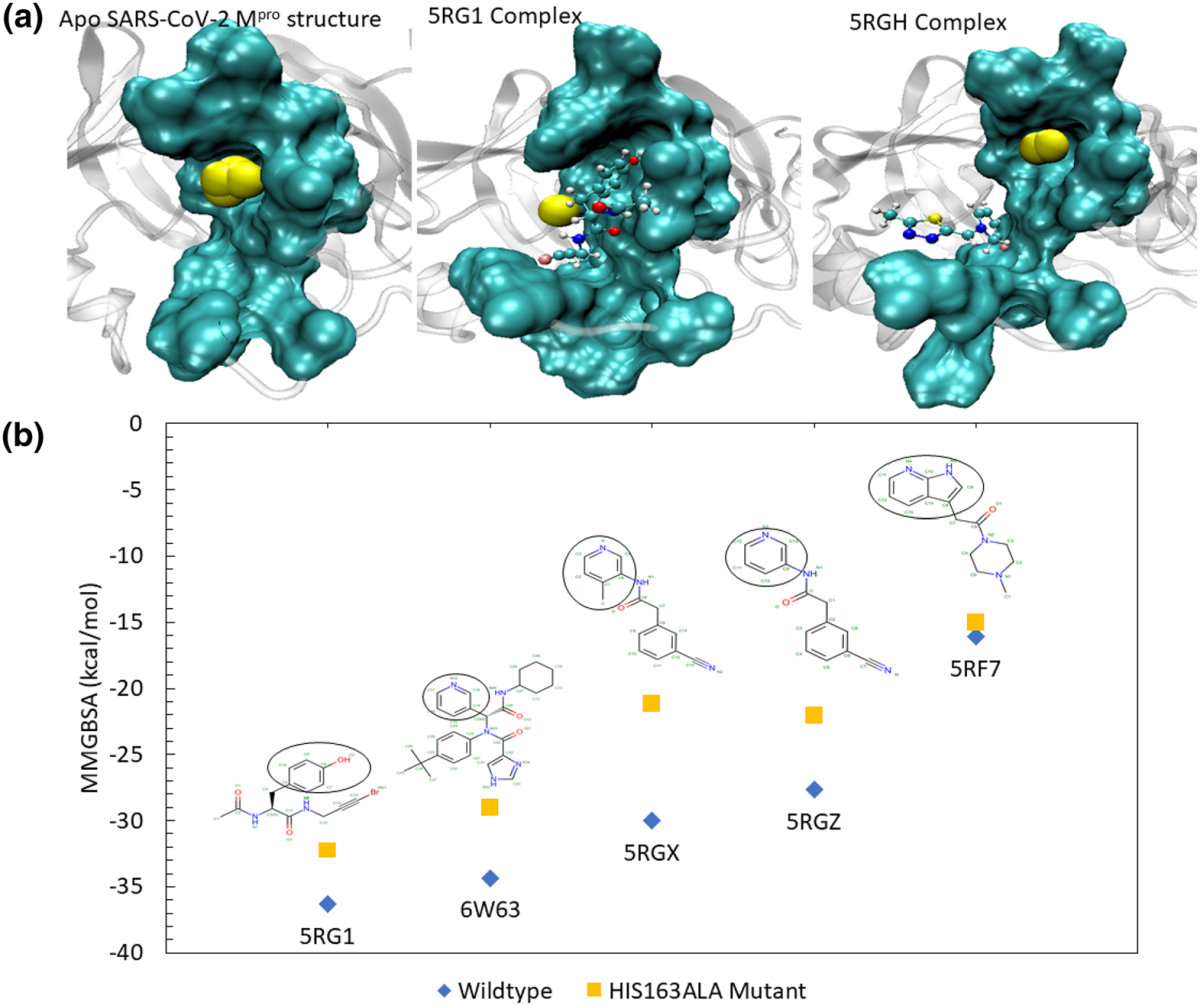
The identification of steric water sites in the apo and ligand-bound M^pro^ complexes (**a**) along with the comparison of the binding free energy of the wildtype (WT) and HIS163ALA mutant complexes of the select systems (**b**). (**a**) The presence of steric water molecules (shown as yellow spheres) are found within the lateral binding pocket in the apo protein structure. When a ligand occupied the lateral site in the 5RG1 complex, the steric water molecules were displaced to the edge of the pocket, allowing for more stable ligand-binding. Nevertheless, when the ligand did not occupy the lateral pocket (as in the case of 5RGH structure), the steric water molecules are again observed within this lateral site. (**b**) Mutation of HIS163 to an alanine (ALA163) clearly led to a weak binding affinity between the selected ligand–M^pro^ complexes. The molecular graphic in this figure was generated using VMD 1.9.3^63^ (**a**) while the comparison plot in (**b**) was generated using Microsoft Excel 365 (https://www.office.com/).

Since our MD and binding free energy analyses highlighted an important role of the lateral pocket in the ligand–M^pro^ affinity, we analyzed the steric water site in the apo M^pro^ structure. This lateral pocket is composed of residues ASN142, GLY143, SER144, CYS145, HIS163, HIS164, MET165, and GLU166. Our analyses revealed that this pocket, in the absence of a ligand, was occupied by steric water molecules (Fig. 8a). Next, we analyzed the presence of steric water molecules in the lateral pocket when a ligand was bound in M^pro^. For example, in the PDB structure of 5RG1, the ligand occupied the lateral pocket and the steric water site was then shifted to the edge of the pocket (Fig. 8a). This displacement of water allowed the ligand to interact with the pocket and bind the target with enhanced binding affinity. In the case of ligands that did not fully occupy the lateral pocket, for example, the PDB structure of 5RGH, the binding affinity was seen to be weaker and, now, the steric water molecule site was again found centered within the lateral pocket (Fig. 8a). This site likely favours the presence of an electronegative hydrogen bond-forming moiety in whose absence this space was occupied by a water molecule that substitutes this role. This evidence supplements the significant role of the lateral pocket in ligand engagement and stronger binding energies.

### Computational mutagenesis of HIS163 in the lateral pocket

Our results significantly highlighted the importance of the lateral pocket in M^pro^, in particular the role of HIS163 in stabilizing the ligand–M^pro^ complexes. Therefore, to evaluate this finding, we mutated HIS163 with ALA163 (called the HIS163ALA) in the selected M^pro^ complexes and reperformed 30 ns long MD and MM-GBSA calculations as carried out earlier for the WT complexes. For these mutagenesis calculations, we selected five complexes (listed in the supplementary table, ST5) related to the PDB structures 5RGZ, 5RF7, 6W63, 5RG1, and 5RGX and mutated HIS163 with ALA163 in the protein structure of each of these complexes. These ligand structures and their wildtype and mutated MM-GBSA binding affinities are shown in Fig. 8b. As can be seen in this figure and the supplementary table, ST5, the HIS163ALA mutation caused an MM-GBSA difference of up to 9 kcal/mol. For example, the PDB structure of 5RGX complex showed the greatest difference in binding affinity with a decrease of 8.868 kcal/mol. In this complex, a methyl-attached pyridine ring was occupying the lateral pocket. In the WT complex, shown in supplementary figure, SFig. 19, the nitrogen atom of the pyridine ring formed a strong H-bond with HIS163. The residues, GLY143, SER144, and CYS145, all formed electrostatic interactions with the double-bonded oxygen atom of the ligand, and ASN142 and MET165 greatly contributed to the ligand’s binding affinity via Van der Waals (VDW) interactions. In the HIS163ALA mutant complex, the nitrogen atom was no longer able to form a stable interaction with HIS163 (SFig. 19b), dropping the pairwise residue energy from − 3.68 kcal/mol to − 0.76 kcal/mol and enabling the pyridine ring to become much more flexible throughout the trajectory. In addition to the HIS163 interaction loss, the 5RGX mutant complex also lost its strong ASN142, GLY143, and SER144 interactions, and instead formed transient contacts with CYS145 and GLU166. This significant change in pairwise residue contribution accounted for the 8.868 kcal/mol loss in MM-GBSA binding affinity, as clearly shown in SFig. 19c.

The 5RGZ HIS163ALA mutant complex also showed a significant decrease in binding affinity. This complex had an initial MM-GBSA of − 27.656 kcal/mol which was decreased by 5.582 kcal/mol in the mutated form. The ligand structure in the 5RGZ complex is very similar to that of the 5RGX complex as previously described, with the absence of a methyl group attached to the pyridine ring positioned in the lateral pocket (Supplementary figure, SFig. 20). Although these ligands are very similar in their chemical structures, they behaved differently when subjected to the HIS163ALA mutation. The 5RGZ mutant complex initially adopted a pose much like its WT complex. During the first 15 ns of the MD trajectory, the pyridine ring was found well within the lateral pocket. After 15 ns of simulation, the 5RGZ complex adopted a new pose in which the pyridine ring moved significantly out of the lateral pocket to instead interact with LEU27, HIS41, SER46, and MET49 (SFig. 20).

Complexes 5RG1 and 6W63 (the top MM-GBSA scoring complexes in this study) were also mutated due to their initially strong binding affinities with MM-GBSA values of − 36.3341 kcal/mol and − 34.3563 kcal/mol, respectively. The 5RG1 complex contained a phenol group in the lateral binding pocket, where the oxygen atom formed a strong H-bond with HIS163. The 6W63 complex contained a very large ligand, with a pyridine ring moiety located in the lateral pocket, as discussed earlier. In addition to its interactions with HIS163, in the WT complex, the 5RG1 complex formed stabilizing electrostatic interactions with GLU166 and GLN189. When HIS163 was mutated, the MM-GSBA binding affinity of the 5RG1 complex decreased by 4.172 kcal/mol. This is attributed to the loss of the HIS163 interaction with the phenol group and GLN189 interaction. In the mutated complex, GLU166 maintained its hydrogen bonds with the ligand and formed an additional, but transient, electrostatic interaction with the mentioned phenol group. This additional interaction to stabilize the phenol group was seen in the pairwise decomposition graph in Supplementary figure, SFig. 21 and also reflected in the slightly lessened decrease in MM-GBSA binding affinity in comparison to other complexes such as 5RGX and 5RGZ. In the WT 6W63 complex, the pyridine ring moiety and nearby double-bonded oxygen atom formed strong, electrostatic interactions with HIS163, ASN142, and GLY143. When mutated, the double-bonded oxygen atom lost its stabilizing electrostatic interactions, and the HIS163 H-bond with nitrogen atom in the pyridine ring was replaced by an H-bond with CYS145, decreasing the binding affinity of the complex by 5.339 kcal/mol. Since the 6W63 ligand is a bulky compound, the complex can be stabilized in the binding site and lateral pocket by its other important electrostatic and VDW interactions (SFig. 22).

We also assessed the effects of mutation on the PDB complex of 5RF7 by building a HIS163ALA mutant model. This complex has a unique 7-azaindole, a double-ringed moiety that fits in the lateral pocket. In the WT 5RF7 complex, this moiety formed stable H-bonds with both HIS163 and HIS164. The rest of the ligand was unable to form lasting interactions with the protein residues and oscillated between ASN142/GLY143 and GLU166. In the HIS163ALA mutant, although the HIS163 bond was lost, the strong bond with HIS164 was maintained. In addition, the ligand was positioned so that it interacted stronger with GLU166. The gain of this interaction is evident in the residue decomposition graph in Supplementary figure, SFig. 23. Since the mutated 5RF7 complex can maintain key, stabilizing interactions, specifically in the lateral pocket, its MM-GBSA binding affinity was only decreased by ~ 1.08 kcal/mol. Therefore, our computational mutagenesis analyses demonstrate the importance of HIS163 and its role in ligand engagement in SARS-CoV-2 M^pro^.

### Effects of explicit water molecules in the binding affinity of ligand–M^pro^ complexes

**Figure 9:**
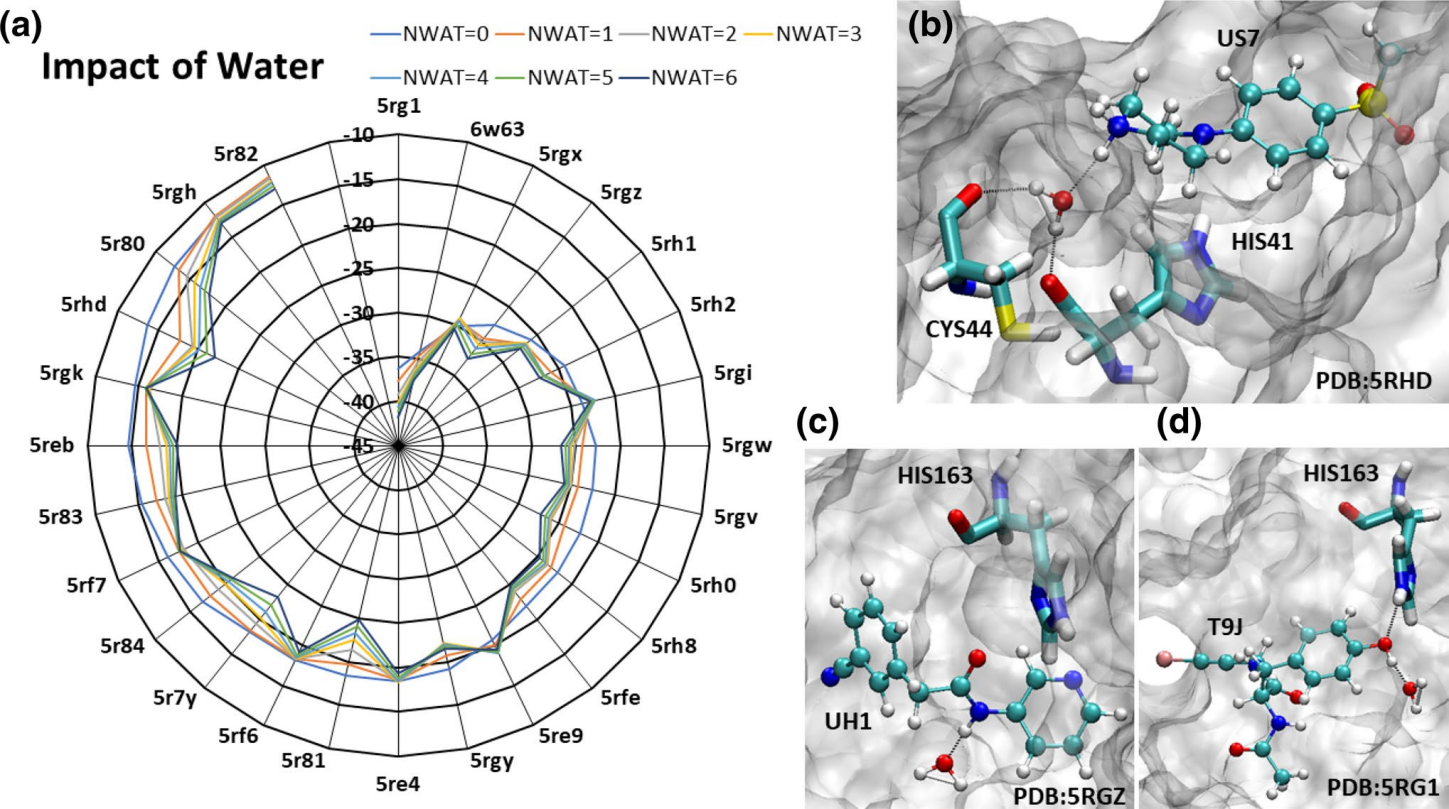
A polar plot describing the change in binding affinity scores of the select ligand–M^pro^ complexes in response to the presence of varying numbers of explicit water molecules (NWAT = 0–6) (**a**), and the 3D snapshots describing the different water interactions with the ligand (**b**–**d**) are also shown. In the 5RHD complex, a water molecule bridged the protein and the ligand acting as a proton donor to M^pro^ and proton acceptor for the ligand (**b**). In PDBs 5RGZ and 5RG1, a key water molecule interacted with the carboxamide and hydroxyl side chains of the ligand that helped to maintain the association of the ligands with the lateral pocket (**c**,**d**) in M^pro^. The molecular graphics in the figure were generated using VMD 1.9.3^63^ (**b**–**d**) while the radial plot in (**a**) was generated using Microsoft Excel 365 (https://www.office.com/).

Our binding free energy analyses revealed several key interactions that contribute to the binding affinity of the reversible ligands against M^pro^. It is known that water molecules play an important role in the catalytic process of M^pro^ in SARS-CoV-2, as also confirmed by our steric water site analyses. For example, a recent study^83^ based on X-ray crystallography and in vitro enzyme kinetics reported water-mediated interactions of known inhibitors such as leupeptin and telaprevir with key residues such as GLN189, and GLN192. Therefore, we analyzed the effects of explicit water molecules on the binding free energies of the M^pro^ complexes, in which the ligand was bound within the catalytic binding site (or pocket 1). For this purpose, we employed the NWAT-MM-GBSA variant, as described earlier by Maffucci et al.^73^ and explained in the methods section. We estimated the MM-GBSA energies with the presence of 0 to 6 explicit water molecules (noted as NWAT = 0 to 6 in the text). The calculated NWAT-MM-GBSA values for the complexes studied are provided as a polar plot in Fig. 9a and the corresponding values are listed in the supplementary table, ST6. In the polar chart (in Fig. 9a), the angular axis (along the circle) presents the PDB codes of the complexes studied, and the radial axis (connecting the circle to the center of the plot) presents the binding affinity values in kcal/mol. The MM-GBSA energies for the systems with no explicit water (i.e., NWAT = 0) is the outer polar line shown in blue in Fig. 8a. The MM-GBSA energies with the presence of (1–6) explicit water molecules are also shown as data points connected by polar lines in different colors. For any given system, if the presence of a water molecule enhances the affinity, then the polar data points for that system is expected to move inside towards the center. Whereas, if the water molecule did not make any impact, then the polar data points are expected to cluster at the same position. As can be seen in Fig. 9, we noticed that most of the systems had the impact of explicit water molecules. The complexes represented by the PDB structures 5RHD, 5R7Y, 5R81, 5RGZ, and 5RG1 clearly had a significant impact by the presence of explicit water molecules, as the data points for these systems moved inward with the increase of each water molecule. This suggested that the increase in binding affinity (lower MM-GBSA scores) is associated with increasing numbers of explicit water molecules. For example, the maximum impact of water molecules on binding affinity was seen for the 5RHD complex, where the binding affinity scores increased by ~ 8 kcal/mol (from − 13.63 kcal/mol for NWAT = 0 to − 22.09 kcal/mol for NWAT = 6). Analyzing the structures (in Fig. 9b), it can be noted that the water molecules bridged the bound ligand **(**1-[4-(methylsulfonyl)phenyl]piperazine) with CYS44 and HIS41 residues during MD. The other water molecules were generally found engaging with the sulfonyl and piperzine groups. Similarly, for the high-affinity complexes such as 5RGZ and 5RG1, the inclusion of explicit water molecules further enhanced their affinity towards M^pro^ (Fig. 9a). As expected, the water molecules were found to stabilize the electronegative sites such as amide, nitriles, or the hydroxyl groups in the ligands co-crystallized with these complexes (Fig. 9c,d). In some other complexes such as those represented by the PDB codes 5RF7, 5RGK, 5RH8, 5RGV, adding one or two water molecules enhanced the ligand–enzyme affinity; however, adding more water molecules did not make any more impact on the affinity. As a result, the binding affinity scores for these complexes with NWAT > 2 all clustered at one position suggesting the saturation in the number of water molecules that can engage inside the catalytic site of M^pro^. Thus our NWAT-MM-GBSA analyses suggested that up to 2 water molecules could play an important role in the interactions of ligand–M^pro^ in the complexes studied in this work. Nevertheless, we were also able to see that some complexes did not have any impact from the explicit water molecules such as the complexes of 5RGX and 5RE9. As the ligands bound in these complexes were already engaged in optimal interactions with the residues in the pocket, therefore, the water could not contribute to the ligand–protein interactions (supplementary figure, SFig. 24).

### Ligand interactions with a M^pro^ dimer

As we discussed above some of the ligands bound to the monomer form of M^pro^ did not exhibit stable interactions in MD and egressed the protein during simulations. It is now known that M^pro^ is functional in a dimeric state, where the N-finger of the second monomer interacts with the first monomer chain to close its catalytic site. Therefore, the natural question is if the ligands that egressed M^pro^ during our simulation were due to the absence of the involvement of the second chain of M^pro^. Therefore, to address this concern, we analyzed the dynamic interactions of the select ligands in the dimeric state of M^pro^. These include the ligands that were originally bound (1) within the catalytic site of M^pro^ and unbound during MD, (2) at the dimer interface of M^pro^ (e.g., 5RGQ and 5RFA complexes), and (3) at the proximity to the dimer interface. Using these selection criteria, a total of 13 complexes were selected for evaluation in the dimer system. Supplementary figure, SFig. 25, presents the positions of all the selected ligands within a dimer structure of M^pro^ and the PDB codes for the corresponding ligands. For each of the complexes selected, we modeled the dimer by aligning a ligand-bound monomer against the PDB structure of a SARS-CoV-2 M^pro^ dimer (PDB 6WTT) and deleting an aligned chain of the overlapping monomer. The modeled ligand-bound dimer complexes were subjected to 30 ns long MD simulation and their binding free energies were recalculated using the MM-GBSA method. The MM-GBSA scores of the select complexes both under monomer and dimer conditions are compared in the supplementary table, ST7. We initially assessed the stability of the complexes, in which the ligands were bound at the proximity to the dimer interface. These include 5RF1, 5RE8, 5RF9, 5RE7, 5RGJ,5REZ (refer to ligand positions in SFig. 25. As can be seen in the supplementary table, ST7, the presence of a M^pro^ dimer does not increase the stability or affinity of these complexes when compared to their respective monomer M^pro^ complexes. These ligands are still unbound from the dimer M^pro^ structures. This highlights the likely weak affinity of these ligands towards M^pro^. Interestingly, in the case of 5RF1 complex, the ligand exhibited better affinity (~ 5.7 kcal/mol) in the monomer state when compated to that of the dimer form. From our analyses, we noted that, during the MD simulation of the monomer state, the ligand in this complex moved away from its initial crystal structure pose and occupied the surface near the DI-DIII linker loop thereby resulting in a weak affinity of ~ 9 kcal/mol (supplementary table, ST7). Nevertheless, in the dimer form, this linker loop and DIII were involved in the dimer interactions that prevented the ligand from occupying the surface near DI-DIII linker (as in the case of monomer state). As a result, the ligand eventually completely unbound from the protein and moved into the bulk water. As a result, the weak affinity that the ligand had with the M^pro^ monomer was further reduced by ~ 5.7 kcal/mol. In a few other complexes where the ligand affinity weakened in the dimer state of M^pro^, the binding energy difference was < 1 kcal/mol that can be considered as insignificant.

**Figure 10:**
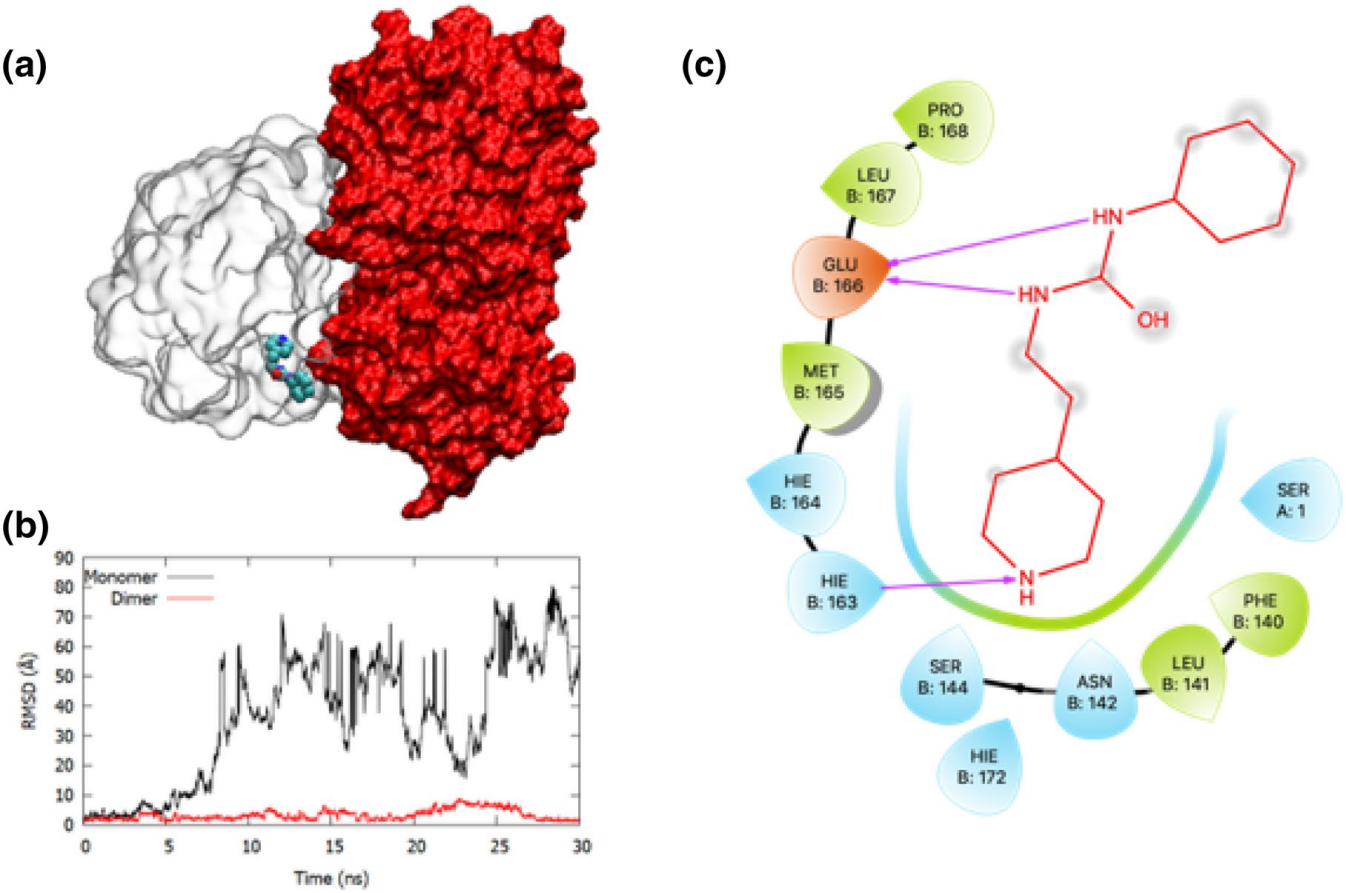
The binding of ligand from 5REH within a dimer model of M^pro^ (shown as surface representation) (**a**), along with the RMSD of the ligand when bound with the monomer and dimer (**b**) and the binding pose of the ligand within the dimer. The RMSD plots described that the ligand was unstable when bound to a monomer of M^pro^; however, it was more stable within a dimer condition. This is likely due to its favourable binding in the active site containing pocket where the ligand made stable H-bonds with HIS163 and GLU166 of monomer B thus occupying the lateral pocket that was extensively explored by other ligands part of our set (**c**). The N terminal of the second monomer, specifically SER 1, interacted in a hydrophobic manner, which indeed helped in the stable ligand interactions with M^pro^. In the 2D interaction diagram, the hydrophobic residues are shown in green; the polar residues in torquise; the negatively charged residues in orange; and the positively charged residues are shown in blue. The hydrogen bonds are displayed as purple arrows; and the solvent exposed sites in the bound ligand are noted with grey circles over the chemical structure of the ligand shown in red lines. The dimer molecular graphic shown in (**a**) was generated using VMD 1.9.3^63^ (**a**) while the two-dimensional interaction diagram was developed using Maestro Schrodinger^86^ (**c**). The plot in (**c**) was generated using GNUplot (v5.2 patchlevel 8 http://www.gnuplot.info/).

Subsequently, we analyzed the complexes, in which the ligands were originally bound to the catalytic site (pocket 1) and subsequently egressed the monomer M^pro^ during MD. This set of complexes included the PDB structures of 5REH, 5RF2, 5RF3, 5RF8 and 5R7Z. Again, as can be seen in the supplementary table, ST7, the engagement of the second M^pro^ chain did not improve the stability and affinity of most of these ligands. For instance, in the PDB complexes of 5RF2 and 5RF3, the presence of the second M^pro^ chain did not improve the affinity of the bound ligands (1-azanylpropylideneazanium in 5RF2; and pyrimidin-5-amine in 5RF3), which is not surprising given their small sizes. However, the only exception to this is the ligand 1-cyclohexyl-3-(2-pyridin-4-ylethyl) urea (PDB: 5REH) in Fig. 10a, whose affinity towards a M^pro^ dimer increased by ~ 14 kcal/mol when compared to the monomer. The stability of the ligand with the dimer over the monomer is described by the ligand RMSD fluctuation in both states (in Fig. 10b). Analyzing the MD trajectory of the ligand-bound dimer complex, we noticed that the binding of N-finger of the second monomer against the first monomer of M^pro^ closed the binding site, thereby, pushing the ligand further into the binding site. The closure of the catalytic pocket by the N-finger from the second M^pro^ chain pushed the pyridine ring of the ligand further into the lateral pocket to establish the crucial H-bond interactions with HIS163 (in Fig. 10c). Further, the amine groups in the ligand made stronger H-bonds with GLU166 (Fig. 10c) that helped to improve the stability and affinity of this complex.

Finally, the ligands that benefitted the most by the presence of the M^pro^ dimer were the ligands that bound at the dimer interface such as 1-(4-fluoro-2-methylphenyl)methanesulfonamide (5RGQ), 1-methyl-*N*-{[(2S)-oxolan-2-yl]methyl}-1H-pyrazole-3-carboxamide (5RFA), and [1-(pyridin-2-yl)cyclopentyl]methanol] (5RF0). While the methanesulfonamide ligand (in 5RGQ) remained stable even within the monomer structure, this was not the case with the other two ligands. Nevertheless, the presence of a dimer environment assisted in the stability of these ligands and improved their binding affinity with M^pro^ significantly. For example, the carboxamide-based ligand (in 5RFA) was bound at the interface of two M^pro^ monomers (Supplementary figure, SFig. 26a) and remained stable throughout MD simulation, which was not true when it was bound with a monomer enzyme, as described by the ligand RMSDs (Supplementary figure, SFig. 26b). Within the dimer form, the ligand underwent some structural changes around ~ 10-ns during MD and remained in a much stable pose that was supported by the interactions of the ligand with the key residues such as MET6, PHE8, ILE152, ASP298 from one M^pro^ monomer, and predominantly SER123 from the other monomer (Supplementary figure, SFig. 26c). This pose is described by the 2D interaction diagram shown in Supplementary figure, SFig. 26d. In a similar nature, the methanol-based ligand (in 5RF0) also displayed an enhanced affinity with the dimer when compared to the monomer form (refer to supplementary table, ST7). The 3D structure of all the ligand-bound dimer complexes along with their RMSD fluctuations in monomer and dimer states of M^pro^ are provided in the supplementary figure, Supplementary figure, SFig. 27.

Since dimerization of M^pro^ is a crucial mechanism for the activity of the enzyme and hence the replication of SARS-CoV-2 enzyme, there is an increasing interest towards disrupting the dimerization of M^pro^ in potential therapeutics. Therefore, the molecular level interactions of the ligands at a specific pocket present at the dimer junction identified through earlier X-ray crystal screening efforts^14^ and currently studied through our efforts using MD and binding free energy analyses could be useful for developing new compounds or identifying known drugs with physicochemical complementarity to this site. Identifying such compounds will allow us to explore the widely acknowledged novel approach of targeting the protease activity in SARS-CoV-2.

## Conclusion and future prospects

The novel coronavirus pandemic caused by the SARS-CoV-2 virus has emerged as a huge challenge for the twenty-first century. In less than a year of the outbreak, this infection has already caused over 105 million cases and 2.3 million deaths worldwide. Absence of a potent therapy against this virus remains a serious concern. The M^pro^ enzyme in SARS-CoV-2 has been identified as an ideal drug target as it plays a vital role in regulating the replication and transcription of the virus. With consistent scientific efforts, several high-resolution 3D structures of ligand–M^pro^ complexes have already been reported in the PDB and this number is rapidly growing. Understanding the dynamic interactions of these ligands offers useful insights about the ligand–M^pro^ complexes.

In this work, we employed a wide array of computational methods to present a comprehensive picture about ligand–M^pro^ interactions at the atomic level. We identified and characterized the conformational dynamic properties of 8 different pockets in the apo M^pro^ that included the known catalytic pocket (pocket 1). Ligands were found bound to almost all of these identified sites, with the majority of them binding to the catalytic site. Through MD and stability analyses, we showed that the ligands interacting in the catalytic site of M^pro^ monomer in general remained stable, except for a few ligands that egressed the protein during MD. We highlighted four key components in the catalytic site of M^pro^ and identified that the binding of ligands with these elements impacted their affinity to M^pro^. Specifically, we revealed a lateral pocket, featuring HIS163 as a key residue, which played a critical role in enhancing the stability and the affinity between ligands and M^pro^. We revealed that, in the absence of a bound ligand, the steric water molecules occupied the lateral pocket to engage with HIS163. When a ligand bound at this site, the steric water moved away to facilitate the binding of ligands to HIS163, which eventually increased the affinity of ligands to M^pro^. In silico mutation of this HIS163 to alanine reduced the affinity of the ligand with M^pro^, thereby clarifying the importance of this residue in efficient ligand recognition by M^pro^. In vitro assessment of the effects of HIS163 mutation will be useful to confirm its role in ligand–M^pro^ interactions. Our results also summarized different moieties such as pyridine, phenol and isoquinoline groups that are able to make stable interactions with HIS163 and the lateral pocket. Therefore, it is proposed that designing new compounds or identifying existing small molecules that have similar structural groups to engage the lateral pocket can exhibit enhanced affinity, and thus inhibitory potency, against M^pro^. In addition, we have described several key molecular dynamic interactions of few ligands that bound at the dimer interface of M^pro^. Such molecular-level insights could be useful to gain some perspectives on druggable hotspots at the dimer interface and developing small molecules for targeting the dimerization of M^pro^, a potent therapeutic strategy against SARS-CoV-2 M^pro^.

## Supplementary Information

Supplementary Information.

## Data Availability

Any data related to this work might be available from the corresponding author.

## Abbreviations

M^pro^: Main protease
MD: Molecular dynamics
PDB: Protein data bank
MM-GBSA: Molecular mechanics generalized born surface area
RMSD: Root mean square deviation
H-bond: Hydrogen bond
RMSF: Root mean square fluctuation
PCA: Principal component analyses
