# Supplementary material for: Molecular dynamics and in silico mutagenesis on the reversible inhibitor-bound SARS-CoV-2 main protease complexes reveal the role of lateral pocket in enhancing the ligand affinity
Source: Sci Rep. 2021 Apr 1;11:7429. doi: 10.1038/s41598-021-86471-0 (PMC8016996; doi:10.1038/s41598-021-86471-0)

Molecular dynamics and in silico mutagenesis on the reversible inhibitor-bound SARS-CoV-2 Main Protease complexes reveal the role of lateral pocket in enhancing the ligand affinity

Supplementary Information

Ying Li Weng[1$], Shiv Rakesh Naik[1$], Nadia Dingelstad[1$], Miguel R. Lugo, Subha Kalyaanamoorthy[2], Aravindhan Ganesan[1*]

[1]ArGan's Lab, School of Pharmacy, Faculty of Science, University of Waterloo, Ontario, Canada.
[2]Department of Chemistry, Faculty of Science, University of Waterloo, Ontario, Canada.
[$]Authors contributed equally.

*Corresponding author: aravindhan.ganesan@uwaterloo.ca

ST. 1: Following contains the list of PDB codes and the chemical structures of co-crystalized ligands for all the 62 reversible ligand-Mpro complexes studied in this work.

Index	PDB	Ligand	Index	PDB	Ligand
**1**	5R7Y	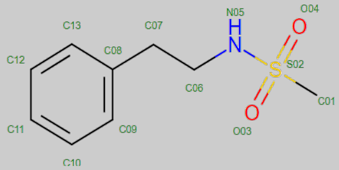	32	5RF5	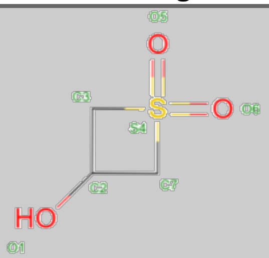
**2**	5R7Z	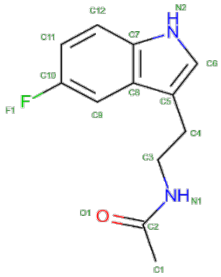	33	5RF6	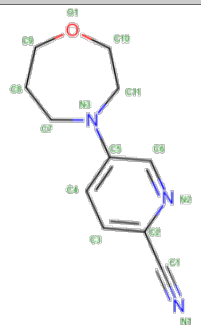
**3**	5R80	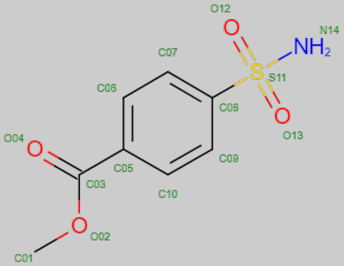	**34**	5RF7	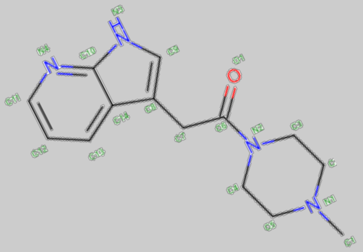
**4**	5R81	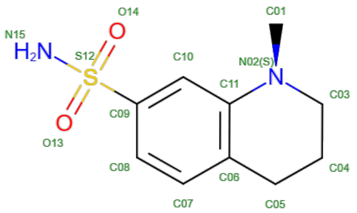	**35**	5RF8	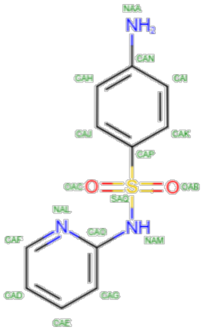

**5**	5R82	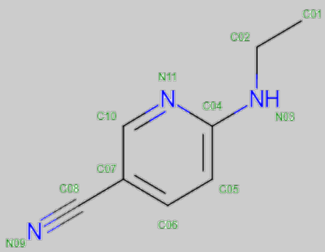	**36**	5RF9	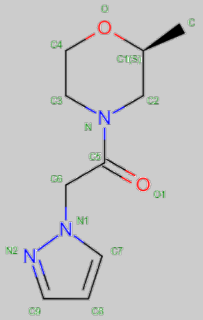
**6**	5R83	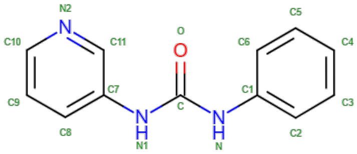	**37**	5RFA	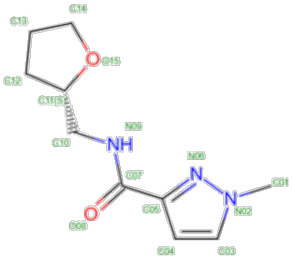
**7**	5R84	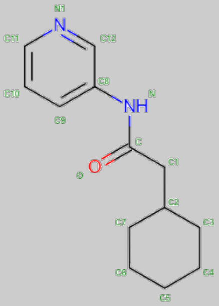	**38**	5RFB	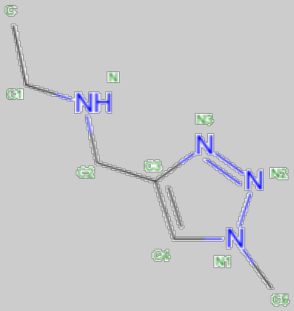
**8**	5RE4	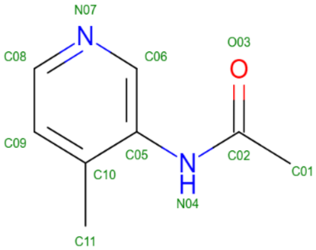	**39**	5RFC	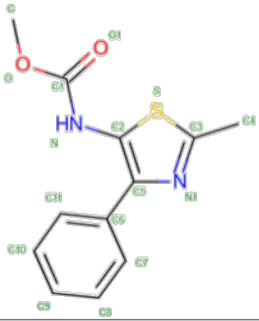
**9**	5RE5	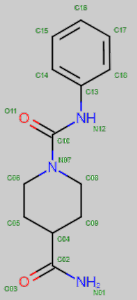	**40**	5RFD	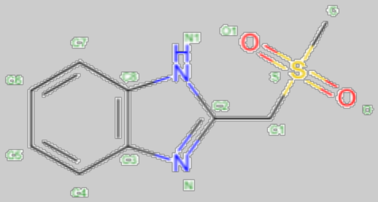

**10**	5RE6	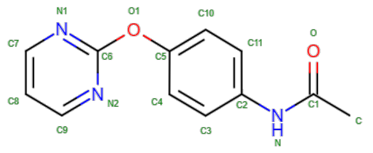	**41**	5RFE	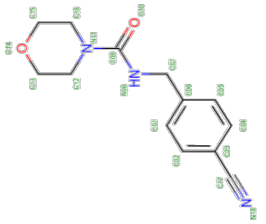
**11**	5RE7	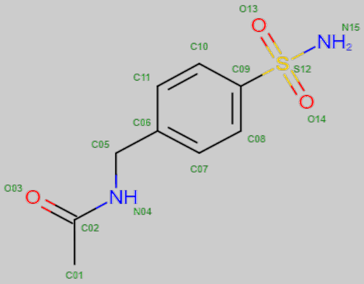	**42**	6W63	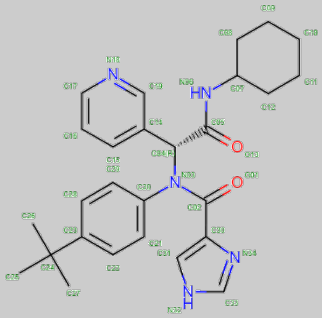
**12**	5RE8	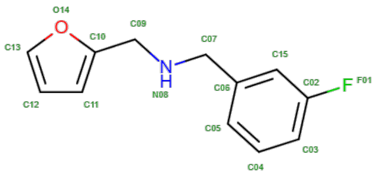	**43**	5RGG	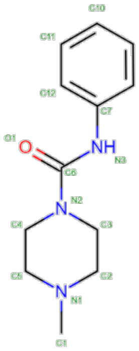
**13**	5RE9	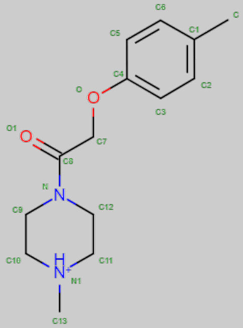	**44**	5RGQ	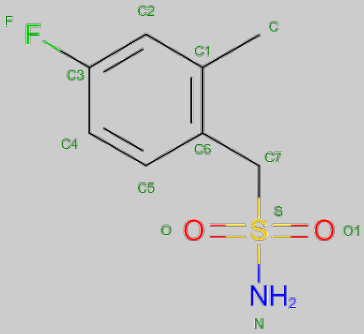
**14**	5REA	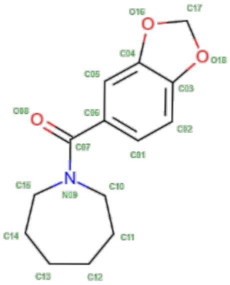	**45**	5RG1	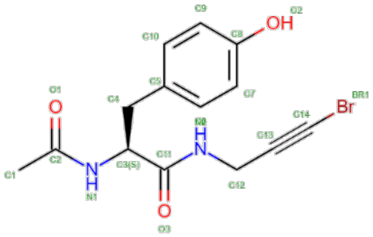

**15**	5REB	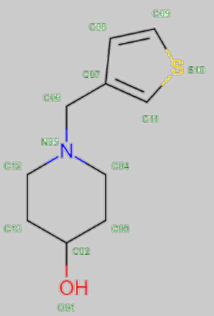	**46**	5RGH	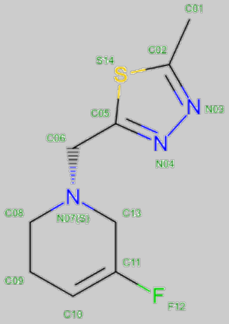
**¶¢16**	5REC	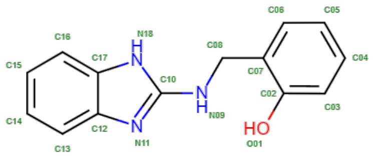	**47**	5RGI	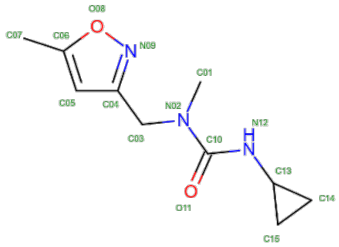
**17**	5RED	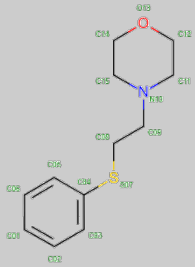	**48**	5RGJ	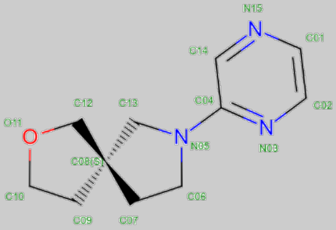
**18**	5REE	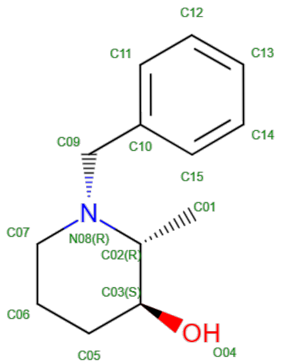	**49**	5RGK	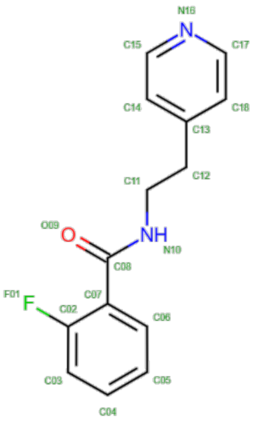
**19**	5REF	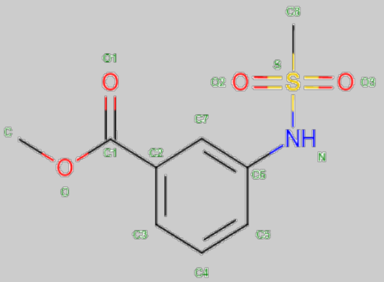	**50**	5RGR C1	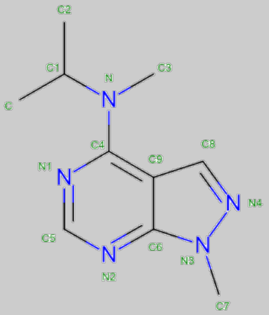

**20**	5REG	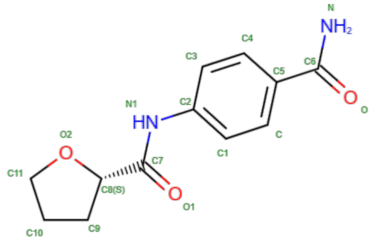	**51**	5RGR C2	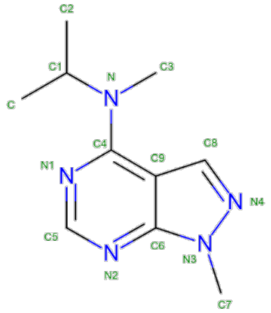
**21**	5REH	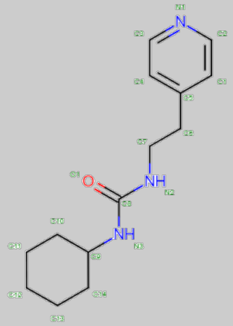	**52**	5RGS	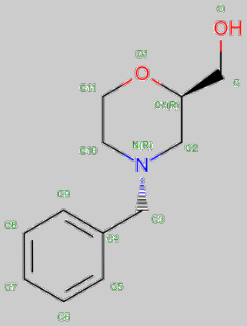
**22**	5REI	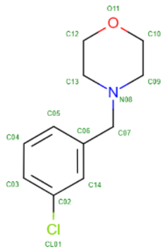	**53**	5RGY	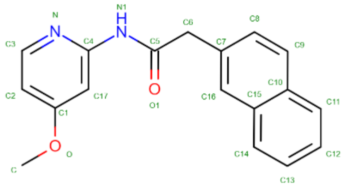
**23**	5RGZ	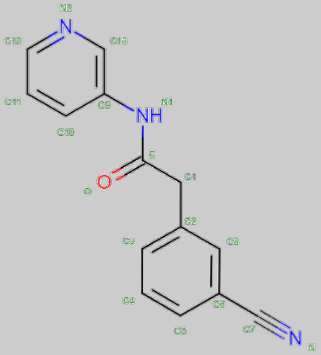	**54**	5RGX	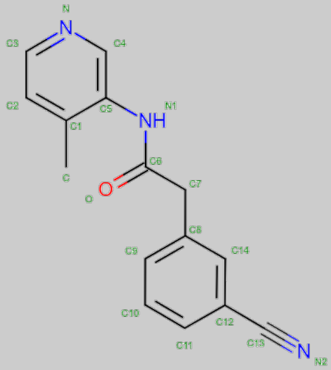

**24**	5RH4	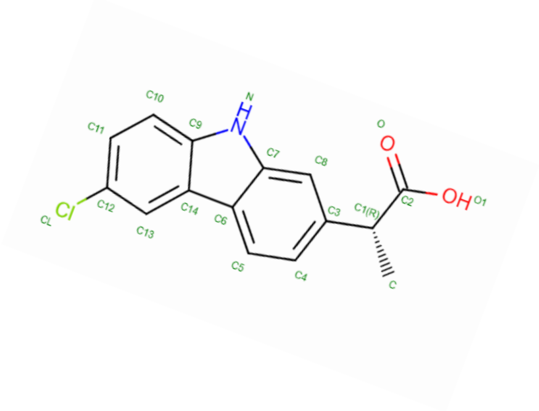	**55**	5RH0	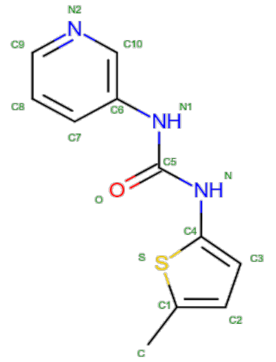
**25**	5RHD	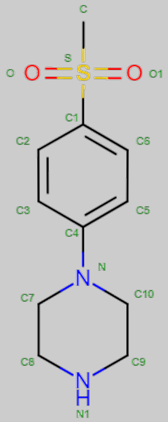	**56**	5RH2	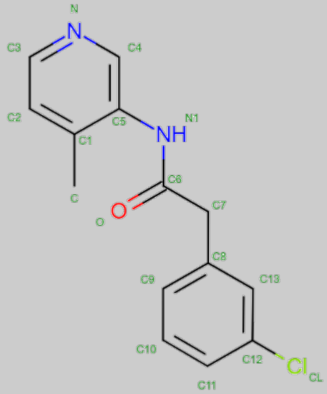
**26**	5REZ	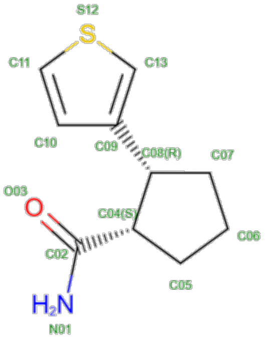	**57**	5RH1	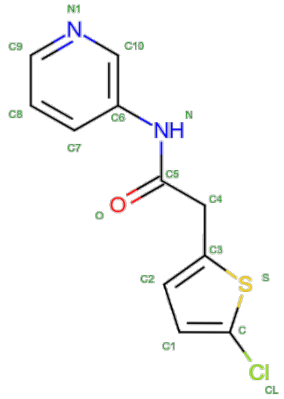
**27**	5RF0	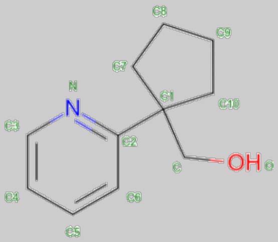	**58**	6YVF	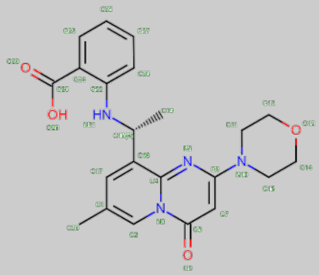

**28**	5RF1	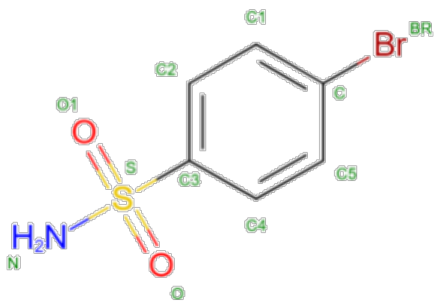	**59**	5RGV	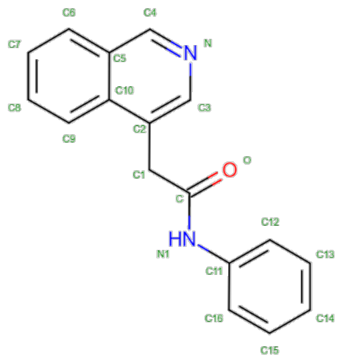
**29**	5RF2	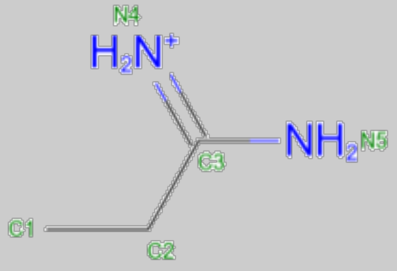	**60**	5RGW	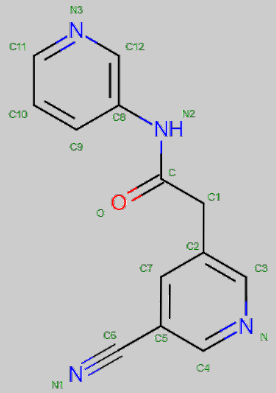
**30**	5RF3	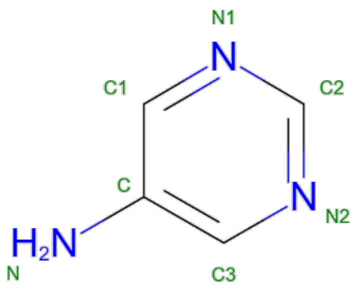	**61**	5RH3	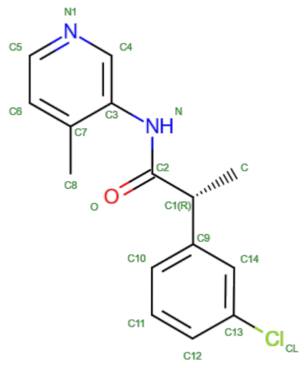
**31**	5RF4	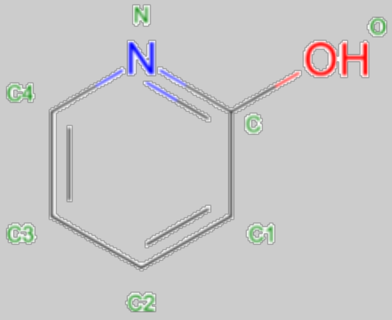	**62**	5RH8	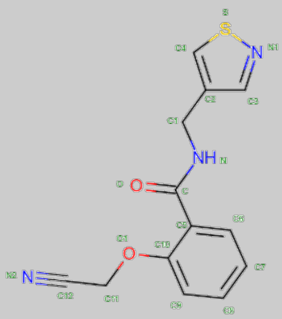

ST. 2: Apo pocket characterizations. More details on the pockets identified in the apo protein MD trajectory. Each were characterized by pocket name, residue composition, PDB ID's of ligands bound in their crystal structure positions, and pocket volume.

Pocket Name	Residues	PDB ID's in crystal structure	Volume
1	PHE140 LEU141 ASN142 GLY143 SER144 CYS145 HIS163 HIS164 MET165 GLU166 HIS172 VAL186 ASP187 ARG188 GLN189 GLN192	All the other PDB codes (from ST1) not listed below	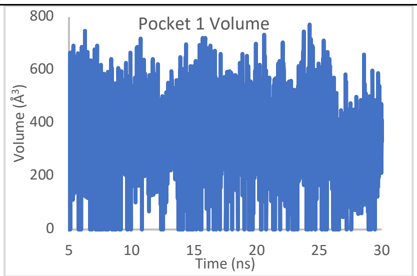
2	PHE3 ARG4 LYS5 MET6 PHE8 THR111 GLN127 PHE291 THR292 PHE294 ASP295 VAL296 ARG298 GLN299 GLY302 THR304	5RFA<br>5RGQ	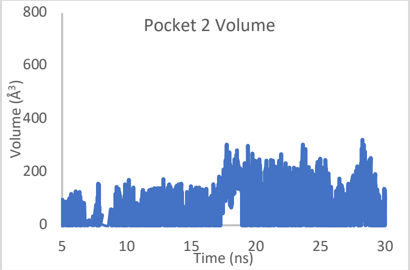
3	THR198 MET235 ASN238 TYR239 GLU240 PRO241	5REC<br>5RGS<br>5REE	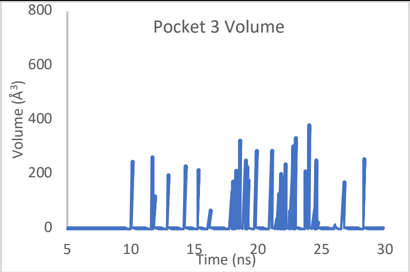
4	ASP34 VAL35 VAL36 TYR37 GLY79 HIS80 SER81 LYS88 LEU89 LYS90	5RFC<br>5RH4<br>5RE6<br>5RE5<br>5RGG<br>5RFB<br>6YVF	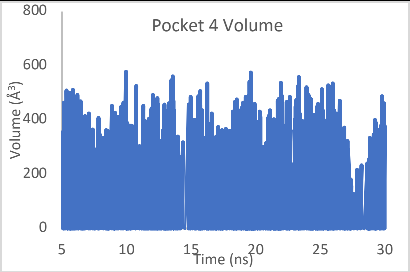
5	PHE103 VAL104 ARG105 PHE159 CYS160 TYR161 THR175 ASN176 LEU177 GLU178 ASN180 TYR182	5REI<br>5RED<br>5RF5<br>5RGR	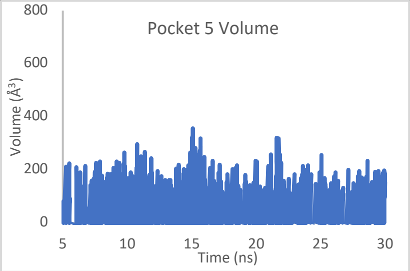

6	PHE8 PRO9 SER10 GLY11 LYS12 VAL13 GLU14 GLY15 CYS16 MET17 LEU30 LEU32 ASP33 ASP34 ILE78 LYS90 VAL91 ASP92 THR93 ALA94 ASN95 PRO96 LYS97 THR98 PRO99 LYS100 TYR101 LEU115 VAL148 PHE150 ASN151 ILE152 ASP155 CYS156 VAL157 PHE159	5RF4<br>5RFD<br>5RE8<br>5RF9<br>5RGJ	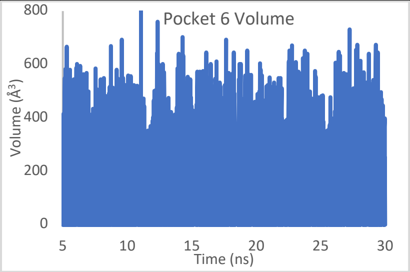
7	ARG105 ILE106 GLN107 PRO108 MET130 PHE134 PHE181 TYR182 GLY183 PRO184	5REG	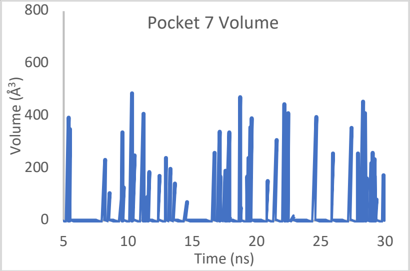
8*	LYS5 ILE106 GLN107 PRO108 GLY109 GLN110 THR111 PHE112 VAL114 LEU115 ALA116 TYR118 SER123 GLY124 VAL125 TYR126 GLN127 CYS128 ALA129 MET130 ARG131 PRO132 ASN133 PHE134 THR135 ILE136 LYS137 GLY138 SER139 PHE140 SER147 VAL148 GLY149 CYS160 TYR161 MET162 HIS163 LEU167 PRO168 THR169 GLY170 VAL171 HIS172 TYR182 ALA193 ALA194 GLY195 THR196 ASP197 THR198 THR199 ILE200 THR201 VAL202 ASN203 VAL204 LEU205 TYR237 ASN238 TYR239 GLU240 PRO241 LEU242 THR243 ASP245 HIS246 ILE249 LEU250 LEU287 GLU288 ASP289 GLU290 PHE291 THR292 PRO293 PHE294 ASP295	5RF0	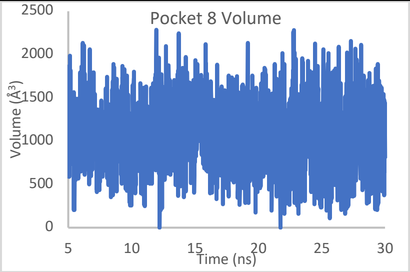

***The figure here depicts residues forming pocket 8 in yellow surface representation, red and blue represents monomer A and B respectively.**

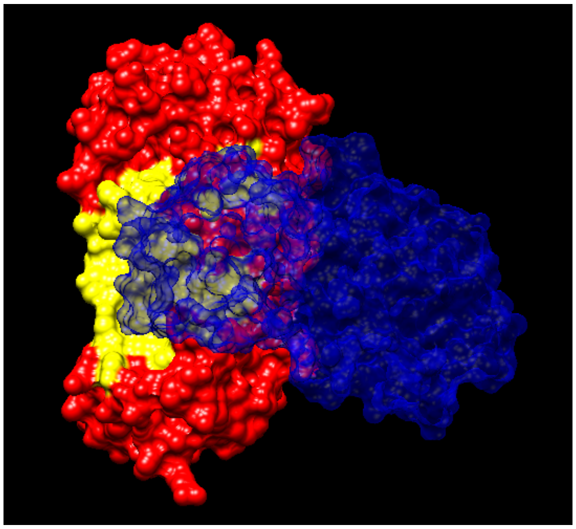

ST. 3: Below presented are all the 62 reversible ligands according to their crystal structure complexes, presented along with their MM-GBSA Binding Free Energy and averaged Protein Backbone RMSD and Unaligned Ligand RMSD. The first section of the table presents all the ligands in the active site/Pocket1 and the section thereafter the ligands are grouped according to their binding pocket.

Index	PDB	MMGBSA (kcal/mol)	Standard Deviation	Protein Average RMSD(Å)	Standard Deviation	Ligand Average RMSD(Å)	Standard Deviation	Pocket
**1**	**5R7Y**	**-18.120**	2.659	**1.917**	**0.338**	**4.350**	**0.871**	**1**
**2**	5R7Z	-7.526	6.067	2.082	0.381	10.366	6.734	1
**3**	5R80	-12.784	2.570	1.777	0.350	4.838	1.564	1
**4**	5R81	-18.376	2.926	1.499	0.191	3.676	1.165	1
**5**	5R82	-11.630	2.678	1.657	0.227	5.621	1.534	1
**6**	5R83	-15.371	2.424	1.857	0.370	2.307	0.735	1
**7**	5R84	-16.806	2.174	1.956	0.318	2.535	1.225	1
**8**	5RE4	-18.411	2.600	1.618	0.330	3.142	0.518	1
**13**	5RE9	-20.695	4.183	1.694	0.296	1.855	0.988	1
**15**	5REB	-14.650	3.325	2.129	0.416	7.587	3.175	1
**21**	5REH	0.003	0.016	1.744	0.328	35.867	21.568	1
**23**	5RGZ	-27.656	2.742	1.701	0.237	2.426	0.186	1
**25**	5RHD	-13.609	4.562	2.027	0.400	3.206	0.709	1
**26**	5REZ	-11.560	5.300	1.420	0.176	5.630	3.322	1
**29**	5RF2	-0.012	0.775	1.815	0.262	59.830	23.909	1
**30**	5RF3	-0.165	0.866	1.848	0.456	20.520	18.616	1
**33**	5RF6	-18.208	2.761	1.522	0.205	3.980	0.845	1
**34**	5RF7	-16.112	2.405	1.681	0.296	4.080	1.047	1
**35**	5RF8	-4.449	2.674	1.443	0.205	7.640	6.505	1
**41**	5RFE	-21.937	2.566	1.828	0.336	6.030	0.830	1
**42**	6W63	-34.356	3.592	1.756	0.372	2.100	0.524	1
**45**	5RG1	-36.334	4.194	1.506	0.298	1.204	0.376	1
**46**	5RGH	-12.129	3.161	1.672	0.195	3.571	1.199	1
**47**	5RGI	-23.811	2.648	1.783	0.380	1.507	0.403	1
**49**	5RGK	-14.525	2.593	1.673	0.287	4.006	2.590	1
**53**	5RGY	-19.166	3.041	1.810	0.336	3.248	0.852	1
**54**	5RGX	-30.026	2.886	1.627	0.190	2.424	0.780	1
**55**	5RH0	-22.245	2.348	1.672	0.269	1.550	0.530	1
**56**	5RH2	-24.270	2.464	1.683	0.402	1.894	0.660	1
**57**	5RH1	-25.936	3.584	1.485	0.257	4.530	2.266	1
**59**	5RGV	-22.649	2.317	1.798	0.266	2.113	0.480	1
**60**	5RGW	-22.799	2.768	2.034	0.422	1.564	0.556	1
**61**	5RH3	-24.488	2.635	1.556	0.221	1.260	0.425	1
**62**	5RH8	-22.146	2.454	1.545	0.267	3.984	0.732	1

Ligands initially bound in pockets other than pocket 1 are grouped below according to their initial site of binding.

**44**	5RGQ	-18.302	4.235	1.712	0.369	2.289	0.461	2
**37**	5RFA	-10.801	4.046	1.413	0.260	30.380	9.115	2
**16**	5REC	-17.564	2.752	1.852	0.278	3.644	0.731	3
**18**	5REE	-2.028	2.977	1.724	0.284	36.141	12.469	3
**52**	5RGS	-14.791	2.374	1.887	0.303	5.919	0.627	3
**9**	**5RE5**	**-16.203**	3.437	2.844	0.406	31.103	18.157	4
**10**	**5RE6**	**-12.056**	2.407	1.668	0.216	38.135	24.649	4
**38**	**5RFB**	**-6.844**	6.927	1.914	0.595	38.700	22.077	4
**39**	5RFC	-10.626	2.295	1.860	0.281	6.050	2.083	4
**43**	**5RGG**	**-10.025**	3.983	1.371	0.153	21.941	16.319	4
**24**	**5RH4**	**-17.503**	3.620	1.921	0.284	3.481	0.734	4
**58**	**6YVF**	**-15.349**	4.336	1.856	0.213	3.440	1.159	4
**22**	5REI	-7.799	3.274	1.478	0.214	22.509	13.850	5
**17**	5RED	-1.431	2.790	1.565	0.249	49.817	16.664	5
**32**	5RF5	0.345	1.149	2.247	0.436	35.110	15.835	5
**50**	5RGR	-3.080	4.495	1.899	0.338	21.967	15.395	5
**51**	5RGR	-9.098	2.211	1.801	0.217	24.340	21.539	5
**58**	6YVF	-15.349	4.336	1.856	0.213	3.440	1.159	5
**31**	5RF4	-3.643	3.040	1.764	0.333	63.180	18.851	6
**12**	5RE8	-0.109	0.581	1.673	0.318	47.159	14.972	6
**36**	5RF9	-8.414	2.367	1.903	0.370	24.870	8.164	6
**40**	5RFD	-1.518	2.375	1.359	0.243	40.740	13.493	6
**48**	5RGJ	-6.613	2.960	1.812	0.402	45.739	10.069	6
**20**	5REG	-18.222	2.241	2.866	0.952	2.931	1.130	7
**27**	5RF0	-2.051	3.178	1.616	0.284	31.213	15.480	8
**11**	5RE7	-0.616	1.843	2.148	0.296	40.999	21.265	Unique pocket
**14**	5REA	-16.038	3.007	2.273	0.701	6.311	2.029	Unique pocket
**28**	5RF1	-9.191	2.395	1.934	0.321	3.520	1.273	Unique pocket

ST. 4: The average RMSD of protein and ligand in the selected ligand-M^{pro} complexes from the 100 ns long MD simulations. The comparison of the binding free energy values from the 30 ns and 100 ns trajectories, along with The $\Delta\Delta G_{(bind)}$ values, are also provided.

PDB	Protein		Ligand		MMGBSA Free Energy (ΔG) 80ns	Standard Deviation	MMGBSA Free Energy (ΔG) 30ns	Standard Deviation	Absolute Free Energy Difference (ΔΔG)
	100ns Average RMSD(Å)	Standard Deviation	100ns Average RMSD(Å)	Standard Deviation					
**5RG1**	1.620	0.313	2.321	1.944	-30.892	7.430	-36.334	4.194	5.442
**6W63**	2.368	0.522	2.661	0.590	-36.568	3.912	-34.356	3.592	2.211
**5RGX**	2.010	0.556	1.955	0.668	-26.004	3.071	-30.026	2.886	4.022
**5RGZ**	2.077	0.437	2.473	0.203	-25.827	3.092	-27.656	2.742	1.829
**5RH1**	1.752	0.313	4.288	1.449	-25.713	2.858	-25.936	3.584	0.222
**5RH3**	1.884	0.409	2.661	2.205	-22.448	4.171	-24.488	2.635	2.040
**5RH2**	2.160	0.523	3.490	1.836	-19.430	3.732	-24.270	2.464	4.840
**5RGI**	2.047	0.430	1.820	0.487	-24.300	3.007	-23.811	2.648	0.489
**5RGW**	2.186	0.318	2.098	0.716	-19.835	2.899	-22.799	2.768	2.963
**5RGV**	2.119	0.331	2.277	0.564	-20.957	2.694	-22.649	2.317	1.692
**5RH0**	1.790	0.274	5.772	4.912	-15.958	5.327	-22.245	2.348	6.287
**5RH8**	1.794	0.353	4.996	1.034	-21.214	2.793	-22.146	2.454	0.933
**5RFE**	1.891	0.254	5.988	0.521	-23.958	2.530	-21.937	2.566	2.021
**5RE9**	1.900	0.279	24.023	17.455	-8.655	8.535	-20.695	4.183	12.040

ST. 5: Mutation analysis of HIS163 to ALA163. A mutation analysis was performed on five PDB complexes; 5RGZ, 5RF7, 6W63, 5RG1, and 5RGX. HIS163 was mutated to ALA163 in these complexes to demonstrate the importance of a key residue in the lateral pocket for ligand binding. Stable3 compares the initial wildtype (WT) MMGBSA vs. mutated MMGBSA, and HIS163 contribution (in kcal/mol) vs. ALA163 contribution (in kcal/mol). As seen in this table, PDB 5RGX demonstrated the highest MMGBSA difference of -8.868 kcal/mol and PDB 5RF7 demonstrated the least MMGBSA difference of -1.076 kcal/mol.

**Index**	**PDB**	**MMGBSA (WT)**	**MMGBSA (Mutant)**	**HIS163 contribution**	**ΔΔG (WT-mutant)**	**HIS163 contribution**	**ALA163 contribution**
**45**	5RG1	-36.334	-32.162	-2.340	4.172	-2.340	-0.080
**54**	5RGX	-30.026	-21.158	-3.680	8.868	-3.680	-0.760
**23**	5RGZ	-27.656	-22.073	-3.470	5.582	-3.470	-0.070
**34**	5RF7	-16.112	-15.040	-3.140	1.076	-3.140	0.660
**42**	6W63	-34.356	-29.017	-3.240	5.339	-3.240	0.800

ST. 6: Summary of MMGBSA-NWAT results from last 30ns production simulation, NWAT=0 is the energy scores without explicit water molecules and NWAT=6 represents the energy released by complex when there are 6 explicit water molecules in the M^{pro} receptor.

**Index**	**PDB**	**NWAT=0**	**NWAT=1**	**NWAT=2**	**NWAT=3**	**NWAT=4**	**NWAT=5**	**NWAT=6**	**Energy change**
**1**	5R7Y	-18.129	-18.375	-19.351	-20.156	-21.006	-22.059	-23.167	-5.039
**3**	5R80	-12.791	-13.415	-14.545	-15.718	-16.472	-17.083	-17.793	-5.002
**4**	5R81	-18.385	-19.720	-21.420	-22.530	-23.357	-24.111	-24.936	-6.551
**5**	5R82	-11.634	-11.395	-11.606	-11.926	-12.196	-12.532	-12.933	-1.299
**6**	5R83	-15.379	-17.100	-18.088	-18.533	-18.772	-18.979	-19.296	-3.918
**7**	5R84	-16.811	-17.934	-19.065	-20.017	-20.582	-20.853	-21.186	-4.375
**8**	5RE4	-18.419	-18.619	-18.476	-18.387	-18.495	-18.836	-19.281	-0.862
**13**	5RE9	-20.719	-20.065	-19.515	-19.160	-19.029	-19.181	-19.529	1.189
**15**	5REB	-14.654	-16.580	-18.108	-18.942	-19.342	-19.632	-20.103	-5.450
**23**	5RGZ	-27.667	-29.580	-30.099	-30.553	-31.122	-31.820	-32.585	-4.919
**25**	5RHD	-13.633	-17.762	-19.219	-19.568	-20.153	-21.051	-22.085	-8.452
**33**	5RF6	-18.213	-18.308	-18.369	-18.346	-18.443	-18.739	-19.203	-0.989
**34**	5RF7	-16.124	-17.544	-18.116	-17.817	-17.577	-17.522	-17.651	-1.527
**41**	5RFE	-21.943	-22.940	-23.938	-24.248	-24.342	-24.505	-24.820	-2.878
**42**	6W63	-34.104	-34.750	-35.532	-36.208	-36.729	-37.055	-37.418	-3.314
**45**	5RG1	-36.346	-37.724	-39.237	-40.123	-40.659	-41.207	-41.841	-5.495
**46**	5RGH	-12.132	-11.946	-12.123	-12.428	-12.580	-12.811	-12.996	-0.865
**47**	5RGI	-23.819	-22.944	-22.532	-22.372	-22.360	-22.541	-22.870	0.949
**49**	5RGK	-14.531	-15.957	-16.274	-16.121	-15.938	-15.807	-15.860	-1.329
**53**	5RGY	-19.173	-20.730	-21.763	-22.155	-22.056	-21.748	-21.498	-2.325
**54**	5RGX	-30.039	-29.344	-29.054	-29.073	-29.326	-29.821	-30.415	-0.376
**55**	5RH0	-22.251	-24.294	-25.926	-26.155	-26.257	-26.632	-27.203	-4.952
**56**	5RH2	-24.278	-25.949	-26.778	-26.743	-26.791	-26.965	-27.347	-3.068
**57**	5RH1	-25.945	-26.808	-26.608	-26.530	-26.712	-27.041	-27.435	-1.490
**59**	5RGV	-22.657	-24.224	-25.081	-25.219	-25.320	-25.549	-25.874	-3.217
**60**	5RGW	-22.807	-24.344	-25.240	-25.586	-25.793	-26.197	-26.689	-3.882
**62**	5RH8	-22.152	-22.996	-23.574	-23.667	-23.785	-24.163	-24.659	-2.507

ST. 7: The following table summarizes all they ligands in PDB complexes that were run in both the monomeric and dimeric simulations alongwith their calculated MMGBSA binding affinity in both cases as well as the residues that had a significant impact on the binding energy in each case extracted from the pairwise decomposition calculations. Ligands in 5RFA,5RGQ and 5RF0 were present at the dimer interface and as indicated through the binding affinity changes, were stable already in the monomer(5RGQ) or became stable only when simulated in the dimer model (5RF0 and 5RFA). The rest of the compounds were found within proximity of the dimer interface and this includes the active site. While only 5REH(in the active site) was stabilised when simulated in the dimer, the rest of the ligands either had minimal impact from the dimer simulation or even a negative impact in the case of 5RE7.

PDB ID	Average (Monomer)	Std. Dev.	Average (Dimer)	Std. Dev.	Decomposed residues(monomer)	Decomposed residues (Dimer)
5rgq	-18.3015	4.23	-21.786	3.15	4 6 8 295 298 299	308 310 429 593 397 600 601
5rfa	-10.8007	4.05	-15.9308	2.49	Weak binding	123 310 454 456
5reh	0.0032	0.02	-14.4416	3.01	Weak binding	468 465 444 442
5rez	-11.56	5.3	-11.8501	4.02	Weak binding	Weak binding
5rf0	-2.0509	3.18	-10.9406	4.26	Weak binding	Stable
5r7z	-7.5255	6.07	-7.8501	4.51	Weak binding	Weak binding
5re7	-0.6158	1.84	-7.5187	3.46	Weak binding	153 294
5rf9	-8.414	2.37	-7.3238	5.46	Weak binding	Weak binding
5rgj	-6.6131	2.96	-6.1587	4.54	Weak binding	Weak binding
5re8	-0.1094	0.58	-3.5218	3.04	Weak binding	Weak binding
5rf1	-9.1914	2.39	-3.4376	2.91	Weak binding	Weak binding
5rf3	-0.1652	0.87	-2.5144	2.49	Weak binding	Weak binding
5rf2	-0.0124	0.78	-0.1581	1.03	Weak binding	Weak binding

SFig. 1 Sequence alignment between the AA sequence for SARS CoV-2 M^{pro} and SARS CoV M^{pro}. SARS-CoV-2 M^{pro} shares 96% protein sequence similarity with SARS-CoV-1 M^{pro}, as indicated by Clustal x2 (2.1) multiple sequence alignment. The 12 mutations are highlighted by dots below the sequence alignment.

```
CLUSTAL 2.1 multiple sequence alignment


pdb|6LU7|A      SGFRKMAFPSGKVEGCMVQVTCGTTTLNGLWLDDVVYCPRHVICTSEDMLNPNYEDLLIR 60
pdb|2C3S|A      SGFRKMAFPSGKVEGCMVQVTCGTTTLNGLWLDDTVYCPRHVICTAEDMLNPNYEDLLIR 60
                **********************************.**********:**************

pdb|6LU7|A      KSNHNFLVQAGNVQLRVIGHSMQNCVLKLKVDTANPKTPKYKFVRIQPGQTFSVLACYNG 120
pdb|2C3S|A      KSNHSFLVQAGNVQLRVIGHSMQNCLLRLKVDTSNPKTPKYKFVRIQPGQTFSVLACYNG 120
                ****.********************:*:*****:**************************

pdb|6LU7|A      SPSGVYQCAMRPNFTIKGSFLNGSCGSVGFNIDYDCVSFCYMHHMELPTGVHAGTDLEGN 180
pdb|2C3S|A      SPSGVYQCAMRPNHTIKGSFLNGSCGSVGFNIDYDCVSFCYMHHMELPTGVHAGTDLEGK 180
                *************.*********************************************:

pdb|6LU7|A      FYGPFVDRQTAQAAGTDTTITVNVLAWLYAAVINGDRWFLNRFTTTLNDFNLVAMKYNYE 240
pdb|2C3S|A      FYGPFVDRQTAQAAGTDTTITLNVLAWLYAAVINGDRWFLNRFTTTLNDFNLVAMKYNYE 240
                *********************:**************************************

pdb|6LU7|A      PLTQDHVDILGPLSAQTGIAVLDMCASLKELLQNGMNGRTILGSALLEDEFTPFDVVRQC 300
pdb|2C3S|A      PLTQDHVDILGPLSAQTGIAVLDMCAALKELLQNGMNGRTILGSTILEDEFTPFDVVRQC 300
                **************************:*****************::**************

pdb|6LU7|A      SGVTFQ 306
pdb|2C3S|A      SGVTFQ 306
                ******
```

SFig. 2 – RMSD for 6M2Q apo structure, organized in domains. Images/graphs were created using GNUplot (v5.2 patchlevel 8 http://www.gnuplot.info/).

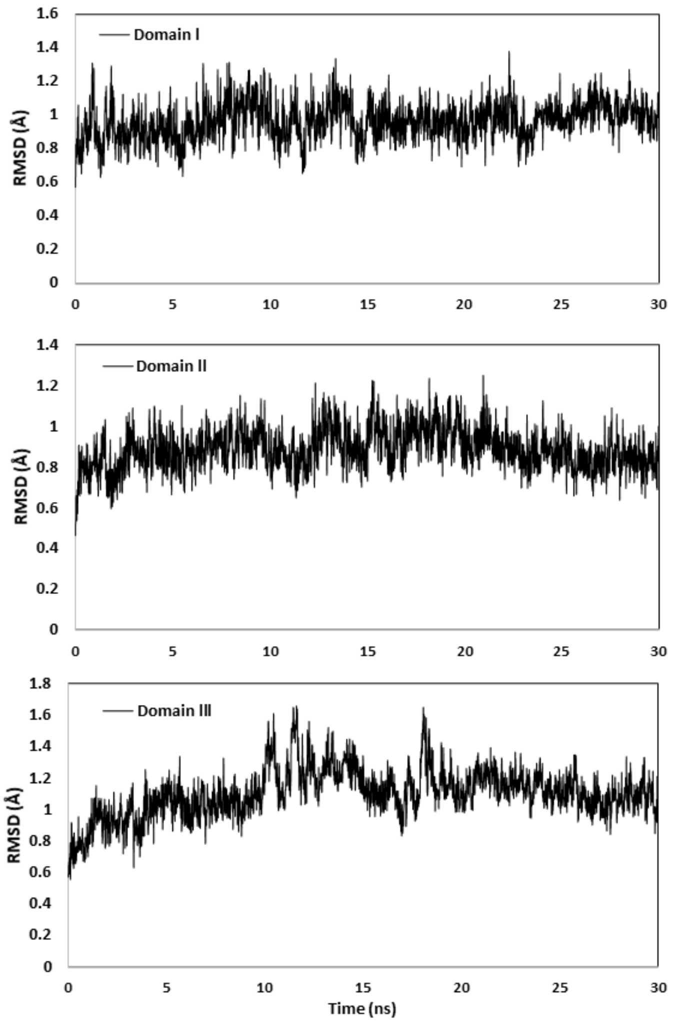

SFig. 3 Different interactions like hydrogen bonds within protein residues (*), salt bridges (**) and water molecules (***) are examined. Details for each interaction are shown in SFig. 3. Images/graphs were created using VMD 1.9.3[1].

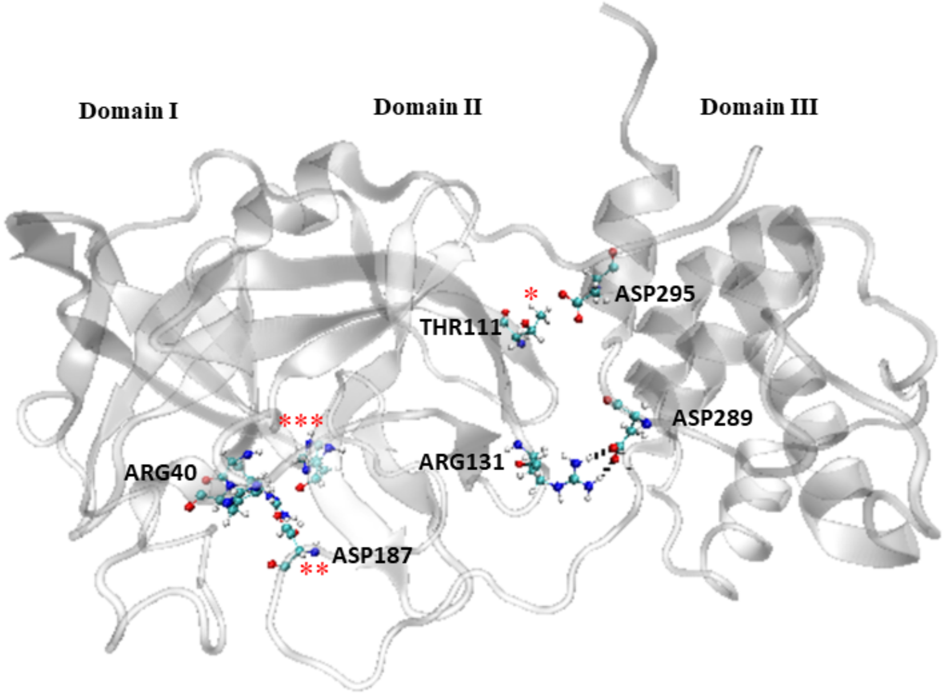

SFig. 4 – Interactions in apo 6M2Q, hydrogen bonds between THR111, ASP295 (a), and salt bridges between ARG131, ASP189(b), and between ARG40, ASP289 (c). Images/graphs were created using VMD 1.9.3[1], Microsoft Excel 365 (https://www.office.com/).

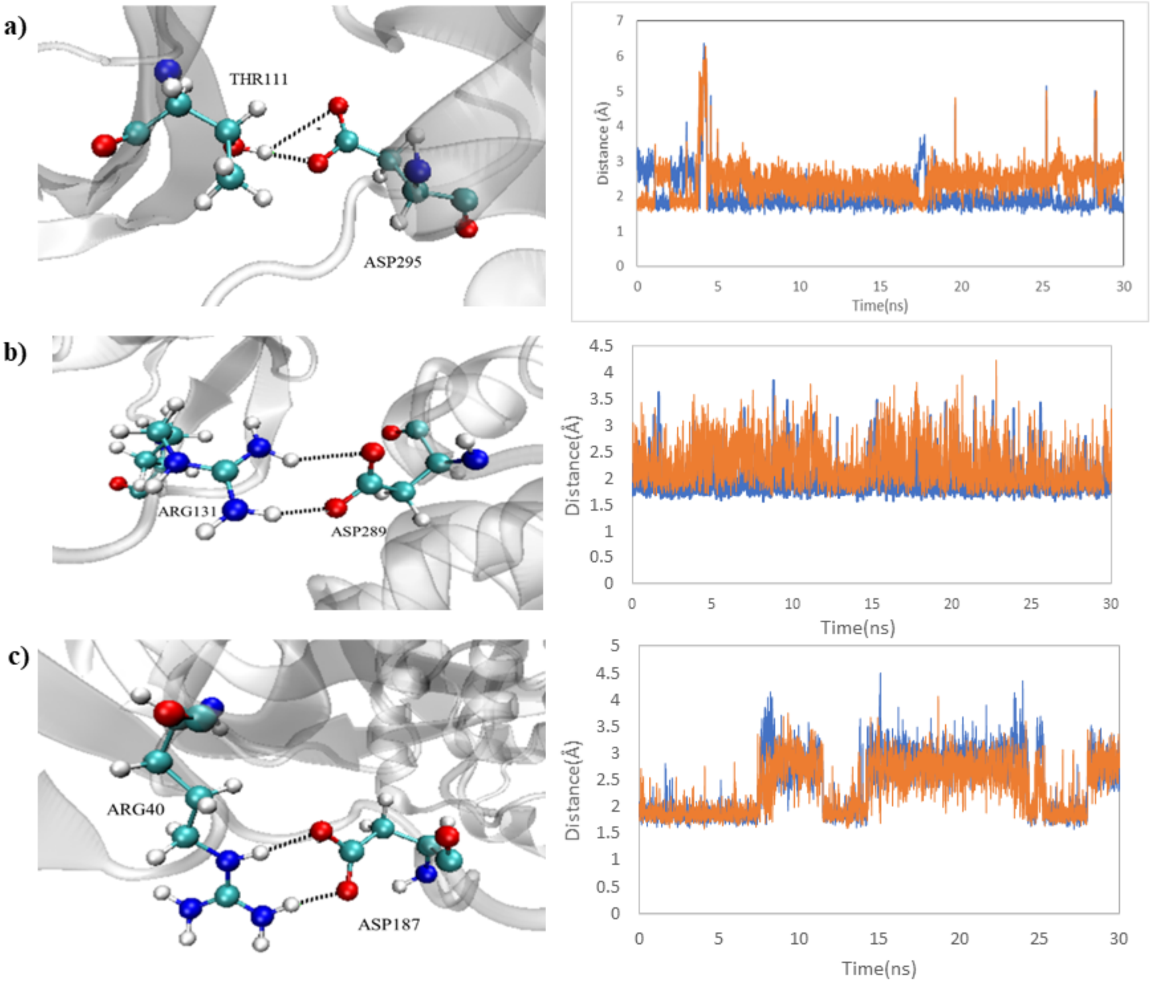

SFig. 5: Average RMSD values for the backbone of the proteins (a) and the ligands (b-c) in the ligand-Mpro complexes studied in this work. The average RMSDs of the proteins were mostly less than 3 Å; whereas the some of the ligand RMSDs exhibited an excessively large RMSD values, which indicated the unbinding of those ligand from the protein during MD simulation. The average ligand RMSDs after removing those ligand with RMSD values > 20 Å is shown in (c). Graphs were created using Microsoft Excel 365 (https://www.office.com/).

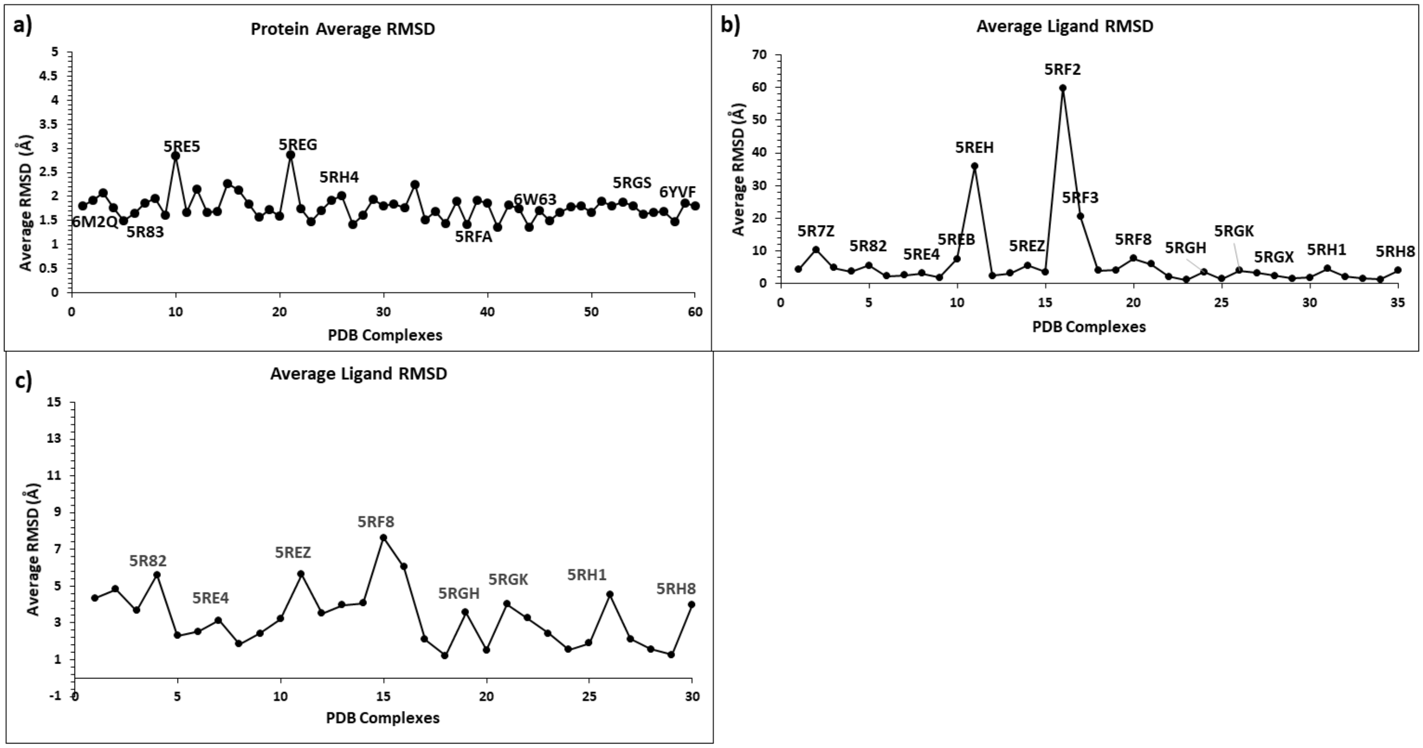

SFig. 6 Ligand (US7) pose of PDB ID 5RHD (a) and its RMSD plots (b). US7 represented in red and blue to show ligand position in beginning and end of production simulation (a). RMSD plots were provided to show stable protein backbone and blue ligand represents favorable pose throughout MD, as RMSD fluctuates around 3.5 Å for about 27 ns (b). Images/graphs were created using VMD 1.9.3[1](a), GNUplot (v5.2 patchlevel 8 http://www.gnuplot.info/) (b).

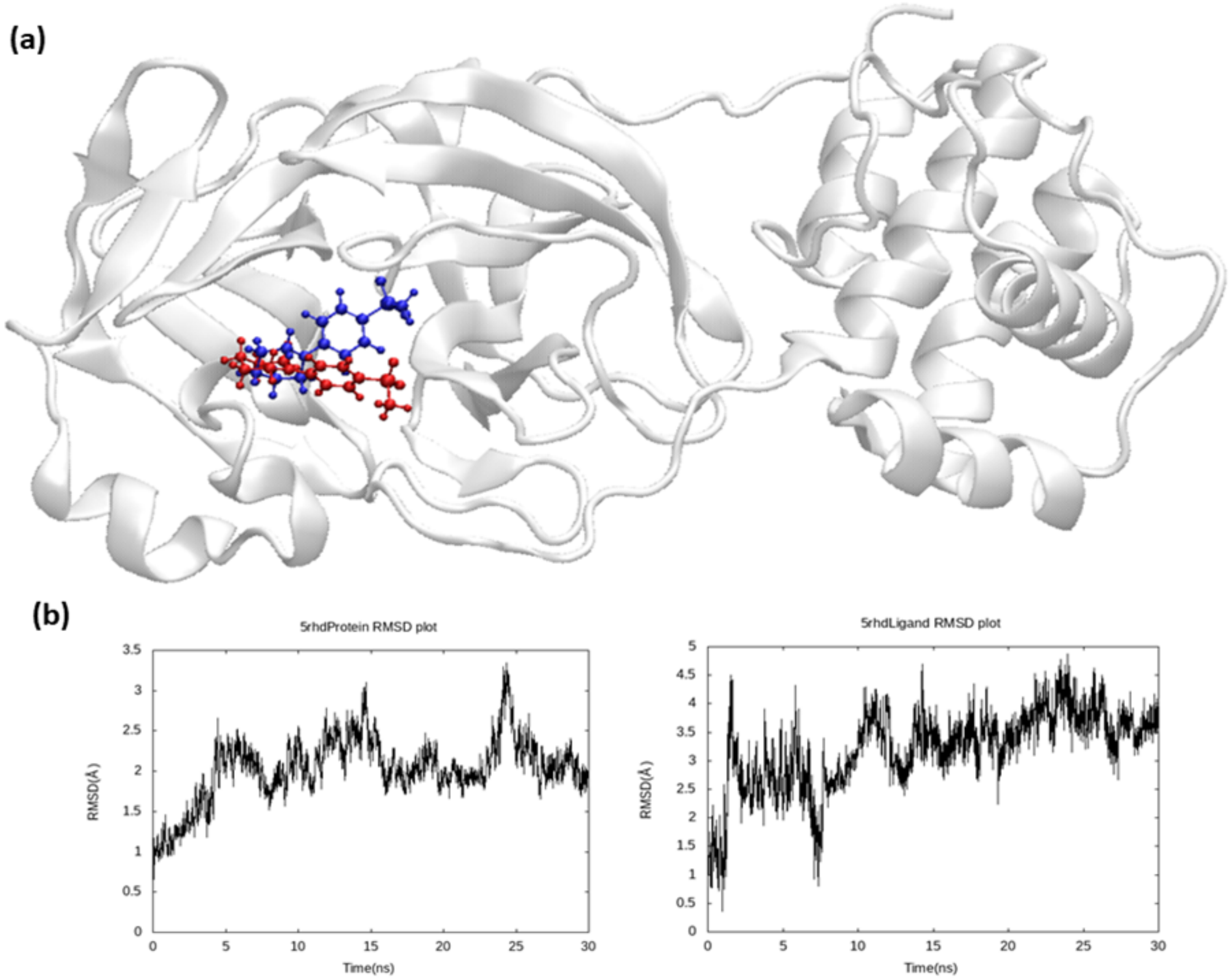

SFig. 7 The following graphs represent the average ligand RMSD of the compounds in Pockets 2-6 as observed in the 30ns simulation. Details of ligands in pockets 7,8 and unique pockets are outline in Stable 3 above. Graphs were created using Microsoft Excel 365 (https://www.office.com/).

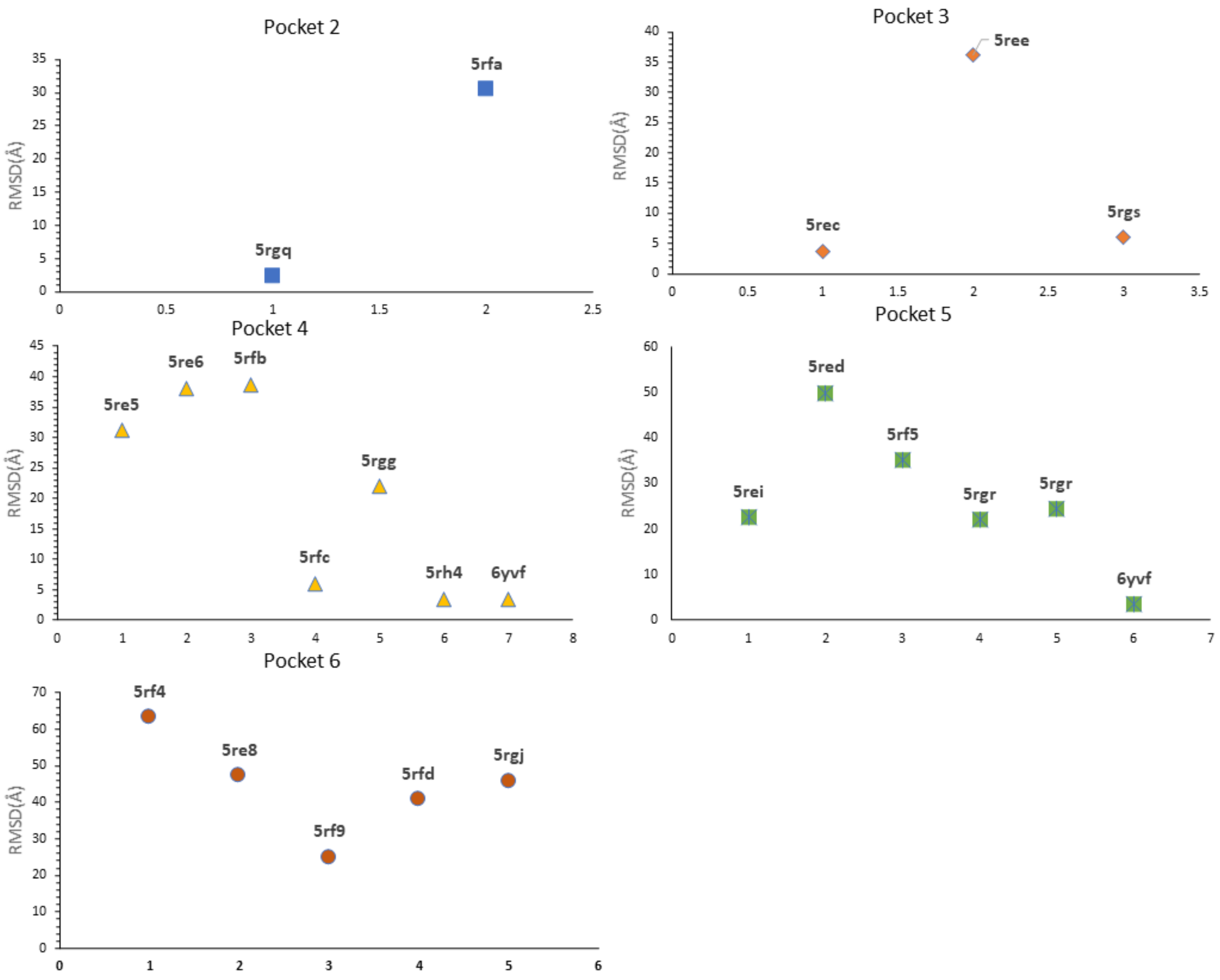

SFig. 8. Graph representing the MMGBSA binding affinities calculated in terms of free energy (kcal/mol)for 34 non-covalently bound ligans that are bound the the Main protease in an unstable manner and ligands bound to other sites other than the orthosteric site. Graphs were created using Microsoft Excel 365 (https://www.office.com/).

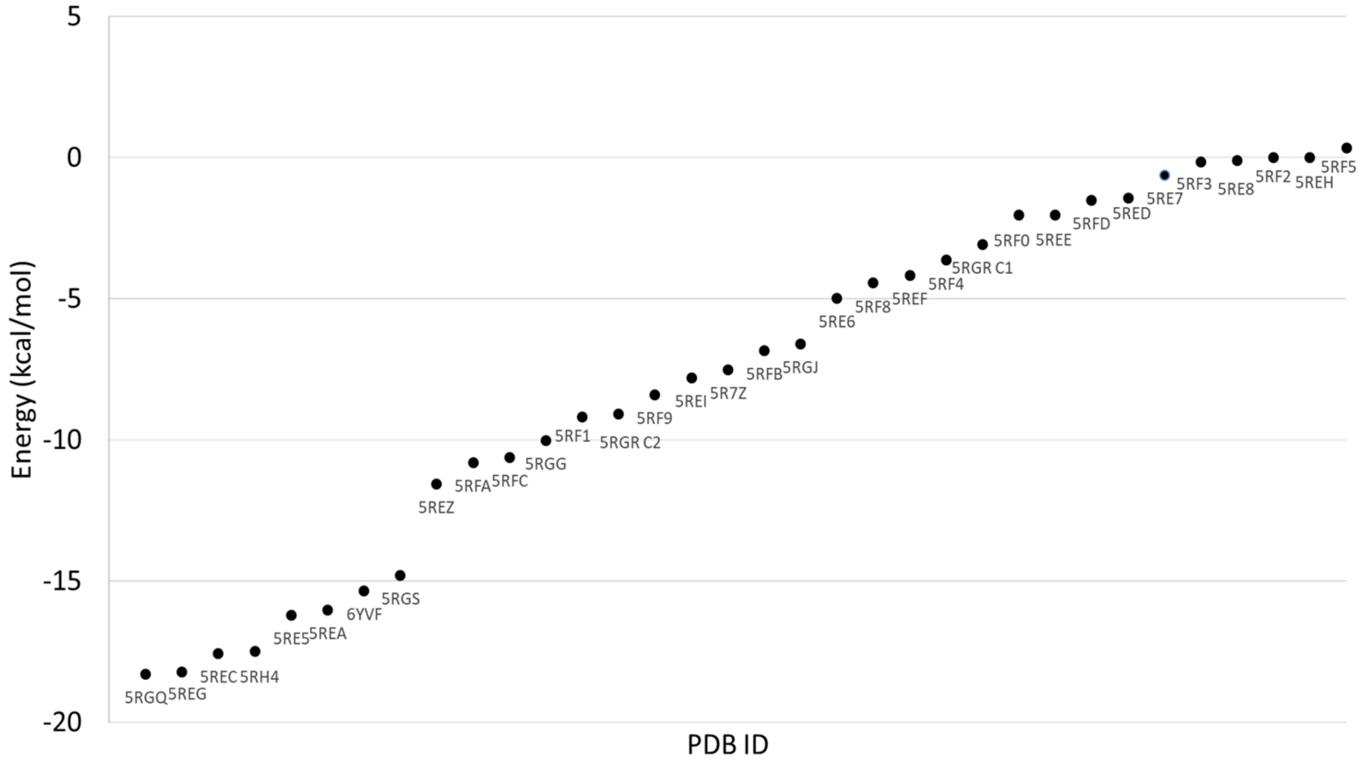

SFig. 9. Diagrams representing the MD behavior of ligand in complex 5RGH(b) (Binding affinity -12.1 kcal/mol) and some of the interacting residues (a). The different confirmations of the ligand shown as a function of time in (b) range from 0ns (red) to 29ns(blue) with white and pink showing intermediate poses, clearly highlighting the exhaustive motion around the active site while being anchored near the 180's loop through fluoride interactions with surrounding residues. Images were created using VMD 1.9.3[1].

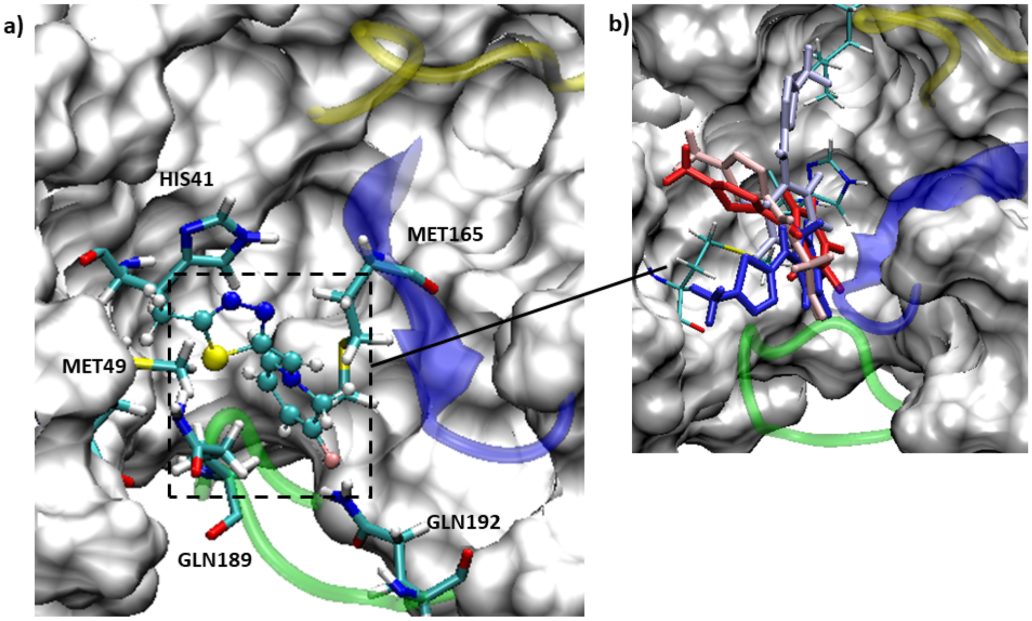

SFig. 10. Diagram showing the ligand in complex 5R82 with a binding affinity of -11.6 kcal/mol changing confirmations frequently in the binding site which are represented in the same diagram and coloured according to the timescale from 0ns (red) to 29ns (blue) and the colours in between. This fluctuating behavior explains why the ligand in 5R82 has relatively poor binding affinity. Images were created using VMD 1.9.3[1].

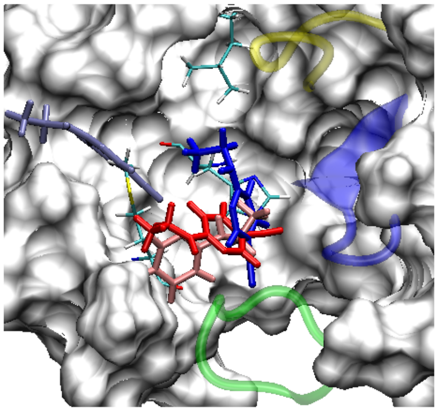

SFig. 11. Another high scoring ligand, in complex 5RH3 with a binding affinity of -22kcal/mol. The interaction diagram in (a) shows the key residues in stick representation which impact the overall binding affinity of the ligand as seen in the pairwise decomposition graph in (b). Stable hydrogen bonds with GLU166 and HIS163 shown in (c,d). These hydrogen bonds are clearly stable and therfore instrumental in stabilising this ligand in addition to the Van der Waals interactions with MET49, GLN189, MET165 and ASN 142 seen through the stick representations in (a) and through their energy impacts in(b). Images/graphs were created using VMD 1.9.3[1](a), GNUplot (v5.2 patchlevel 8 http://www.gnuplot.info/)(c,d), Microsoft Excel 365 (https://www.office.com/)(b).

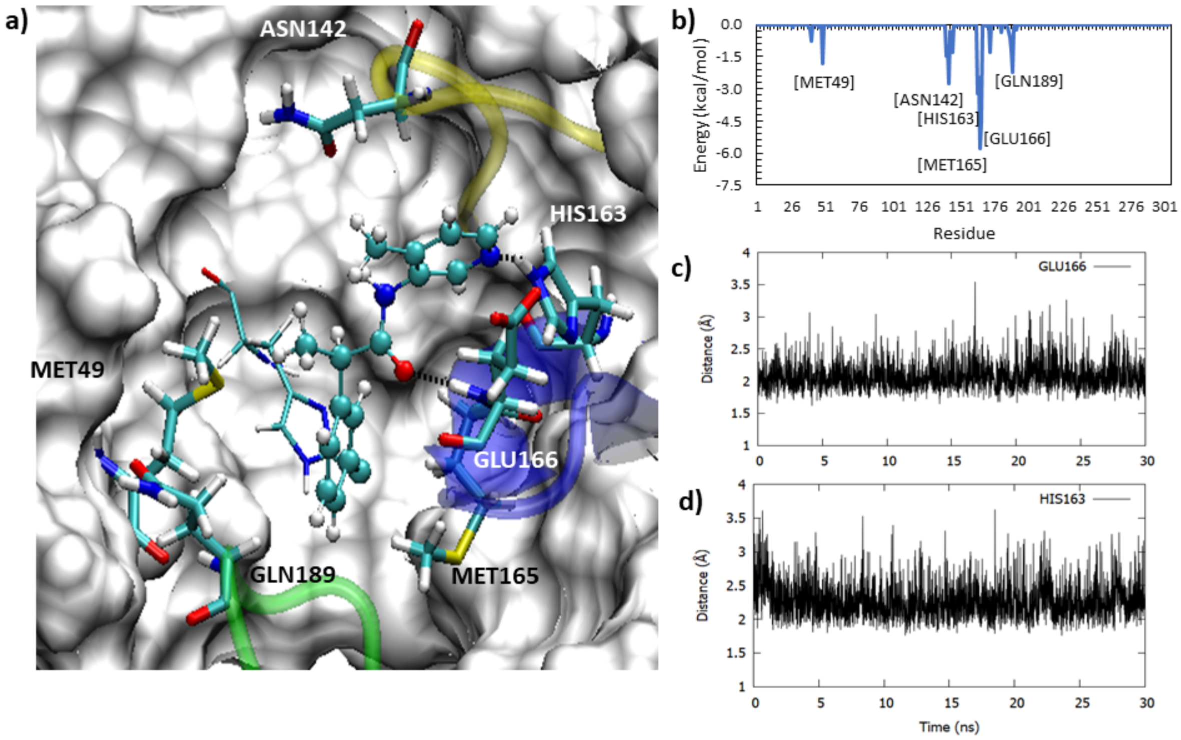

SFig. 12. Diagram representing the predominant binding pose of the ligand in 5R81(a) with a binding affinity of -18.35 kcal/mol and associated energy impacts from key residues in the decomposition graph (b) as well as hydrogen bond evolution plot with GLN192(c). The ligand forms Van der Waals interactions with three of the four main regions of the active site as seen in the interaction diagram and in the energy, peaks corresponding to these residues in (b) namely HIS41, MET49, MET165, GLU166, GLN189. The oxygens of the sulfonamide group form a combined stable hydrogen bond with the amino group of GLN192 while the terminal amino moiety forms hydrogen bonds with THR190 in the latter half of the simulation. Images/graphs were created using VMD 1.9.3[1](a), GNUplot (v5.2 patchlevel 8 http://www.gnuplot.info/)(c), Microsoft Excel 365 (https://www.office.com/)(b).

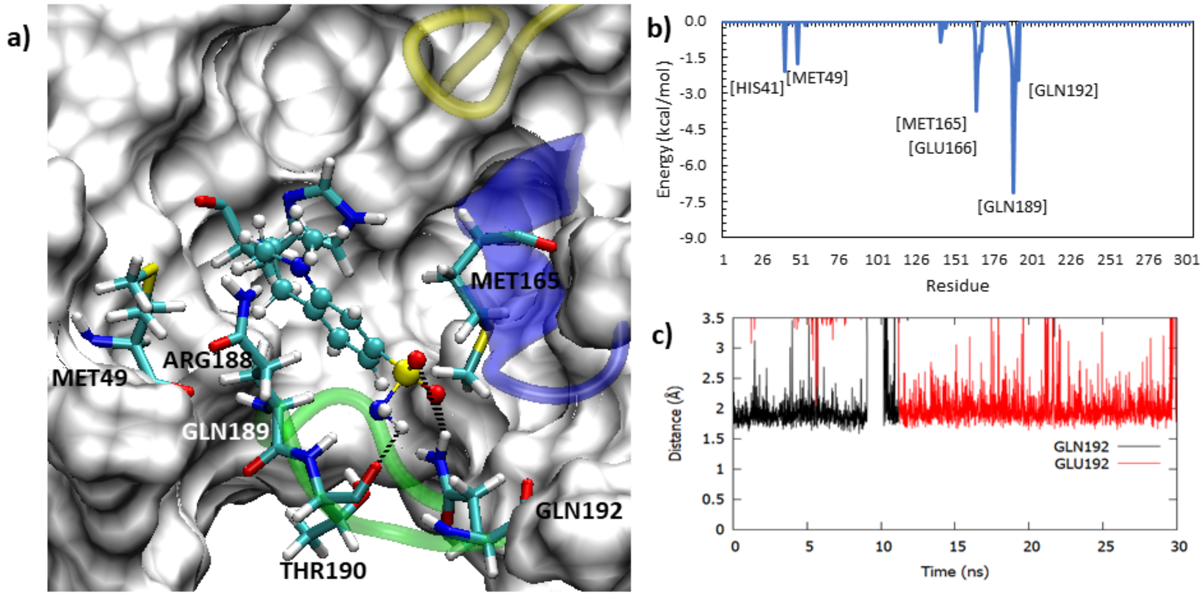

SFig. 13. Interaction diagram of ligand in complex 5RF7 (a) alongwith its pairwise decomposition graph in (b) and impactful hydrogen bonding evolution plots in (c) and (d). The ligand has a binding affinity of and is clearly occupying the lateral pocket and forming a stable hydrogen bond with HIS163 (a,c) but the rest of the ligand is not stabilised evidenced by fluctuating hydrogen bonds with GLY143, ASN142 and GLU166 as well as the lack of interactions with other main regions of the active site. Images/graphs were created using VMD 1.9.3[1](a), GNUplot (v5.2 patchlevel 8 http://www.gnuplot.info/)(c,d), Microsoft Excel 365 (https://www.office.com/)(b).

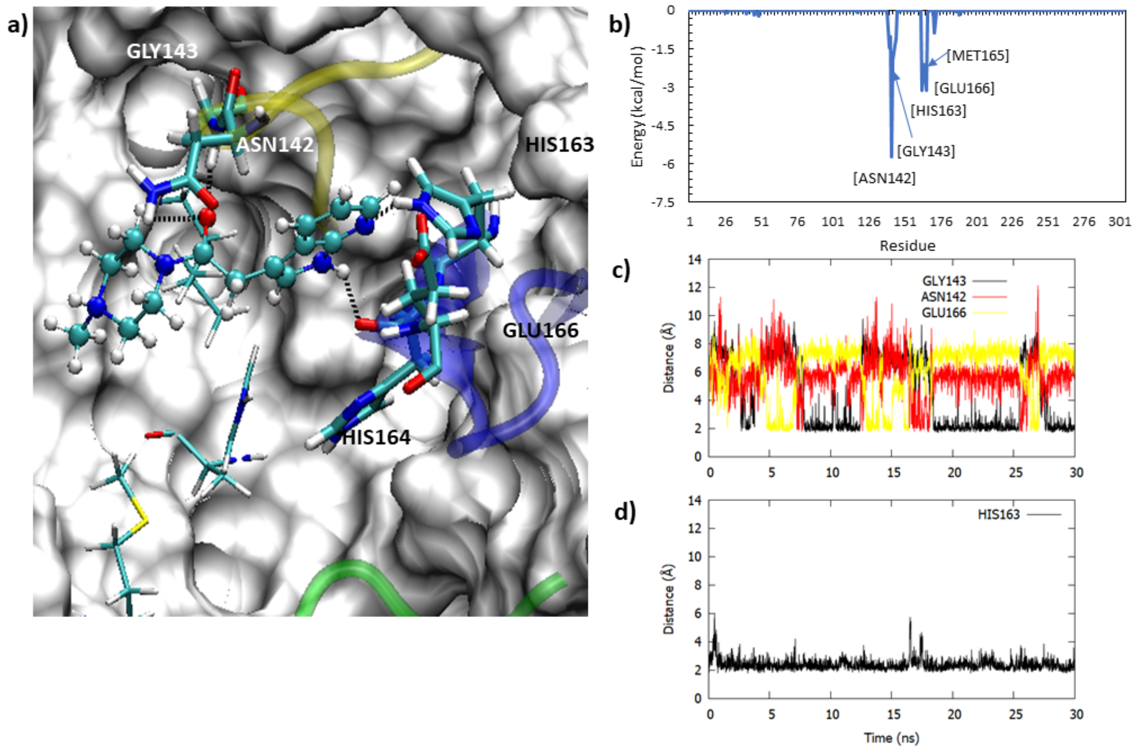

SFig. 14

A scatter plot comparing the binding affinity of the selected ligand-M^{pro} complexes calculated using the 30 ns and 100 ns MD trajectories. This graph was created using Microsoft Excel 365 (https://www.office.com/).

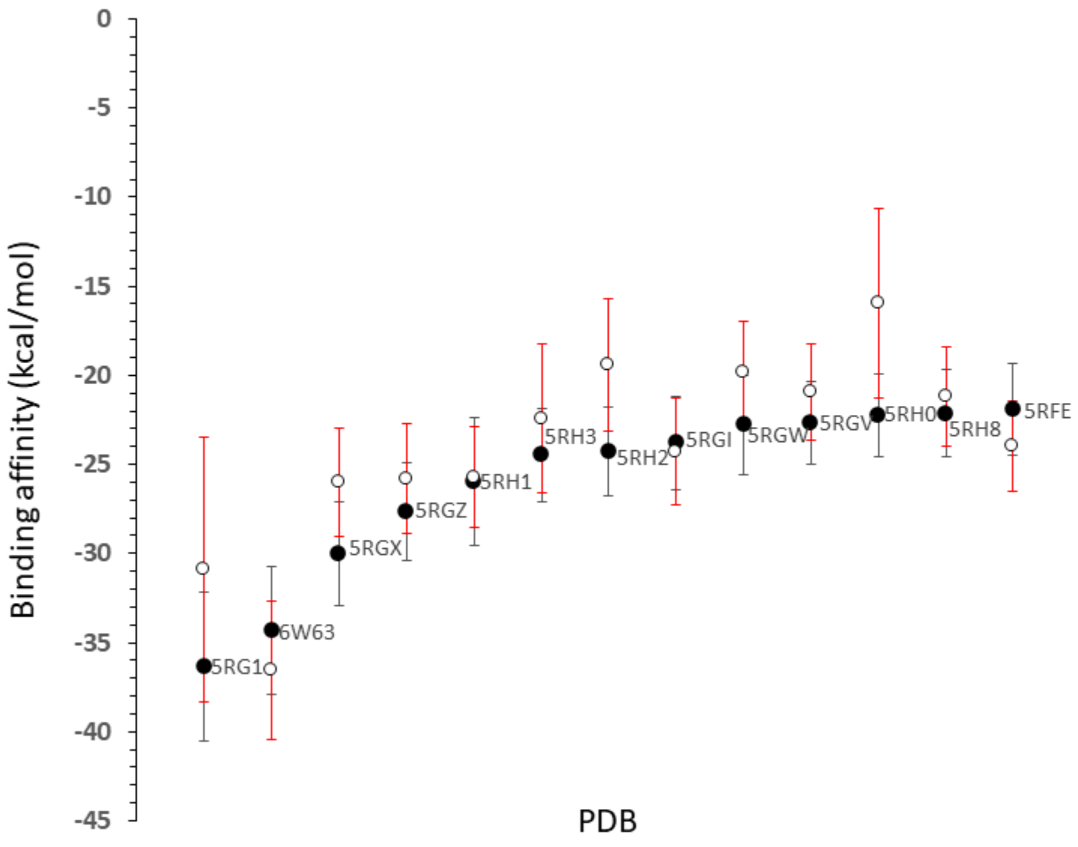

SFig. 15. (a) ***Time-course mobility of the combined ensemble.*** Root median square displacement, RMSD (in Å), of 75,000 iterposed conformations of the C_α-trace of Covid19-SARS-2 Mpro from the average conformation of a combined trajectory (Grand ensemble) of fifteen 100 ns MD simulations (5k conformations each), and colored by trajectory according to 0: 6M2Q (black), 1: 5RG1 (red), 2: 6W63 (blue), 3: 5RGX (green), 4: 5RGZ (indigo), 5: 5RH1 (dark cyan), 6: 5RH3 (purple), 7: 5RH2 (sienna), 8: 5RGI (dark olive), 9: 5RGW (teal), 10: 5RGV (magenta), 11: 5RH0 (brown), 12: 5RH8 (gold), 13: 5RFE (lime), and 14: 5RE9 (navy). (b) *Average mobility of individual trajectories on the combined ensemble*. Average RMSD (in Å) of 5,000 conformations per trajectory vs index of each trajectory. (c) *Mobility profile of the combined ensemble*. Mean square fluctuation, MSqF (in $Å^2$) from the Grand ensemble of MD conformations vs Mpro residue index (5..300). Highlighted with colored dots residues belonging to active-site loops according to segment [24-28] (red), L3: [39-54] (blue), L_3: [118-119] (green), L1: [140-146] (cyan), "L" strand: [163-172] (purple), and L2: [181-192] (yellow). L_{dIII}: [275,292] is the linker between H10 and H11 at D_{III}. Graphs were created using Microsoft Excel 365 (https://www.office.com/).

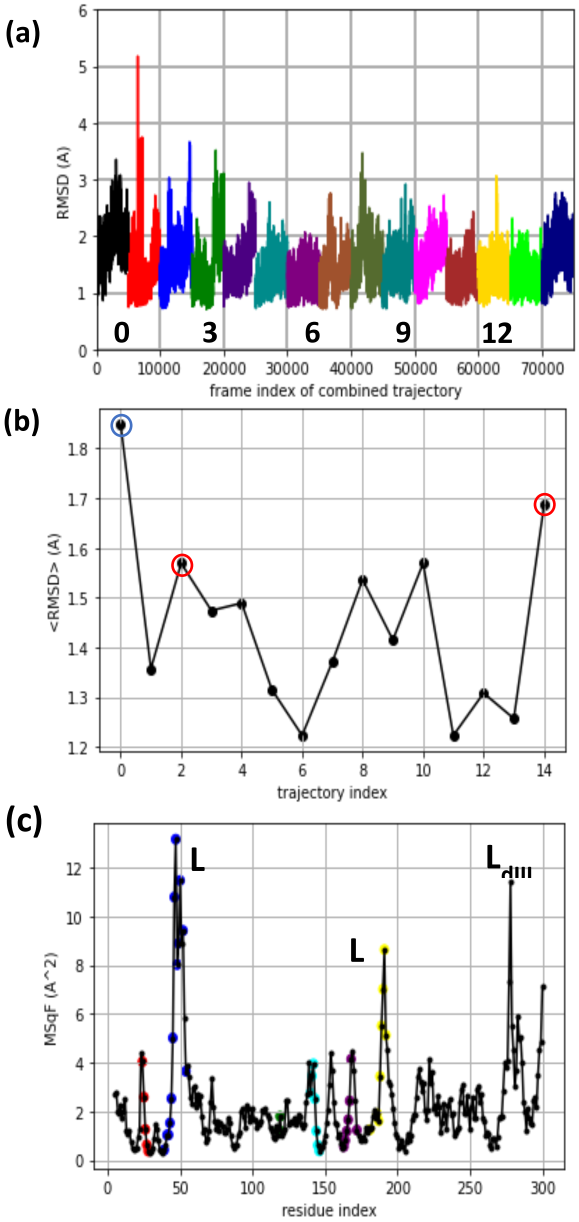

SFig. 16 ***Residue mobility profile per trajectory*.** Each panel shows the MSqF (in Å^2) for 191 C$_\alpha$-atoms ($D_{I/II}$) from each ensemble of MD conformations vs M^{pro} residue index (from 5 to 195). Highlighted with colors residues belonging to active-site loops as indicated in SFig.15c. Graphs were created using Microsoft Excel 365 (https://www.office.com/).

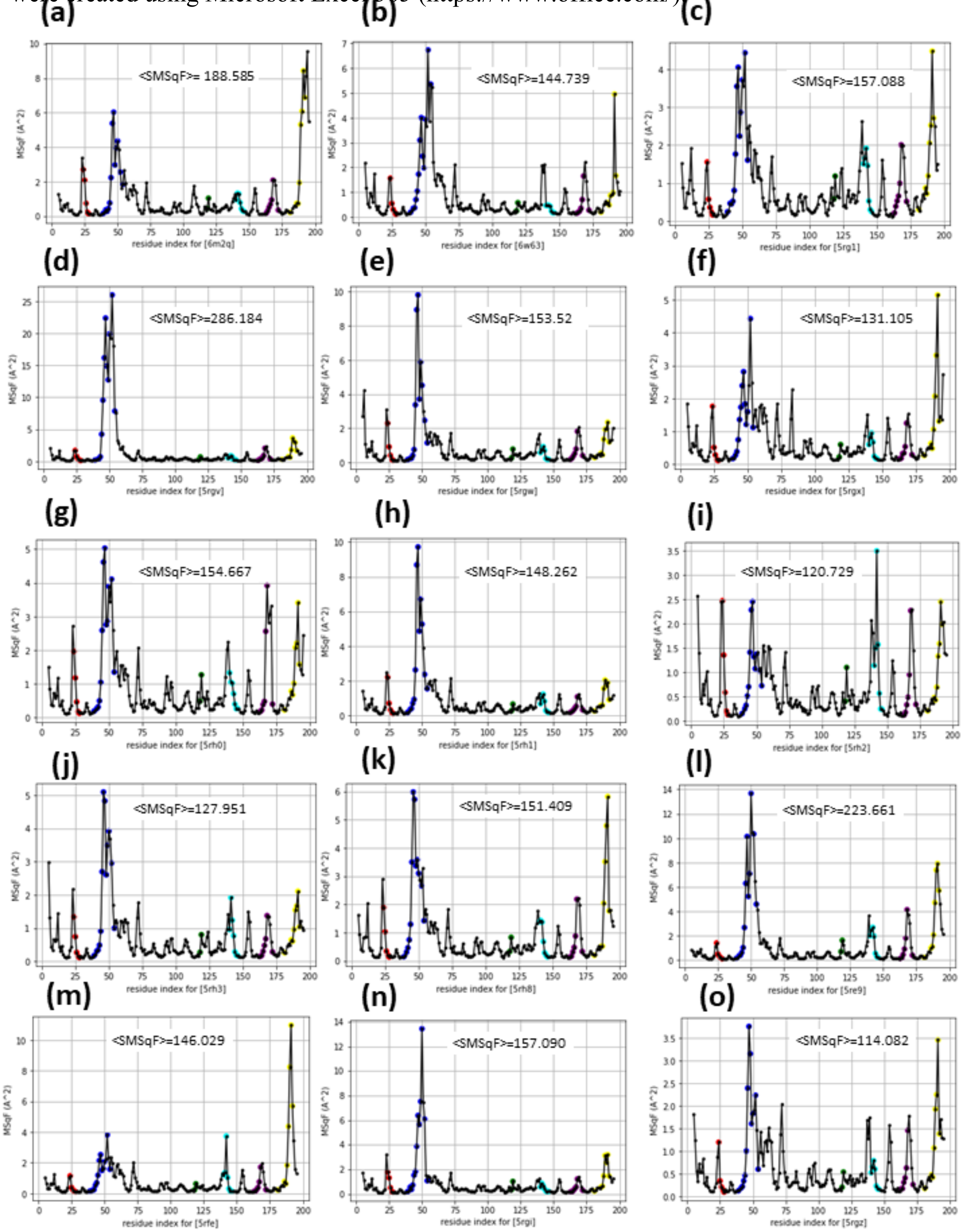

SFig.17. ***Relevance of the essential space of each trajectory.*** Each panel shows the RMSF (in Å) for 191 C_{α}-atoms ($D_{I/II}$) from each ensemble of MD conformations vs M^{pro} residue index (from 5 to 195) for all modes (black line), PC1 (blue), PC[1:4] (red), and PC[1:20] (green).

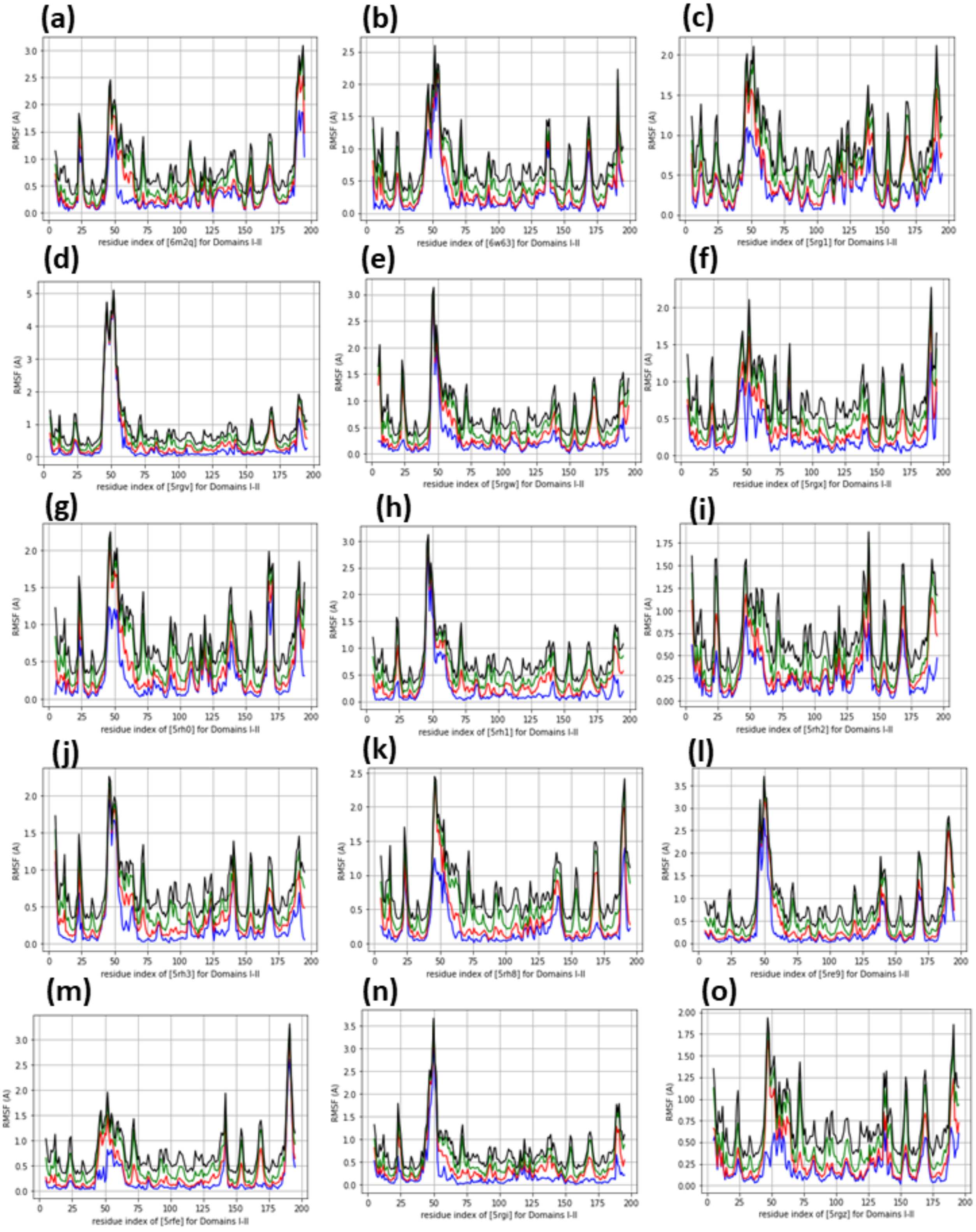

SFig. 18. ***Similitude between essential subspaces.*** Subspaces overlap heatmap matrix (from green to red) between the first 4 PC modes of an EDA on the MD ensemble indexed by idx1 (columns) and the 3 PC modes of an EDA on the MD ensemble indexed by idx2 (rows). In both cases considering only 191 Cα-trace (DI/II) of Mpro (residues 5..195). Graphs were created using Microsoft Excel 365 (https://www.office.com/).

	idx1	0	1	2	3	4	5	6	7	8	9	10	11	12	13	14	
idx2	pdb	6m2q	5rg1	6w63	5rgx	5rgz	5rh1	5rh3	5rh2	5rgi	5rgw	5rgv	5rh0	5rh8	5rf3	5re9	average
0	6M2Q	1.000	0.627	0.262	0.496	0.613	0.656	0.694	0.740	0.609	0.724	0.575	0.651	0.659	0.558	0.690	0.637
1	5RG1	0.542	1.000	0.318	0.484	0.501	0.526	0.608	0.599	0.518	0.567	0.654	0.481	0.542	0.462	0.599	0.560
2	6W^3	0.288	0.389	1.000	0.366	0.436	0.349	0.378	0.363	0.310	0.339	0.453	0.349	0.388	0.289	0.474	0.411
3	5RGX	0.460	0.432	0.311	1.000	0.571	0.379	0.482	0.476	0.413	0.453	0.540	0.444	0.611	0.389	0.503	0.498
4	5RGZ	0.618	0.578	0.366	0.603	1.000	0.561	0.658	0.669	0.595	0.647	0.617	0.624	0.598	0.547	0.647	0.622
5	5RH1	0.677	0.640	0.296	0.371	0.538	1.000	0.644	0.602	0.697	0.745	0.548	0.630	0.575	0.635	0.643	0.616
6	5RH3	0.638	0.666	0.313	0.520	0.622	0.633	1.000	0.610	0.610	0.688	0.579	0.613	0.579	0.451	0.629	0.610
7	5RH2	0.659	0.599	0.356	0.568	0.655	0.538	0.614	1.000	0.530	0.622	0.622	0.629	0.585	0.572	0.642	0.613
8	5RGI	0.653	0.653	0.338	0.450	0.600	0.735	0.635	0.593	1.000	0.730	0.507	0.728	0.539	0.614	0.769	0.636
9	5RGW	0.691	0.673	0.342	0.490	0.579	0.738	0.703	0.651	0.671	1.000	0.526	0.679	0.655	0.565	0.678	0.643
10	5RGV	0.525	0.575	0.428	0.625	0.598	0.497	0.577	0.600	0.497	0.605	1.000	0.502	0.662	0.479	0.655	0.588
11	5RH0	0.630	0.548	0.336	0.450	0.540	0.636	0.619	0.614	0.692	0.673	0.435	1.000	0.514	0.568	0.654	0.594
12	5RH8	0.638	0.601	0.383	0.603	0.596	0.580	0.609	0.589	0.556	0.672	0.636	0.523	1.000	0.498	0.663	0.610
13	5RF3	0.572	0.437	0.243	0.428	0.490	0.585	0.393	0.574	0.521	0.548	0.457	0.517	0.448	1.000	0.647	0.524
14	5RE9	0.657	0.693	0.483	0.551	0.642	0.626	0.661	0.643	0.687	0.693	0.628	0.645	0.669	0.650	1.000	0.662
	average	0.617	0.607	0.385	0.534	0.599	0.603	0.618	0.622	0.594	0.647	0.585	0.601	0.602	0.552	0.660	

SFig. 19. Wildtype 5RGX complex interactions (a), 5RGX HIS163ALA mutant complex interactions (b), and pairwise residue decomposition graph depicting residue contribution in 5RGX wildtype and mutant complexes (c). Representative snapshots of key interactions near the pyridine ring are shown in Fig.X(a) and (b). The wildtype complex forms strong electrostatic interactions with HIS163, GLY143, SER144, and CYS145. The mutant complex loses electrostatic interactions with the pyridine ring and the double bonded oxygen atom is shifted to form transient interactions with CYS145 and GLU166. The difference in residue contribution to overall MMGBSA binding affinity is shown via the wildtype and mutant pairwise decomposition graphs where the residue denoted by an asterisk (*) represents HIS163ALA. Images/graphs were created using VMD 1.9.3 [1](a,b) and Microsoft Excel 365(c) (https://www.office.com/).

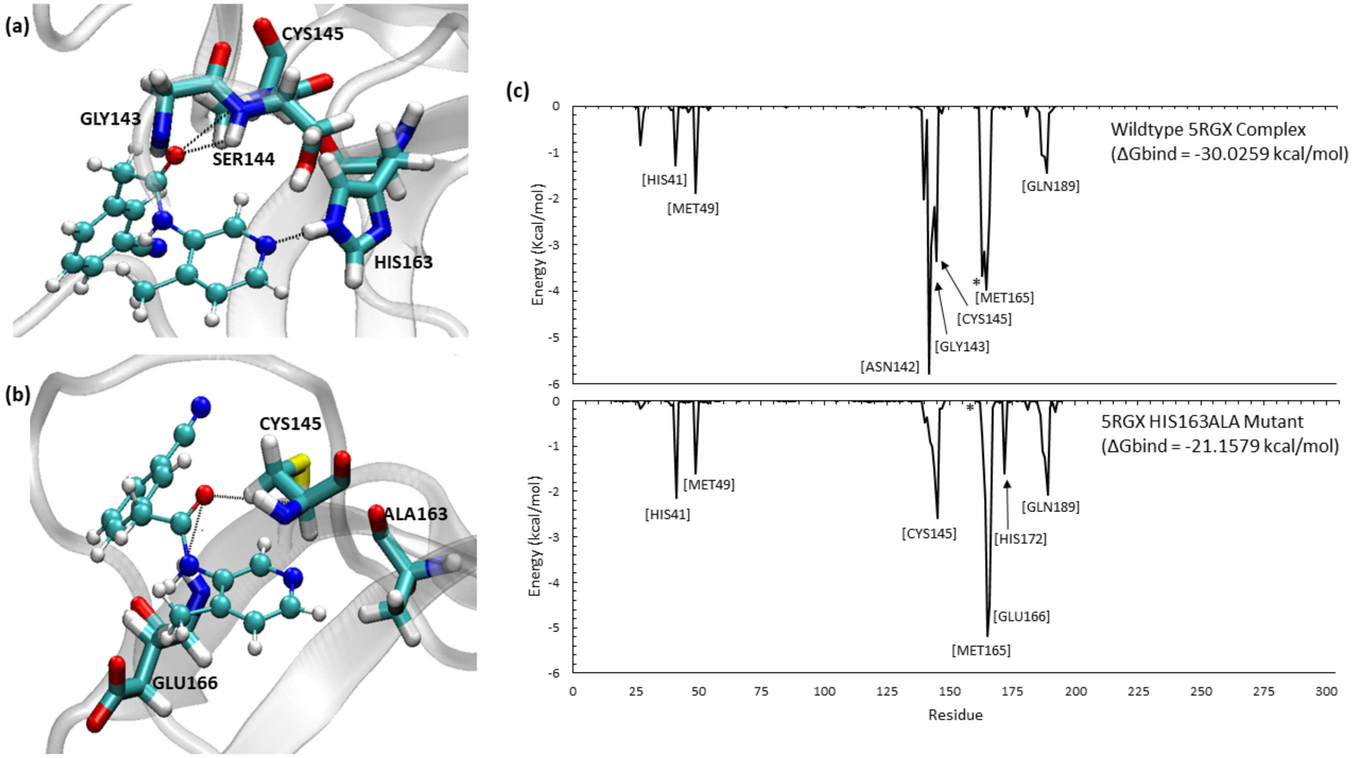

SFig. 20. Representative snapshot of the 5RGZ HIS163ALA mutant complex at 0 ns and 15ns (a), and pairwise residue decomposition graph depicting residue contribution in 5RGZ wildtype and mutant complexes (b). The initial binding poses of the 5RGZ HIS163ALA mutant complex (red) reveals the pyridine ring located close to ALA163 in the lateral pocket. After 15 ns, the pyridine ring rotates out of the binding pocket (blue), greatly decreasing its MMGBSA binding affinity. The difference in residue contribution to overall MMGBSA binding affinity is shown via the wildtype and mutant pairwise decomposition graphs where the residue denoted by an asterisk (*) represents HIS163ALA. Images/graphs were created using VMD 1.9.3 [1](a) and Microsoft Excel 365(b) (https://www.office.com/).

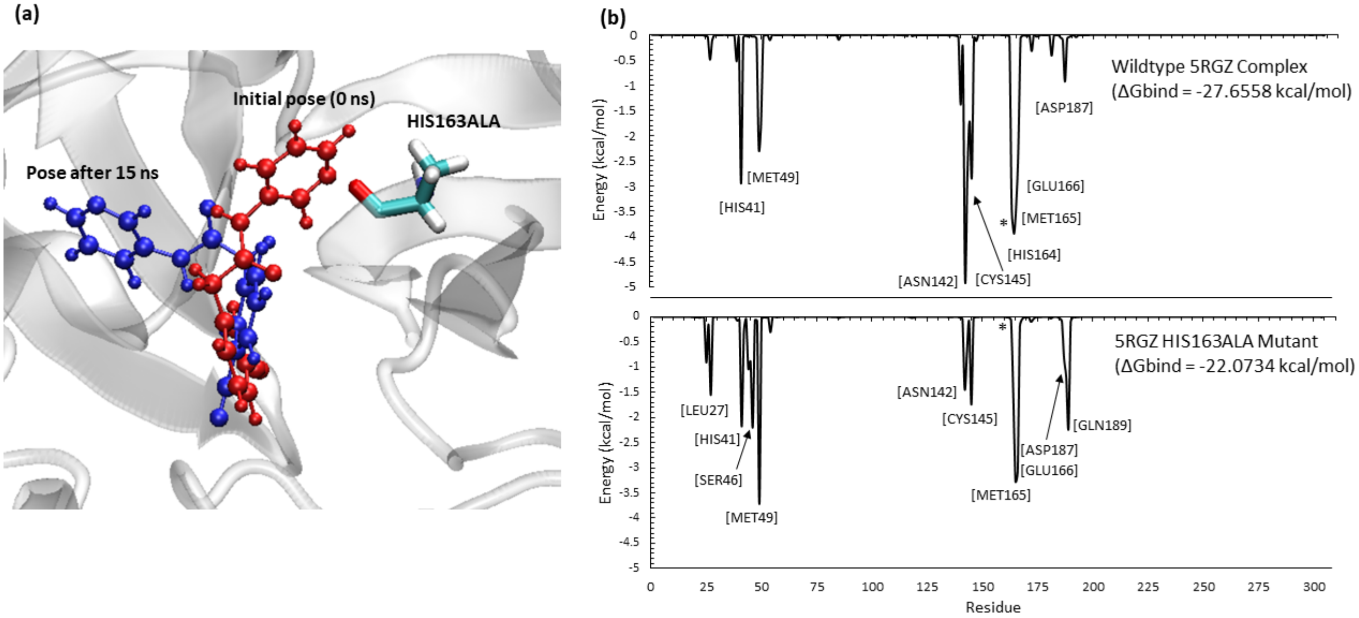

SFig. 21. Wildtype 5RG1 complex interactions (a), 5RG1 HIS163ALA mutant complex interactions (b), and pairwise residue decomposition graph depicting residue contribution in 5RG1 wildtype and mutant complexes (c). The wildtype 5RG1 complex shows stable interactions with key residues HIS163, GLU166, and GLN189. In the HIS163ALA 5RG1 mutant complex, the HIS163 strong, electrostatic interaction is replaced by an additional, but transient, electrostatic interaction with GLU166. The alanine mutation also causes the GLN189 to be lost. The difference in residue contribution to overall MMGBSA binding affinity is shown via the wildtype and mutant pairwise decomposition graphs where the residue denoted by an asterisk (*) represents HIS163ALA. Images/graphs were created using VMD 1.9.3 [1](a,b) and Microsoft Excel 365(c) (https://www.office.com/).

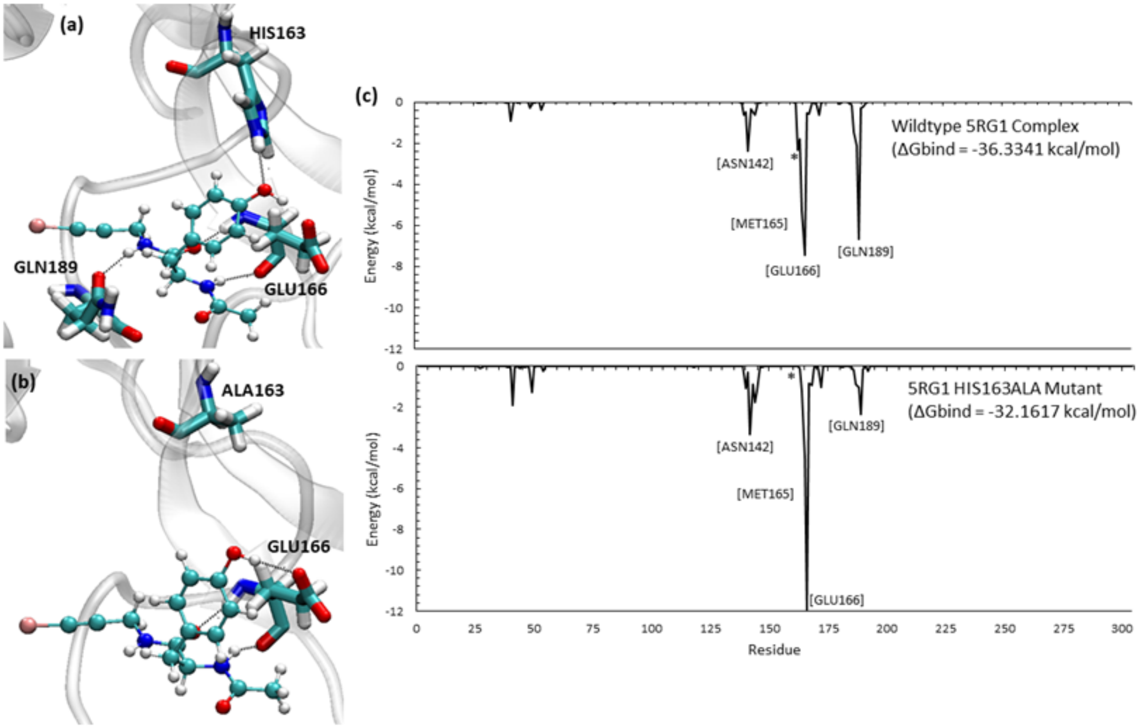

SFig.22. Two representations of key wildtype 6W63 complex interactions (a), 6W63 HIS163ALA mutant complex interactions near pyridine ring (b), and pairwise residue decomposition graph depicting residue contribution in 6W63 wildtype and mutant complexes (c). The wildtype 6W63 complex contains a large ligand that forms many interactions with different residues to maintain its position in the binding site. Some of these key residues include HIS41, ASN142, GLY143, HIS163, and GLU166. Due to its large ligand size, the HIS163ALA 6W63 mutant complex is still relatively stable, but the pyridine ring in the lateral pocket and nearby double-bonded oxygen atoms do undergo some changes in the interactions they form with the protein. For example, the nitrogen atom of the pyridine ring loses its stable interaction with HIS163 to instead form an electrostatic interaction with CYS145. The difference in residue contribution to overall MMGBSA binding affinity is shown via the wildtype and mutant pairwise decomposition graphs where the residue denoted by an asterisk (*) represents HIS163ALA. Images/graphs were created using VMD 1.9.3 [1](a,b) and Microsoft Excel 365(c) (https://www.office.com/).

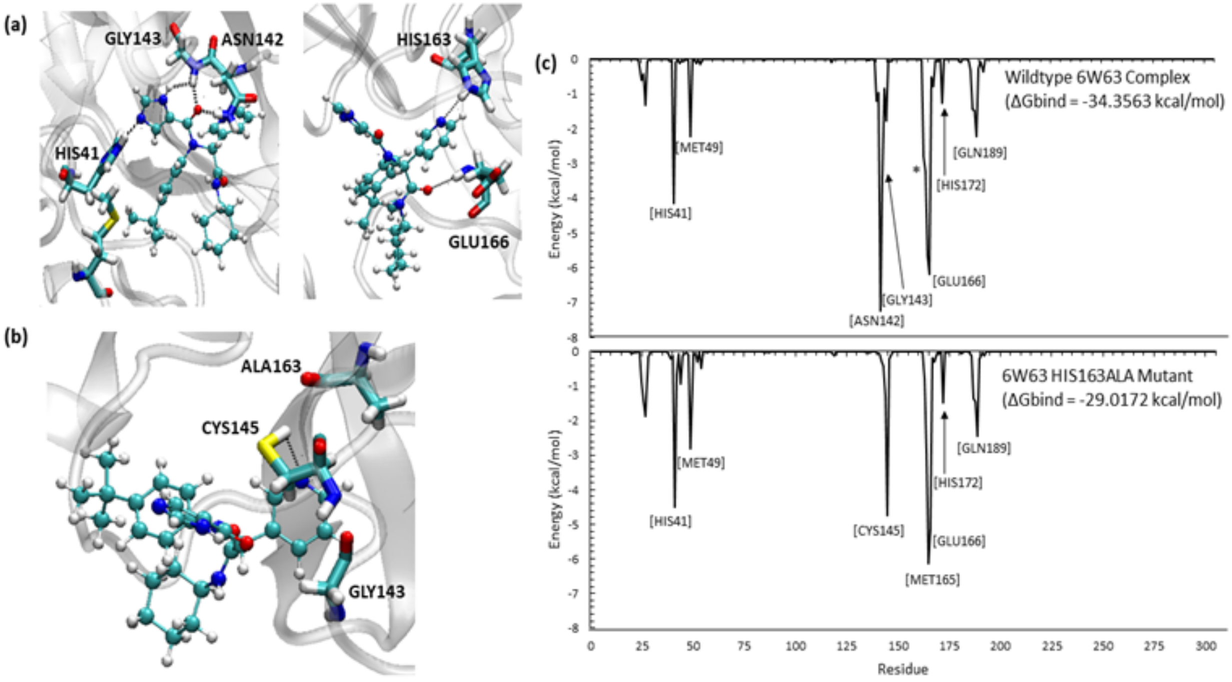

SFig. 23. Wildtype 5RF7 complex interactions (a), 5RF7 HIS163ALA mutant complex interactions (b), and pairwise residue decomposition graph depicting residue contribution in 5Rf7 wildtype and mutant complexes (c). The 5RF7 complex contains a unique 7-azaindole double-ring structure positioned in the lateral pocket. This enables two strong hydrogen bonds with HIS163 and HIS164 to be present in the wildtype 5RF7 complex, shown in Fig.Z.a. When the complex is mutated from a histidine to alanine at reside 163, the hydrogen bond with the 6-membered ring of the 7-azaindole group is lost, but the hydrogen bond between HIS164 and the 5-membered ring is maintained. This interaction stabilizes the HIS163ALA mutant complex in the lateral binding pocket and reduces the MMGSBA difference between the wildtype and mutated complexes. The difference in residue contribution to overall MMGBSA binding affinity is shown via the wildtype and mutant pairwise decomposition graphs where the residue denoted by an asterisk (*) represents HIS163ALA. Images/graphs were created using VMD 1.9.3 [1](a,b) and Microsoft Excel 365(c) (https://www.office.com/).

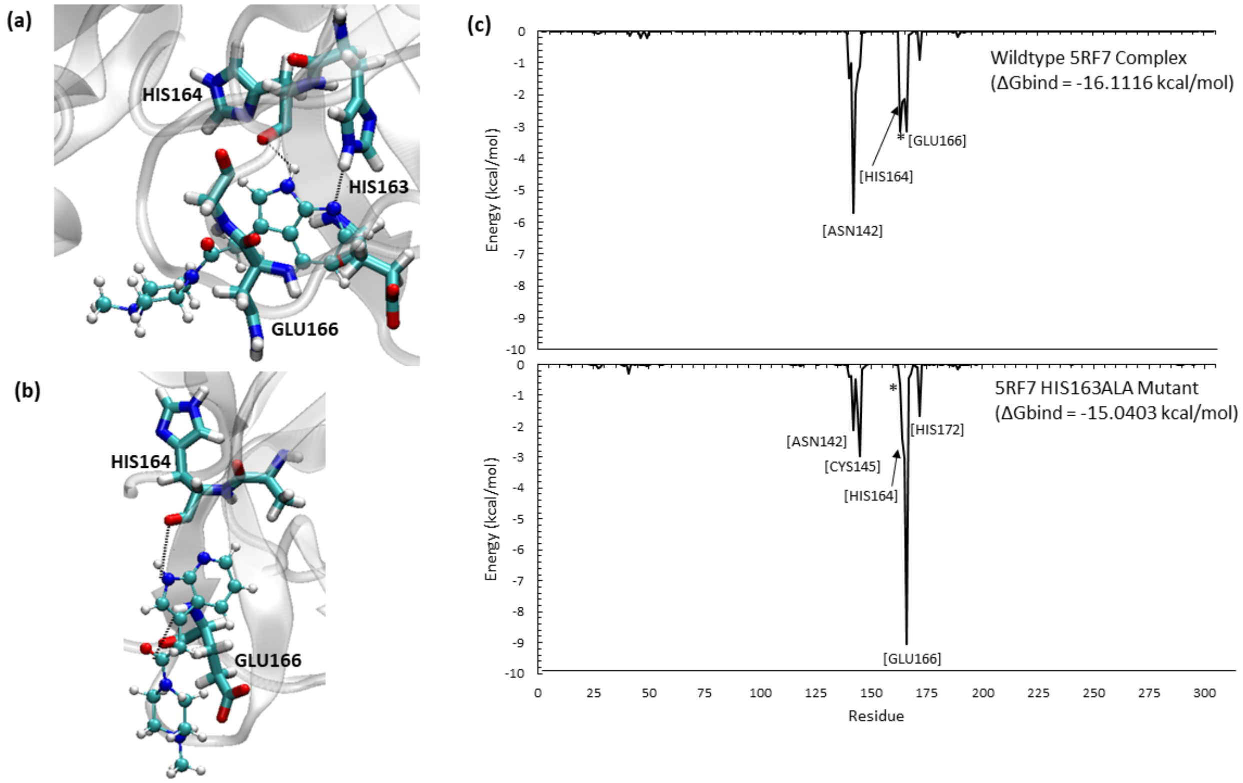

SFig. 24. Some unfavourable NWATER interactions in complexes showing a negative energy impact of water. 2-3 water forms unfavorable interaction with benzene ring of 5RGX and 5RE9 at polar region of M^{pro} (e-f). Images were created using VMD 1.9.3 [1](a,b)

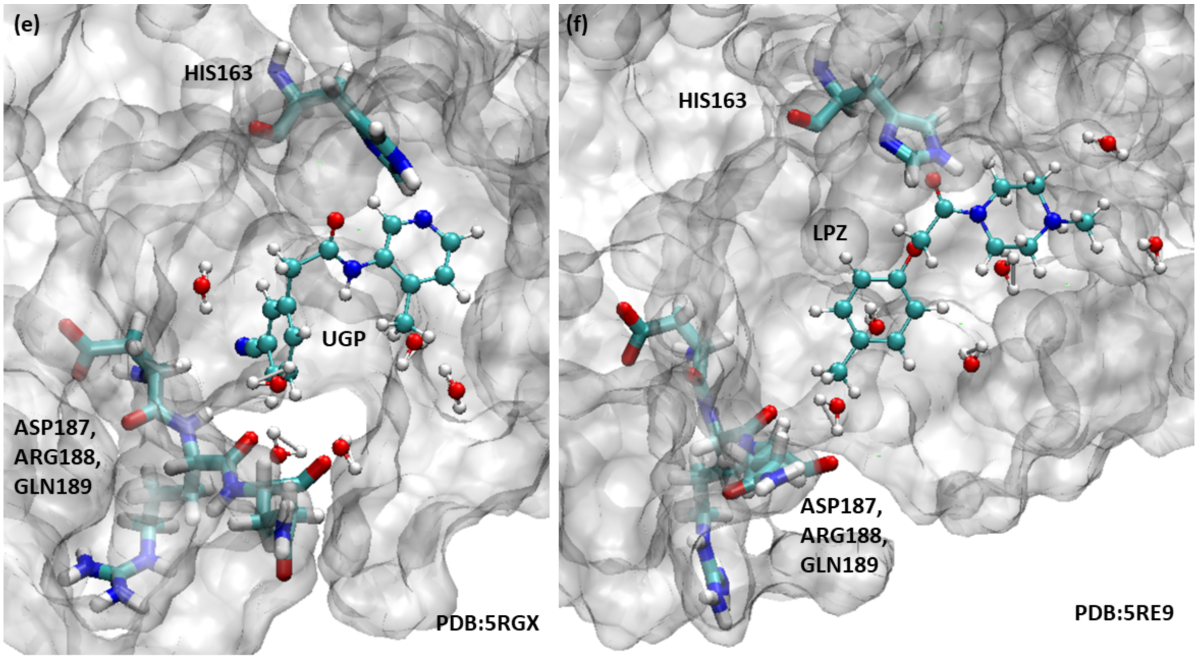

SFig. 25. A surface representation of a dimer model of M^{pro} (red surface is monomer 1 and transparent surface is monomer 2) showing the interactions of ligands (shown as spheres) that are bound at different sites at proximity to the dimer interface. The PDB codes of the ligands are marked. The ligands binding at the dimer interface is marked by *. The representation below was created in VMD 1.9.3[1].

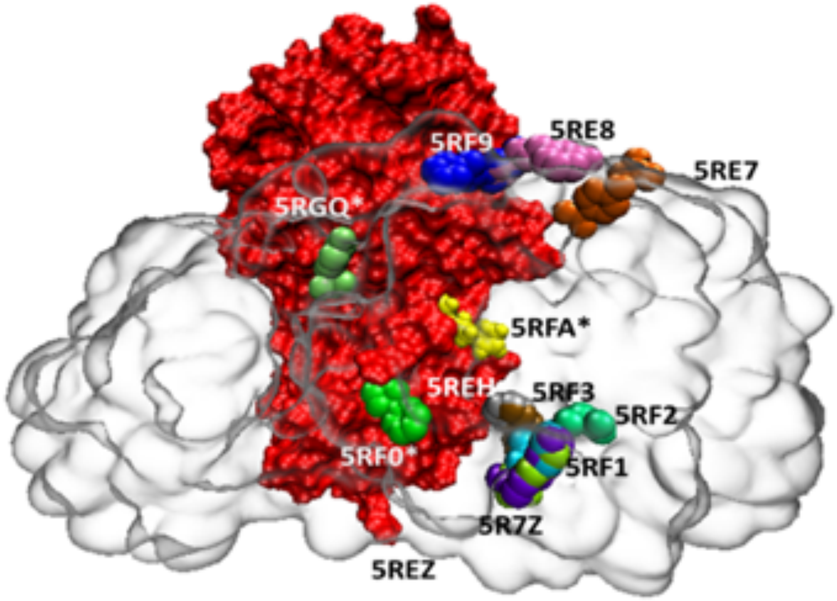

SFig. 26. The binding of ligand from 5RFA within a dimer model of M^{pro} (shown as surface representation) (a) shown, along with the fluctuation of the ligand when bound with the monomer and dimer (b), the pairwise energy decomposition plot (c) and the binding pose of the ligand within the dimer (d). The RMSD plots describes that the ligand was unstable when bound to a monomer of M^{pro}; however, it was more stable within a dimer condition although it underwent some conformational changes at ~10ns before reaching a plateau indicating the stability of the ligand after this point (b). The energy decomposition analyses (c) identified a number of key residues from both the M^{pro} monomer chains to stabilize the ligand interactions, as described in the 2D interaction diagram (d).Images/graphs were created using VMD 1.9.3[1](a), GNUplot (v5.2 patchlevel 8 http://www.gnuplot.info/)(b), Microsoft Excel 365 (https://www.office.com/)(c), Schrodinger Maestro 2019-04(d)[2].

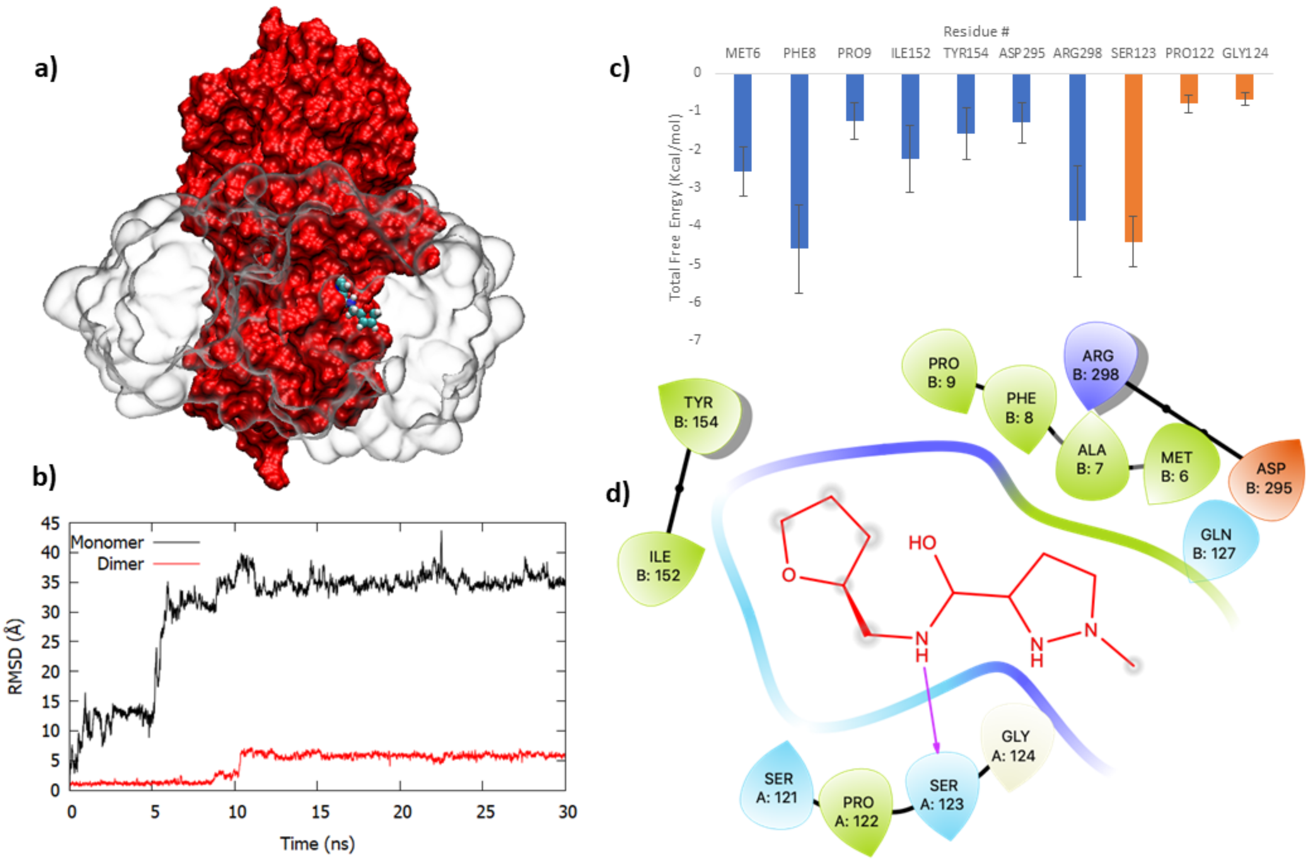

SFig. 27. Binding Site representations and RMSD for 11 of the 13 ligands simulated in the M^{pro} dimer model. The following 11 figures are titled with the original crystal structure complex and they showcase the initial binding site of each compound in the dimer in (a) of each figure and the unaligned ligand RMSD throughout the 30ns timescale for both the monomer and dimer simulations are compared in the graphs shown in (b). It can be clearly seen that some ligands definitely benefit from the dimer model while most ligands in this list have not had a significant impact and have egressed from the surface of M^{pro} in both the models. Each molecular graphic was generated in VMD 1.9.3 while graphs were made in GNUplot (v5.2 patchlevel 8 http://www.gnuplot.info/).

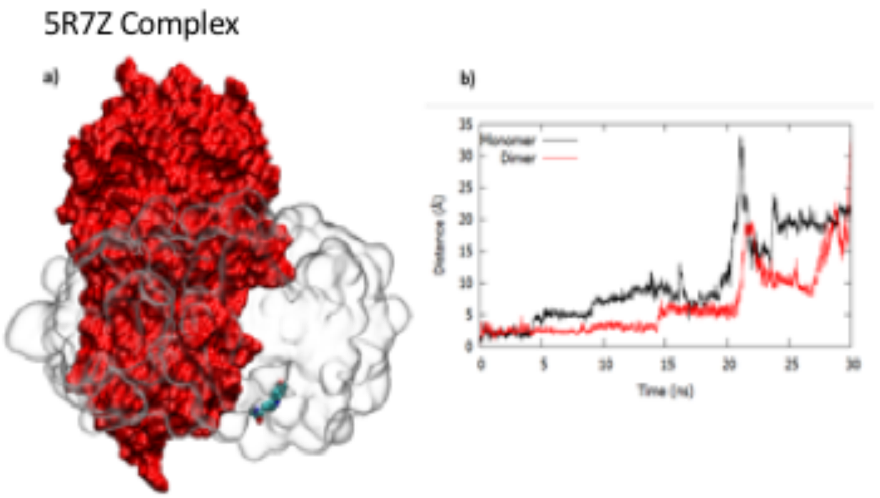

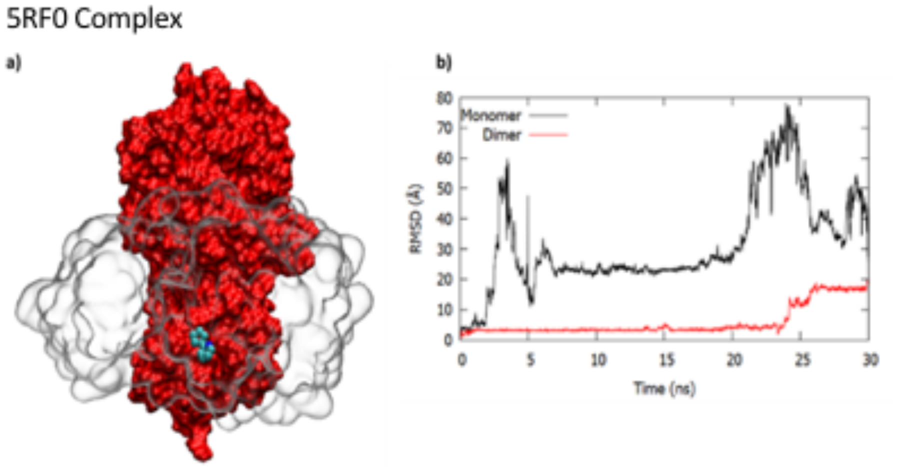

5RE8 Complex

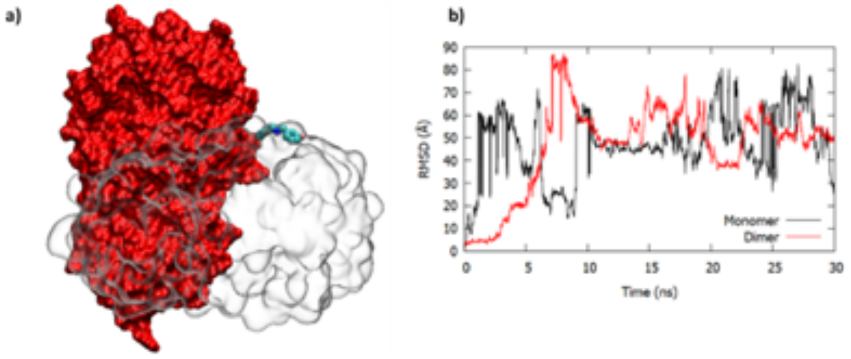

5RE7 Complex

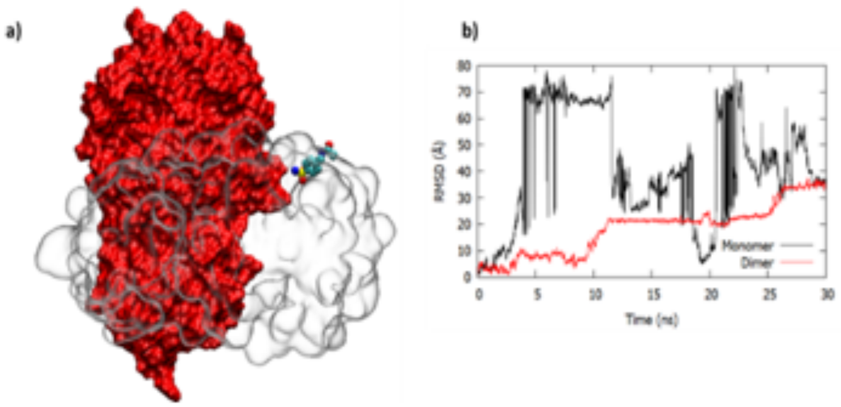

5REZ Complex

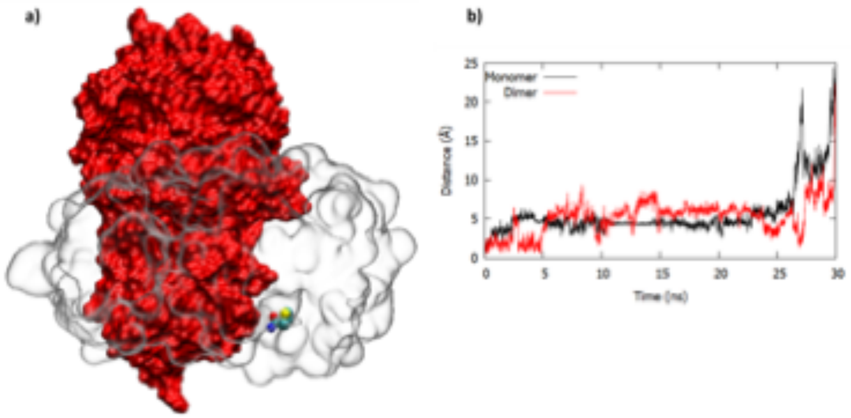

5RF1 Complex

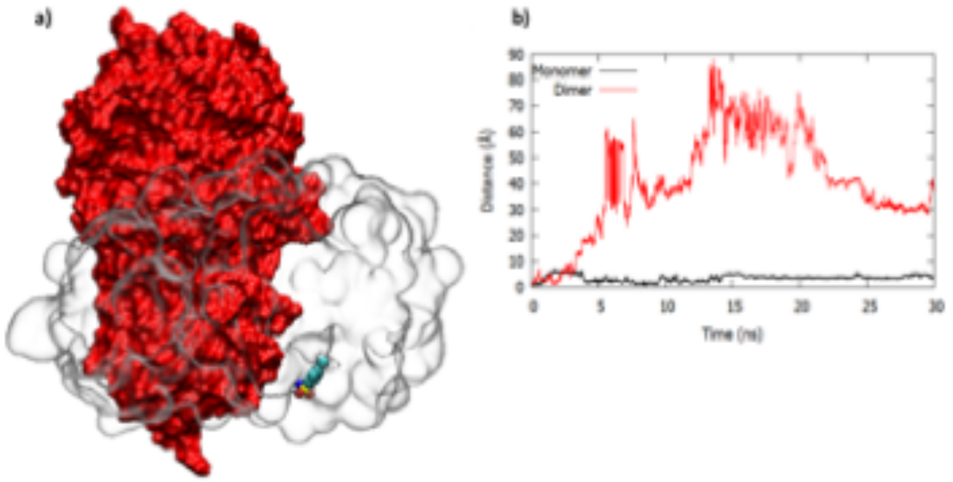

5RF2 Complex

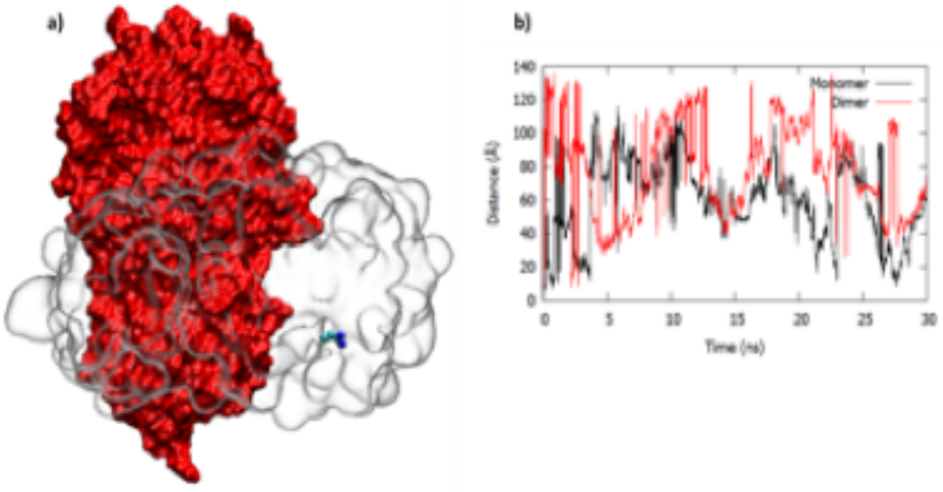

5RF3 Complex

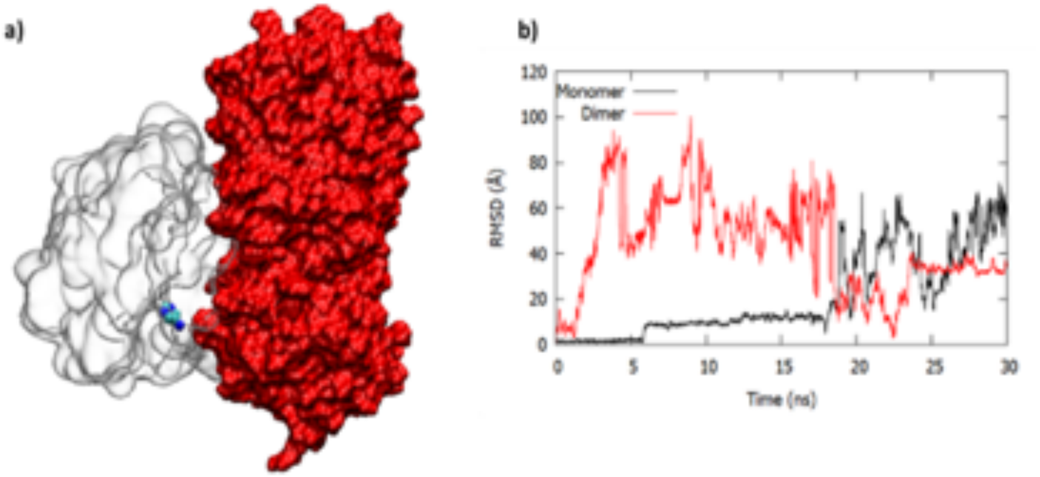

5RF9 Complex

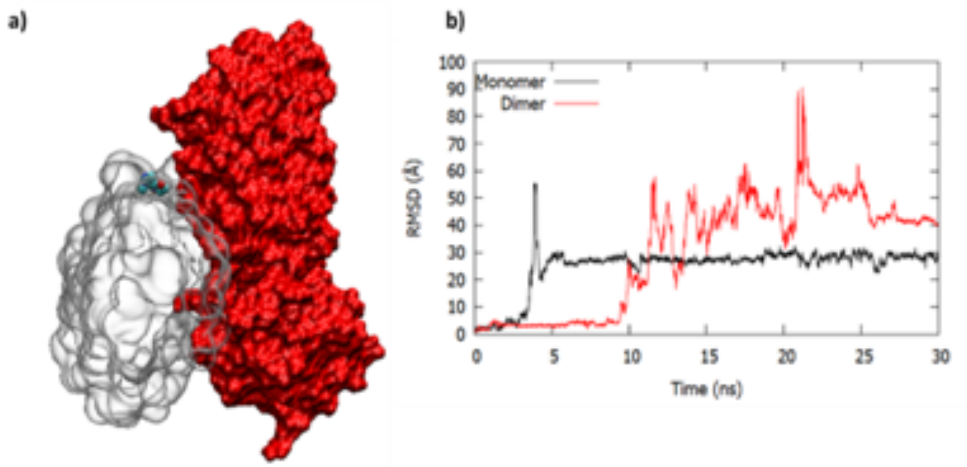

5RGJ Complex

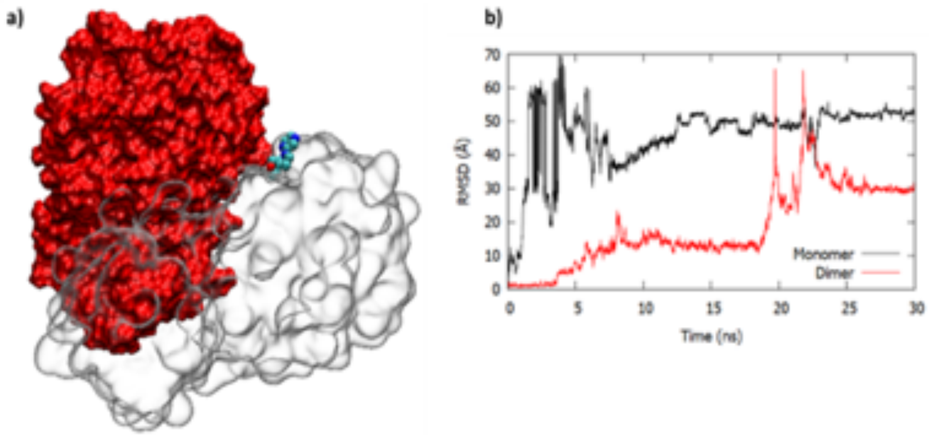

5RGQ Complex

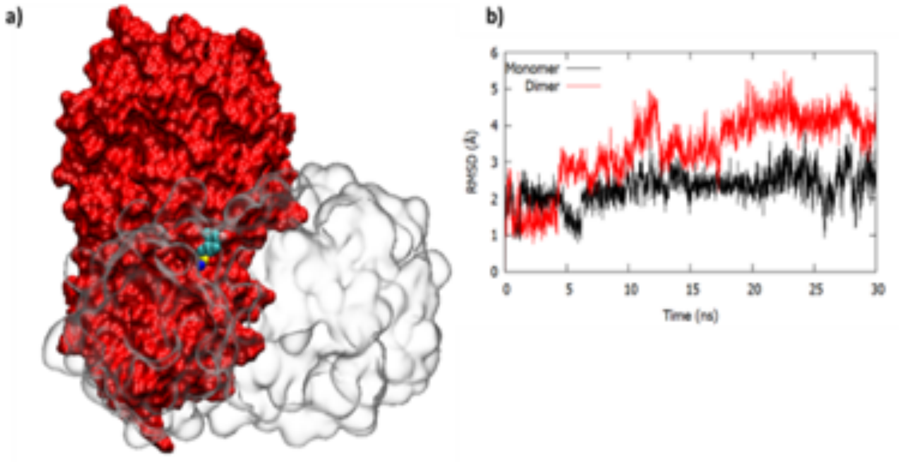

Supplement: Supplementary file 1 — Supplementary Information. [file 41598_2021_86471_MOESM1_ESM.pdf]
